# Next-generation disulfide stapling: reduction and functional re-bridging all in one†
†Electronic supplementary information (ESI) available: ^1^H and ^13^C NMR spectra for all small molecules, LC-MS, SDS-PAGE gels and UV-Vis analysis (where applicable) for all bioconjugates. See DOI: 10.1039/c5sc02666k



**DOI:** 10.1039/c5sc02666k

**Published:** 2015-09-15

**Authors:** Maximillian T. W. Lee, Antoine Maruani, James R. Baker, Stephen Caddick, Vijay Chudasama

**Affiliations:** a Department of Chemistry , University College London , 20 Gordon Street , London , WC1H 0AJ , United Kingdom . Email: v.chudasama@ucl.ac.uk ; Tel: +44207 679 2077

## Abstract

Herein we present a significant step towards next-generation disulfide stapling reagents. A novel class of reagent has been designed to effect both disulfide reduction and functional re-bridging. The strategy has been applied to great success across various peptides and proteins. Moreover, application to a multi-disulfide system resulted in functional re-bridging without disulfide scrambling.

## Abstract 2

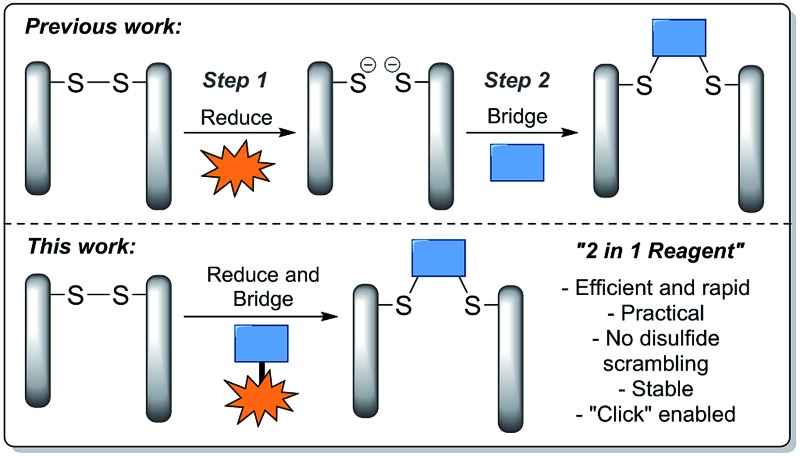
A next-generation disulfide stapling reagent, incorporating both reducing and re-bridging functions, is shown to be successful across various proteins.

## 

Advances in protein modification by chemical means have led to the development of a range of protein bioconjugation methodologies.^1^ These methodologies have been successfully applied to a number of fields such as the fluorescent tagging of proteins,^2^ the development of therapeutic protein conjugates^3,4^ to treat indications such as HIV,^5^ cancer^6^ and malaria,^7^ and for use as diagnostic tools.^8^

Whilst a large number of reagents and protocols have been developed to modify proteins, novel strategies for site-specific conjugation continue to attract considerable interest in view of the increasingly stringent requirements in the development of biologics.^1^ Several amino acid side-chains have been targeted for site-selective modification over the past few decades, *e.g.* tryptophan, histidine, tyrosine and, in particular, lysine and cysteine. However, the modification of native disulfides through functional re-bridging, pioneered by Brocchini *et al.*, has attracted significant interest in recent years with various approaches being developed.^9–13^

Recently, Chudasama, Caddick *et al.* have shown dibromopyridazinediones to be viable candidates for disulfide stapling and that the resulting bisthioether is stable in blood plasma-mimicking conditions.^9*j*^ Whilst this approach, as well as others, offer advances in this growing field of disulfide labelling, a common limitation is the requirement for reduction and re-bridging in distinct steps. This is mainly due to the incompatibility of the bridging and reducing agents. This introduces inefficiencies in terms of cost, time and practicality. Whilst one-pot *in situ* methods have been employed, this is only at the expense of using a vast excess of reducing and bridging agents to compensate for the reaction between the two reagents.^9*j*^

In view of the above, we set about designing a reagent that could incorporate both reducing and re-bridging functions (Fig. 1). During the course of our previous studies, we observed dithiophenolpyridazinediones to be unreactive towards commonly used disulfide reducing agent tris(2-carboxyethyl)phosphine (TCEP). In light of this, TCEP moieties were a logical choice for incorporation into a dithiophenolpyridazinedione. More specifically, the TCEP functional moieties were to be tethered onto the thiophenol groups of the dithiophenolpyridazinedione since these groups would be extruded post-bioconjugation with a reduced disulfide.

**Fig. 1:**
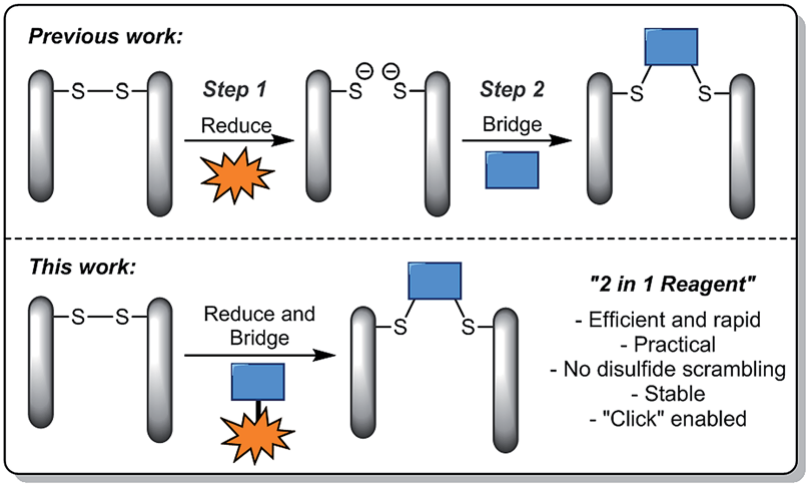
Illustration highlighting previous strategies towards disulfide stapling and the novel strategy described in this manuscript.

A suitable route to a dithioaryl(TCEP)pyridazinedione was conceived (Scheme 1). Initially, TFA cleavage of the Boc groups of di-Boc-diethylhydrazine **1**, followed by reaction with dibromomaleic anhydride under reflux in AcOH, afforded diethyl dibromopyridazinedione **2**. This dibromopyridazinedione was reacted with 4-aminothiophenol to form dithioarylpyridazinedione **3**, which was finally coupled to mono-acid TCEP derivative **4** to form target dithioaryl(TCEP)pyridazinedione **5**.

**Scheme 1:**
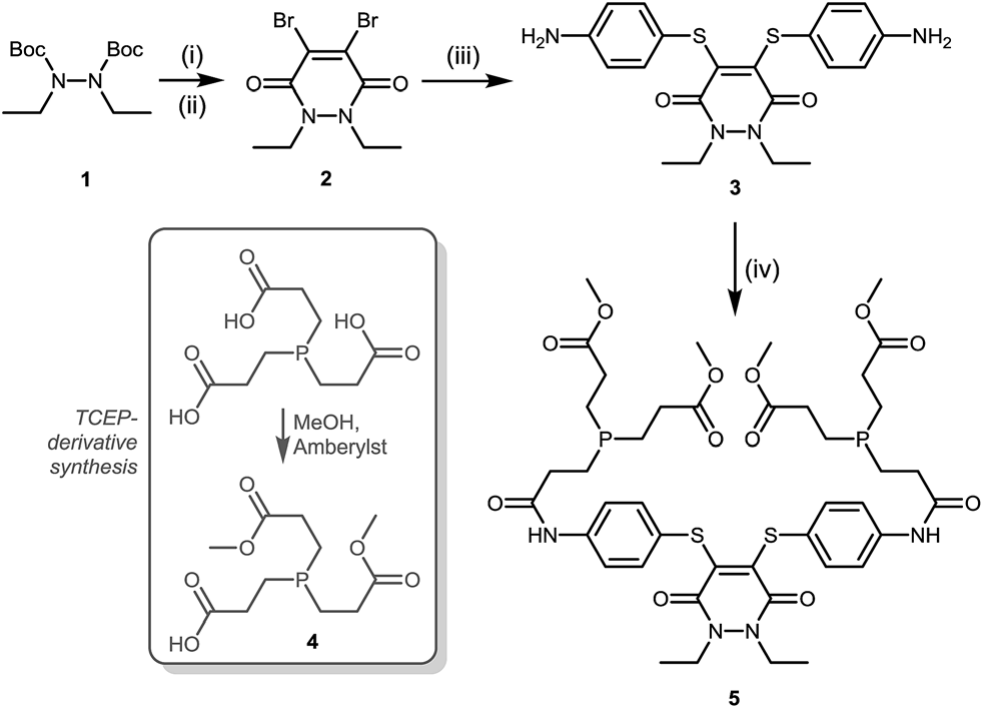
Synthesis of dithioaryl(TCEP)pyridazinedione **5**. Reagents and conditions: (i) TFA, CH_2_Cl_2_, 21 °C, 1 h; (ii) dibromomaleic anhydride, AcOH, reflux, 2 h; (iii) 4-aminothiophenol, NEt_3_, CH_2_Cl_2_, 21 °C, 2 h; (iv) acid **4**, DMF, HATU, DIPEA, 21 °C, 12 h.

With dithioaryl(TCEP)pyridazinedione **5** in hand, we appraised its suitability as a bioconjugation reagent for both disulfide reduction and functional re-bridging. To do this, pyridazinedione **5** was incubated with a selection of biologically relevant disulfide containing peptides and proteins, *i.e.* somatostatin, octreotide and a Fab (fragment antigen-binding) arm of Herceptin™. To our delight, in each and every case, pyridazinedione **5** was shown to reduce and functionally re-bridge the singly accessible disulfide (see Scheme 2 and ESI for further details†). Moreover, only a small excess of “2-in-1” reagent **5**, 1.25 equivalents, was required to effect complete conversion. Another favourable property of pyridazinedione **5** is that it is a solid which can be stored with complete stability over a protracted period at –18 °C under argon.

**Scheme 2:**
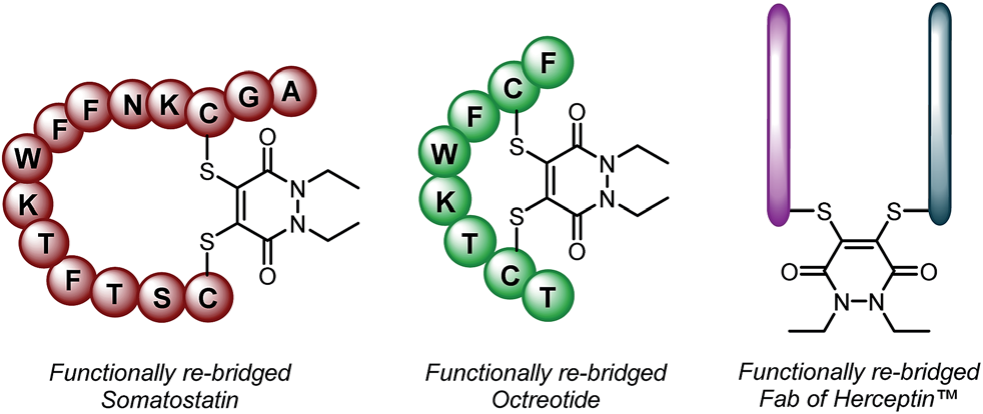
Functionally re-bridged somatostatin, octreotide and a Fab of Herceptin™ using dithioaryl(TCEP)pyridazinedione **5**.

At this stage we rationalised that a molecule with both reducing and re-bridging functions would minimise the residency time of the cysteines liberated from disulfide reduction. This is especially in view of no reduced protein being observed upon incubation of the above peptides and proteins with reagent **5** (see ESI for details†). To appraise this further, we incubated the Fab fragment of Herceptin™ with 1, 2 and 5 equivalents of dipropyl-pyridazinedione **6** prior to incubation with 2 equivalents of diethylpyridazinedione **5** (Scheme 3). Validating our hypothesis, re-bridging was only observed with the TCEP-bearing diethylpyridazinedione. The control reaction of reducing the Fab prior to adding a mixture of pyridazinedione **5** and **6** afforded a mixture of bioconjugates (see ESI for details†).

**Scheme 3:**
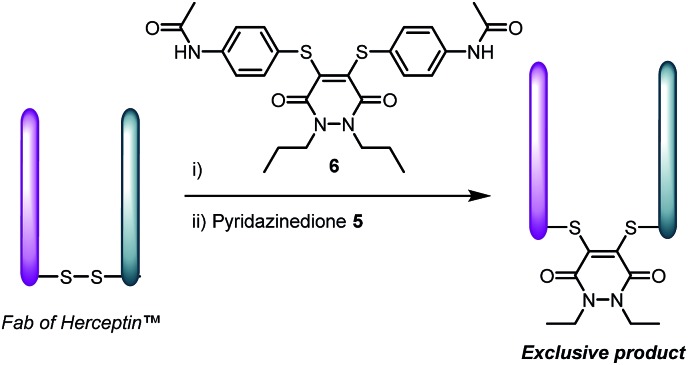
Pre-incubation of Fab fragment of Herceptin™ with pyridazinedione **6**, followed by addition of **5** affords product derived from reagent **5** only.

With the knowledge of the use of dithioaryl(TCEP)pyridazinedione **5** resulting in a high local concentration of the specific pyridazinedione incorporated into the “2-in-1” scaffold, we appraised the use of the reagent in the context of a multi-disulfide system. To do this, we chose to use Herceptin™ – an antibody comprising four disulfide bonds – whose disulfide bonds can be scrambled on attempted functional disulfide re-bridging.^9*h*^ Although this scrambling can be minimised, the leading strategy is reagent specific.^9*j*^ We rationalised that the use of pyridazinedione **5** would ensure minimisation of disulfide scrambling in a general sense. To this end, pyridazinediones **3** (with no internal reducing agent function) and **5** (with reducing agent function) were reacted with Herceptin™ under the appropriate reaction conditions, *i.e.* reaction with pyridazinedione **3** required reduction of Herceptin™ with TCEP. Gratifyingly, no disulfide scrambling was observed by SDS-PAGE for reagent **5**, with complete re-bridging of all disulfides confirmed by UV-Vis (Fig. 2, lane 4). Analogous reagent **3**, with no inherent reducing capability, afforded a disulfide scrambled product (Fig. 2, lane 2). Even when dithiopyridazinedione **3** was used in excess and TCEP was added in small portions multiple times (6 × 0.33 eq per disulfide every 30 min), *i.e.* to minimise the number of open disulfides at any given time, disulfide scrambling was still observed (Fig. 2, lane 3). Although scrambling was far less pronounced, the reaction protocol is highly cumbersome and inefficient. Reaction of dithiopyridazinedione **3** at 37 °C also afforded a mixture of products (Fig. 2, lane 5). Furthermore, reaction of pyridazinedione **2** at 4 °C and 37 °C also afforded a mixture of correctly and incorrectly re-bridged modified Herceptin™ conjugates (see ESI for details†).

**Fig. 2:**
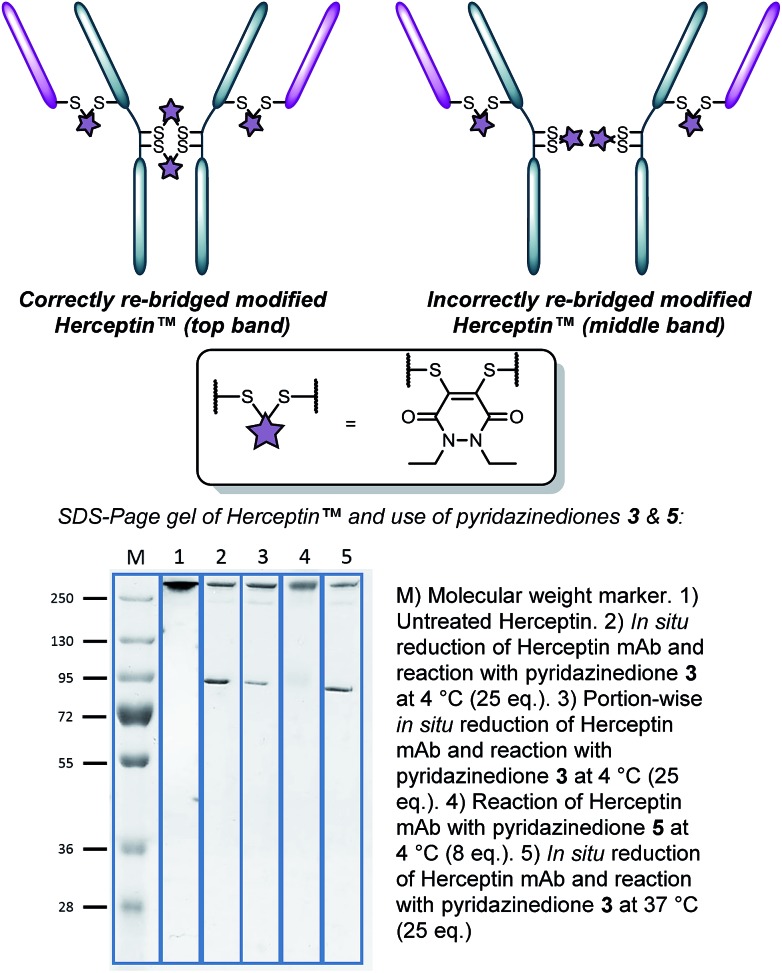
Appraisal of the use of pyridazinediones **3** *(in situ* 4 °C (pre-reduced, portion-wise reduction), 37 °C) and **5** for functional re-bridging of Herceptin™.

Providing a reagent that can functionally re-bridge the disulfide bonds of Herceptin™ without disulfide scrambling is a major contribution in view of the desire to create homogenous conjugates in the field of antibody–drug conjugates.^14^ Use of Herceptin™, in view of its clinical validation alone and as the antibody component of FDA-approved ADC Kadcyla™,^15^ provides direct applicability of the chemistry to this exciting area of targeted therapy.

To make this approach modular and expand its scope, we synthesised an analogue of pyridazinedione **5**, which contained an alkyne handle for “click” functionalisation, in alkyne-pyridazinedione **7** (see Scheme 4). An analogous route to that described in Scheme 1 was followed (see ESI for further details†). The appraisal of this molecule was carried on the Fab fragment of Herceptin™ as this would allow extensive analysis by UV-Vis, MS, SDS-PAGE and ELISA (for binding). The optimised conditions for the insertion of dithioaryl(TCEP)pyridazinedione **5**, was applied to the alkyne bearing analogue for the functional re-bridging of the Fab fragment of Herceptin™ to form conjugate **8**. The efficiency of reduction and re-bridging was translated cleanly from reagent **5** to alkyne analogue **7** by MS, SDS-PAGE and UV-Vis (see Scheme 4 and ESI for further details†).

**Scheme 4:**
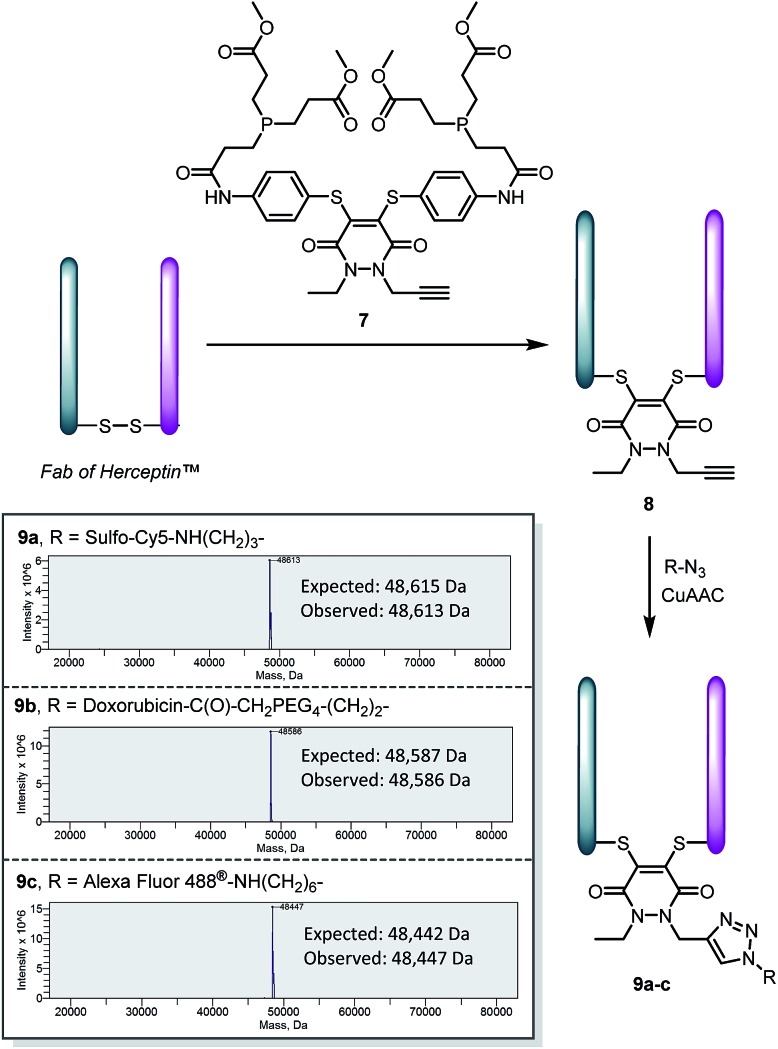
Application of alkyne bearing pyridazinedione **7** for disulfide reduction and re-bridging as well as “click” functionalisation of the product with various azides to form bioconjugates **9a–c**.

Finally, conjugate **8** was functionalised by “click” modification using doxorubicin, AlexaFluor488™ and sulfo-cyanine5 azides (see Scheme 4). In all cases, complete conversion was observed to afford functionalised conjugates **9a–c**, thus demonstrating how the platform may be used for efficient introduction of functional modalities as well as reduction and re-bridging. Moreover, the binding of the Fab protein was not compromised, by ELISA analysis (see ESI for details†), through the chemistry applied. Pyridazinedione **7** was also shown to successfully re-bridge the full antibody system of Herceptin™ and be amenable to “click” functionalisation with doxorubicin azide (see ESI for details†).

## Conclusions

In conclusion, we have provided an important step towards delivering on next-generation disulfide stapling. This first-in class technology allows for reduction and functional re-bridging by the use of a single reagent. Moreover, this strategy has been shown to result in a high local concentration of bridging agent, which has been exploited for the functional re-bridging of a multi-disulfide system (*i.e.* Herceptin™) without disulfide scrambling. Finally, facile “click” functionalisation and retention of binding affinity, using our strategy, has been demonstrated on a Fab of Herceptin™.
