# Supplementary material for: Next-generation disulfide stapling: reduction and functional re-bridging all in one
Source: Chem Sci. 2015 Sep 15;7(1):799–802. doi: 10.1039/c5sc02666k (PMC5580075; doi:10.1039/c5sc02666k)
Supplement: Supplementary file 1 [file SC-007-C5SC02666K-s001.pdf]

Electronic Supplementary Material (ESI) for Chemical Science.
This journal is © The Royal Society of Chemistry 2015

Next-generation disulfide stapling: Reduction and functional re-bridging all in one

Maximillian T. W. Lee, Antoine Maruani, James R. Baker, Stephen Caddick and Vijay Chudasama*

Department of Chemistry, University College London, 20 Gordon Street, London, WC1H OAJ, UK

** Tel: +44 (0)20 76792077; E-mail: v.chudasama@ucl.ac.uk*

General Experimental

All reagents were purchased from Aldrich, AlfaAesar or Lumiprobe and were used as received. Where described below Pet. refers to petroleum ether (40–60 °C). All reactions were monitored by thin-layer chromatography (TLC) on pre-coated SIL G/UV254 silica gel plates (254 μm) purchased from VWR. Flash column chromatography was carried out with Kiesegel 60M 0.04/0.063 mm (200–400 mesh) silica gel. ^{1}H and ^{13}C NMR spectra were recorded at ambient temperature on a Bruker Avance 300 instrument operating at a frequency of 300 MHz for ^{1}H and 75 MHz for ^{13}C, a Bruker Avance 500 instrument operating at a frequency of 500 MHz for ^{1}H and 125 MHz for ^{13}C, and a Bruker Avance 600 instrument operating at a frequency of 600 MHz for ^{1}H and 150 MHz for ^{13}C in $CDCl_3$ or CD_3OD (as indicated below). The chemical shifts (δ) for ^{1}H and ^{13}C are quoted relative to residual signals of the solvent on the ppm scale. ^{1}H NMR peaks are reported as singlet (s), doublet (d), triplet (t), quartet (q), m (multiplet), br (broad) and doublet of quartets (dq). Coupling constants (*J* values) are reported in Hertz (Hz) and are H-H coupling constants unless otherwise stated. Signal multiplicities in ^{13}C NMR were determined using the distortionless enhancement by phase transfer (DEPT) spectral editing technique. Infrared spectra were obtained on a Perkin Elmer Spectrum 100 FTIR Spectrometer operating in ATR mode with frequencies given in reciprocal centimetres (cm^{-1}). Melting points were measured with a Gallenkamp apparatus and are uncorrected. All reagents containing phosphine were stored at -18 °C under argon. All bioconjugation reactions were carried out in duplicate.

Protein LC-MS for Figures S14-S24, S32-S36

LC-MS was performed on protein samples using a Thermo Scientific uPLC connected to MSQ Plus Single Quad Detector (SQD). Column: Hypersil Gold C4, 1.9 μm, 2.1 × 50 mm. Wavelength: 254 nm. Mobile Phase: 99:1 Water (0.1% formic acid): MeCN (0.1% formic acid) to 1:9 Water (0.1% formic acid): MeCN (0.1% formic acid) gradient over 4.5 min. Flow Rate: 0.3 mL/min. MS Mode: ES+. Scan Range: m/z = 500–2000. Scan time: 1.5 s. Data obtained in continuum mode. The electrospray source of the MS was operated with a capillary voltage of 3.5 kV and a cone voltage of 50 V. Nitrogen was used as the nebulizer and desolvation gas at a total flow of 600 L/h. Ion series were generated by integration of the total ion chromatogram (TIC) over the 3.0–5.0 min range. Total mass spectra for protein samples were reconstructed from the ion series using the pre-installed ProMass software using default settings for large proteins in m/z range 500–1500.

Peptide MALDI-TOF MS for Figure S25

Samples were prepared by diluting with matrix (α-cyano-4-hydroxy-cinnamic acid in water-acetonitrile (2:8, v/v)), 0.5% formic acid. 3 μL of sample was spotted onto the MALDI target plate and allowed to dry. Samples were analysed using a Waters MALDI micro MX (Waters, UK) with a nitrogen laser in positive

reflectron mode using source TLF delay (500 ns), an accelerating voltage of 120 V, pulse 2500 and detector 2000. Acquisition was between 500–30000 Da with 100 shots/spectrum.

UV-vis spectroscopy

UV-vis spectroscopy was used to determine protein concentrations and pyridazinedione to antibody ratios (PAR) using a nanodrop ND-1000 spectrophotometer (Figure **S26**–**29**) and a Varian Cary 100 Bio UV-Visible spectrophotometer (Figure **S30**, **S31**, **S41** and **S42**), operating at room temperature. Sample buffer was used as blank for baseline correction.

SDS-PAGE gels

Non-reducing glycine-SDS-PAGE at 12% acrylamide gels were performed following standard lab procedures. A 6% stacking gel was used and a broad-range MW marker (10–250 kDa, Prestained PagerulerPlus Protein Standards, Bio-Rad) was co-run to estimate protein weights. Samples (15 μL at ~12 μM construct) were mixed with loading buffer (3 μL, composition for 6 × SDS: 1 g SDS, 3 mL glycerol, 6 mL 0.5 M Tris buffer pH 6.8, 2 mg bromophenol blue in 10 mL) and heated at 75 °C for 3 min. The gels were run at 30 mA for 50 min in 1 × SDS running buffer. The gels were stained with Coomassie dye.

Synthesis of compounds

Di-*tert*-butyl 1,2-diethylhydrazine-1,2-dicarboxylate (1)

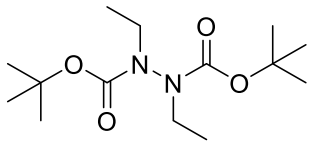

To a solution of di-*tert*-butyl hydrazine-1,2-dicarboxylate (1.0 g, 4.3 mmol) and caesium carbonate (5.6 g, 17.2 mmol) in DMF (20 mL) was added bromoethane (1.1 g, 10.1 mmol) and the reaction mixture stirred at 21 °C for 24 h. After this time, the reaction mixture was diluted with EtOAc (50 mL) and washed with deionised water (4 × 20 mL) and brine (2 × 20 mL). The organic phase was then concentrated *in vacuo* and the crude residue purified by flash column chromatography (50% EtOAc:Pet.). The appropriate fractions were combined and concentrated *in vacuo* to afford di-*tert*-butyl 1,2-diethylhydrazine-1,2-dicarboxylate (1.1 g, 3.8 mmol, 88%) as a colourless oil as a mixture of rotamers. ^{1}H NMR (300 MHz, $CDCl_3$) δ 3.57–3.33 (m, 4H), 1.50–1.39 (m, 18H), 1.16 (t, J = 7.2 Hz, 6H); ^{13}C NMR (75 MHz, $CDCl_3$) δ 155.1 (C), 80.6 (C), 46.37 (CH_2), 44.37 (CH_2), 28.35 (CH_3), 28.27 (CH_3), 13.56 (CH_3), 12.95 (CH_3); IR (thin film) 2980, 2927, 2881, 1698 cm^{-1}; LRMS (ES+) 288 (100, $[M+H]^+$); HRMS (ES+) calcd for $C_{16}H_{32}N_2O$ $[M+H]^+$ 288.2049, observed 288.2054.

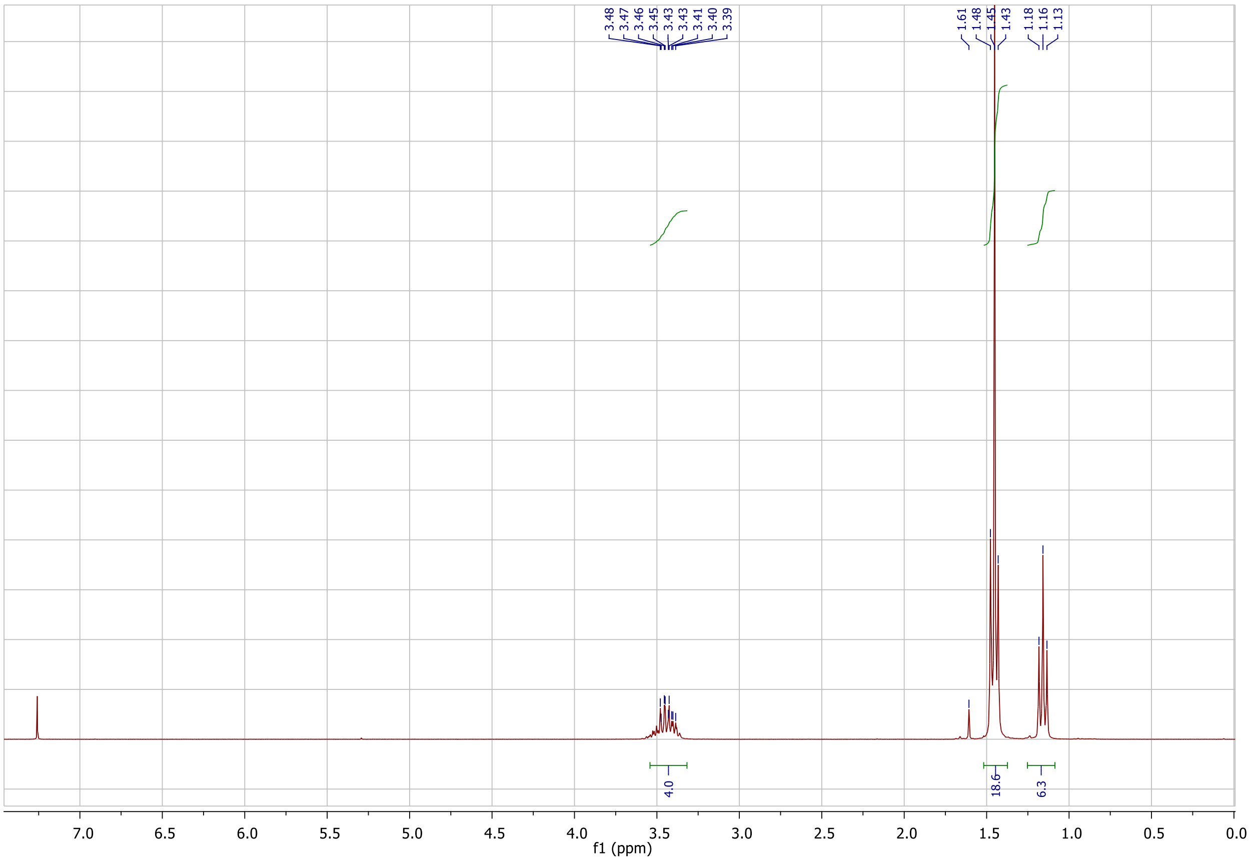

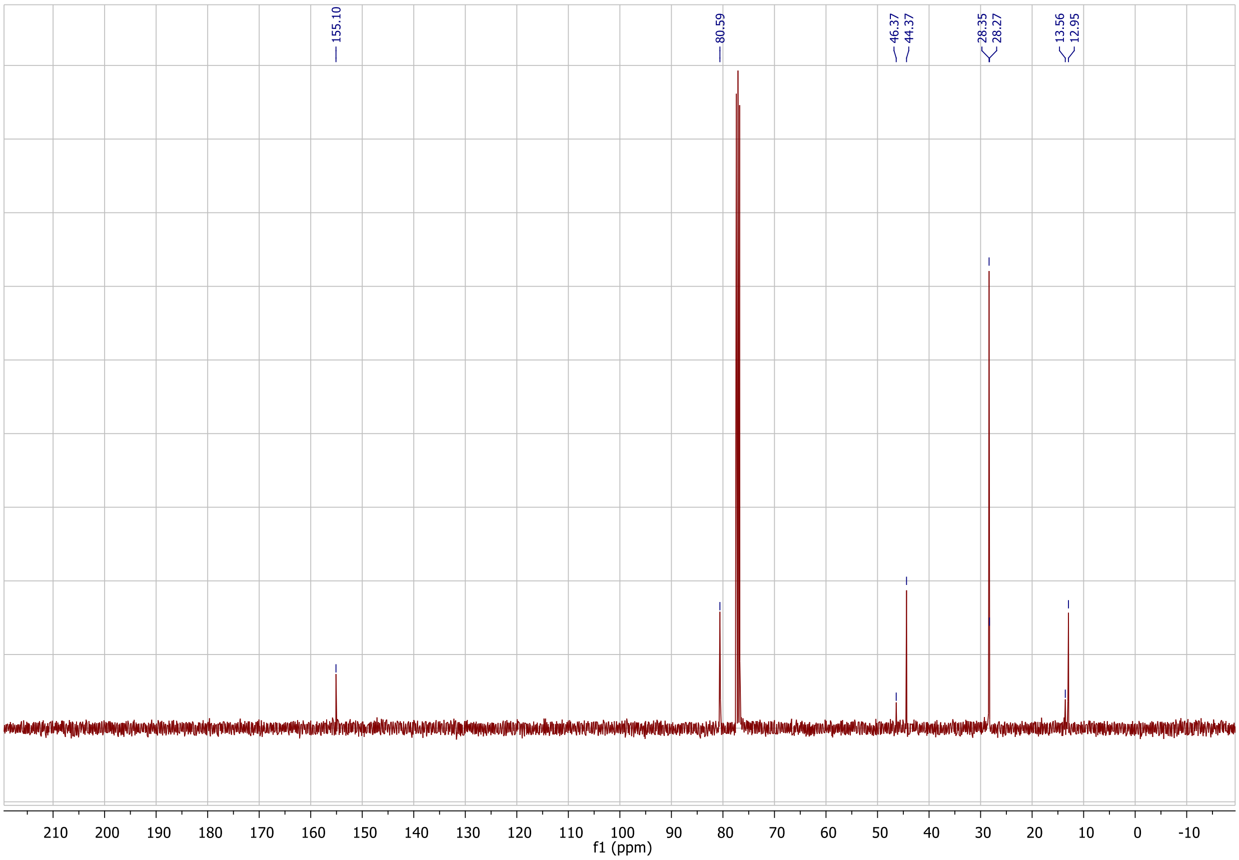

Figure **S1**. ^{1}H and ^{13}C NMR data for di-*tert*-butyl 1,2-diethylhydrazine-1,2-dicarboxylate (**1**).

4,5-Dibromo-1,2-diethyl-1,2-dihydropyridazine-3,6-dione (Pyridazinedione 2)[1]

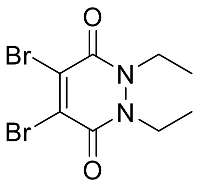

To a 1:1 solution of CH_2Cl_2:TFA (5 mL:5 mL) was added di-*tert*-butyl 1,2-diethylhydrazine-1,2-dicarboxylate **1** (348 mg, 1.21 mmol) and the reaction mixture stirred at 21 °C for 1 h. The reaction mixture was then concentrated *in vacuo*, taking care to ensure all the TFA was removed by use of toluene to form an azeotrope. The crude residue was then dissolved in AcOH (10 mL), to which was added 3,4-dibromomaleic anhydride (310 mg, 1.21 mmol) and the reaction mixture heated under reflux for 2 h. The reaction mixture was then concentrated *in vacuo* and the crude residue was purified by flash column chromatography (30–50 % EtOAc:Pet.). The appropriate fractions were then combined and concentrated *in vacuo* to afford 4,5-dibromo-1,2-diethyl-1,2-dihydropyridazine-3,6-dione (288 mg, 0.88 mmol, 73%) as a pale yellow solid. ^{1}H NMR (600 MHz, $CDCl_3$) δ 4.17 (q, *J* = 7.0 Hz, 4H), 1.28 (t, *J* = 7.0 Hz, 6H); ^{13}C NMR (150 MHz, $CDCl_3$) δ 153.3 (C), 136.1 (C), 42.4 (CH_2), 13.2 (CH_3); IR (solid) 2979, 2937, 1630, 1574 cm^{-1}; LRMS (EI) 328 (50, [M^{81}Br^{81}Br]$^+$), 326 (100, [M^{81}Br^{79}Br]$^+$), 324 (50, [M^{79}Br^{79}Br]$^+$); HRMS (EI) calcd for $C_8H_{10}Br_2N_2O_2$ [M^{79}Br^{79}Br]$^+$ 323.9104, observed 323.9097.

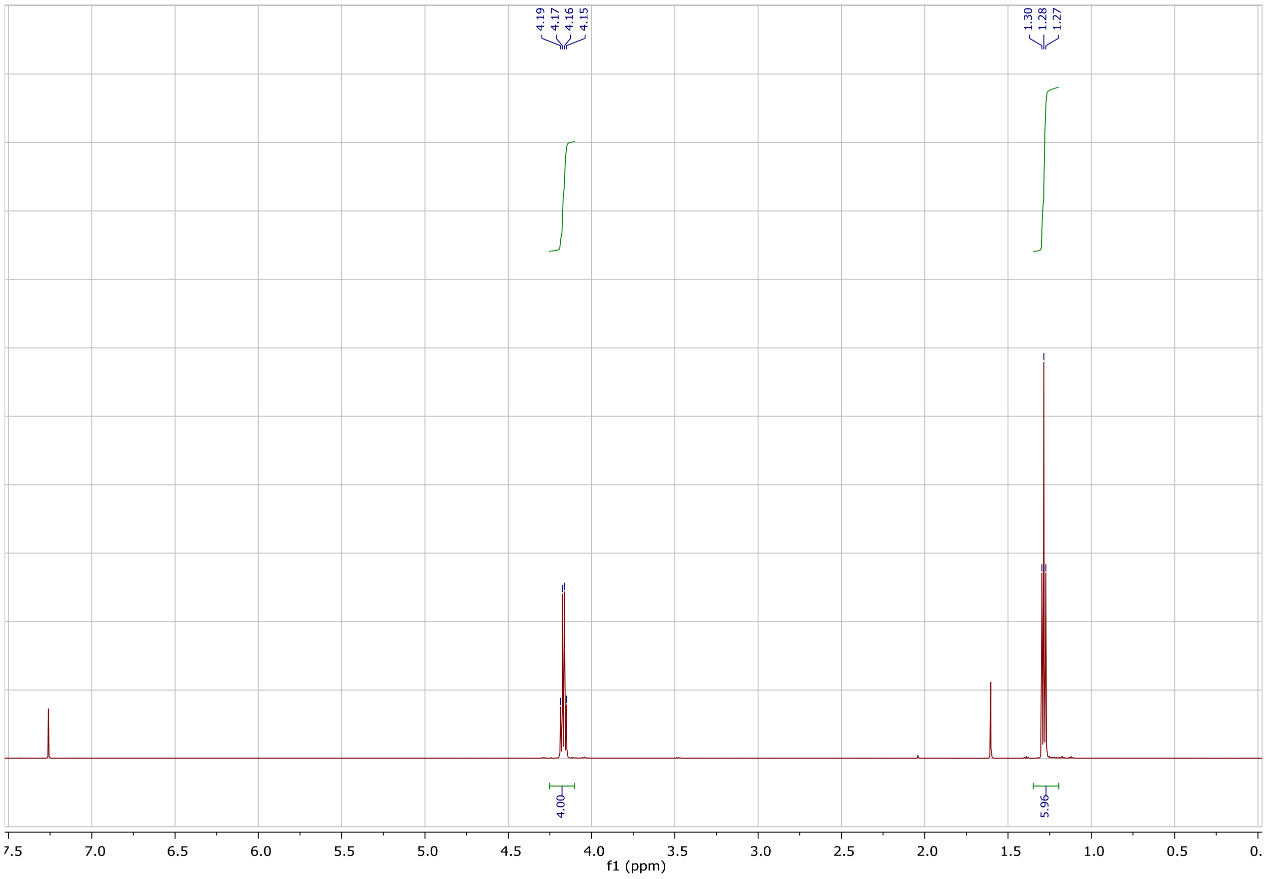

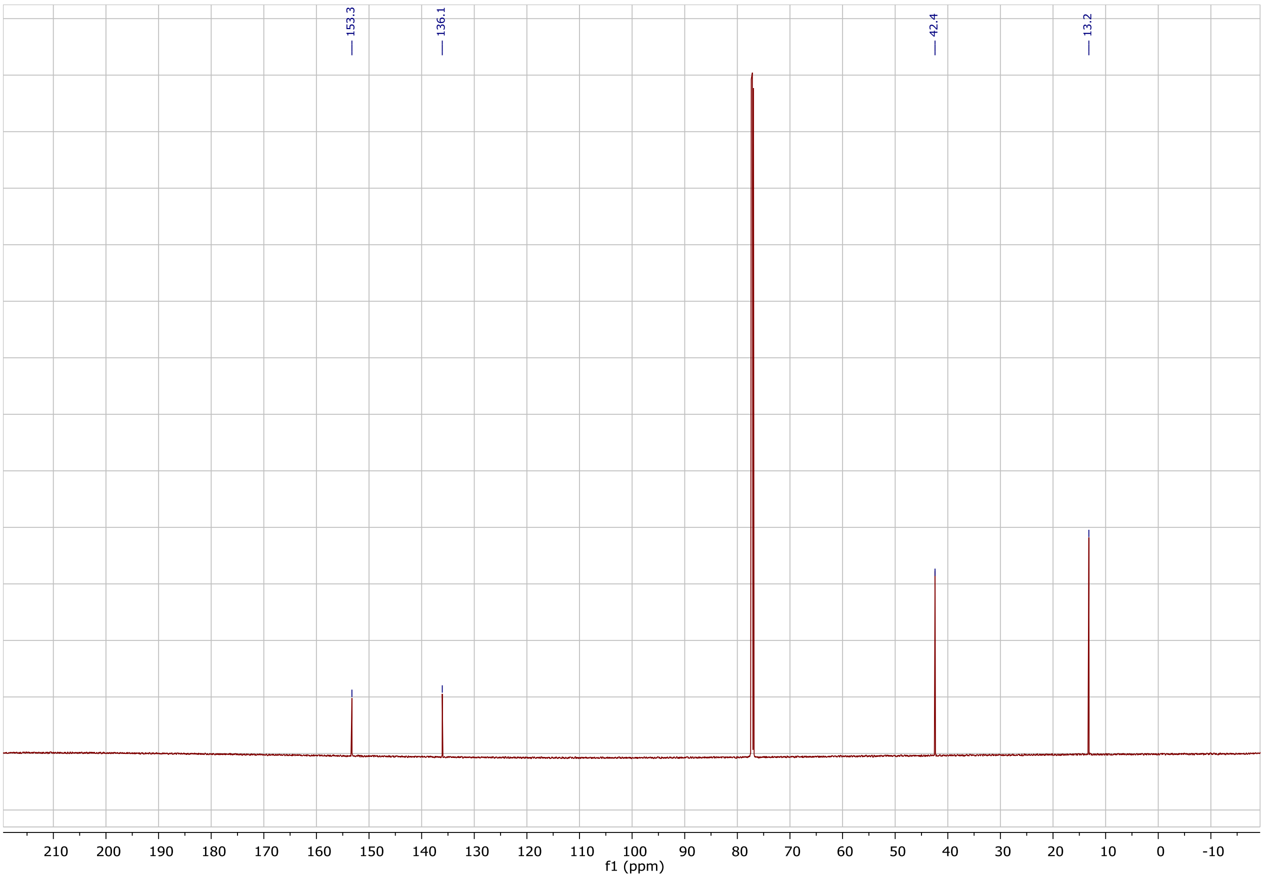

Figure **S2**. ^{1}H and ^{13}C NMR data for 4,5-dibromo-1,2-diethyl-1,2-dihydropyridazine-3,6-dione (Pyridazinedione **2**).

4,5-Bis((4-aminophenyl)thio)-1,2-diethyl-1,2-dihydropyridazine-3,6-dione (Pyridazinedione 3)

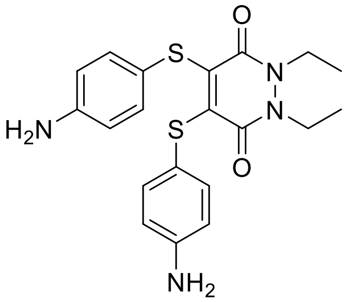

To a solution of 4,5-dibromo-1,2-diethyl-1,2-dihydropyridazine-3,6-dione **2** (193 mg, 0.60 mmol) and 4-aminothiophenol (89 mg, 0.71 mmol) in CH_2Cl_2 (20 ml) was added NEt_3 (166 µL, 1.20 mmol) and the reaction mixture stirred at 21 °C for 2 h. The reaction mixture was then washed with deionised water (3 × 15 mL), the organic phase was concentrated *in vacuo* and the crude residue purified by flash column chromatography (40–60% EtOAc:Pet.). The appropriate fractions were combined and concentrated *in vacuo* to give 4,5-bis((4-aminophenyl)thio)-1,2-diethyl-1,2-dihydropyridazine-3,6-dione (163 mg, 0.39 mmol, 66%) as an orange-red crystalline solid. m.p. 158–162 °C; ^{1}H NMR (300 MHz, $CDCl_3$) δ 7.18–7.08 (m, 4H), 6.62–6.52 (m, 4H), 3.98 (q, *J* = 7.0 Hz, 4H), 3.75 (br. s, 4H), 1.17 (t, *J* = 7.0 Hz, 4H); ^{13}C NMR (75 MHz, $CDCl_3$) δ 156.4 (C), 146.8 (C), 142.3 (C), 134.1 (C), 120.4 (CH), 115.6 (CH), 41.0 (CH_2), 12.7 (CH_3); IR (solid) 3420, 3247, 2975, 1708, 1653, 1596, 1495, 1397 cm^{-1}; LRMS (ES+) 415 (100, $[M+H]^+$); HRMS (ES+) calcd for $C_{20}H_{22}N_4O_2S_2$ $[M+H]^+$ 415.1262, observed 415.1272.

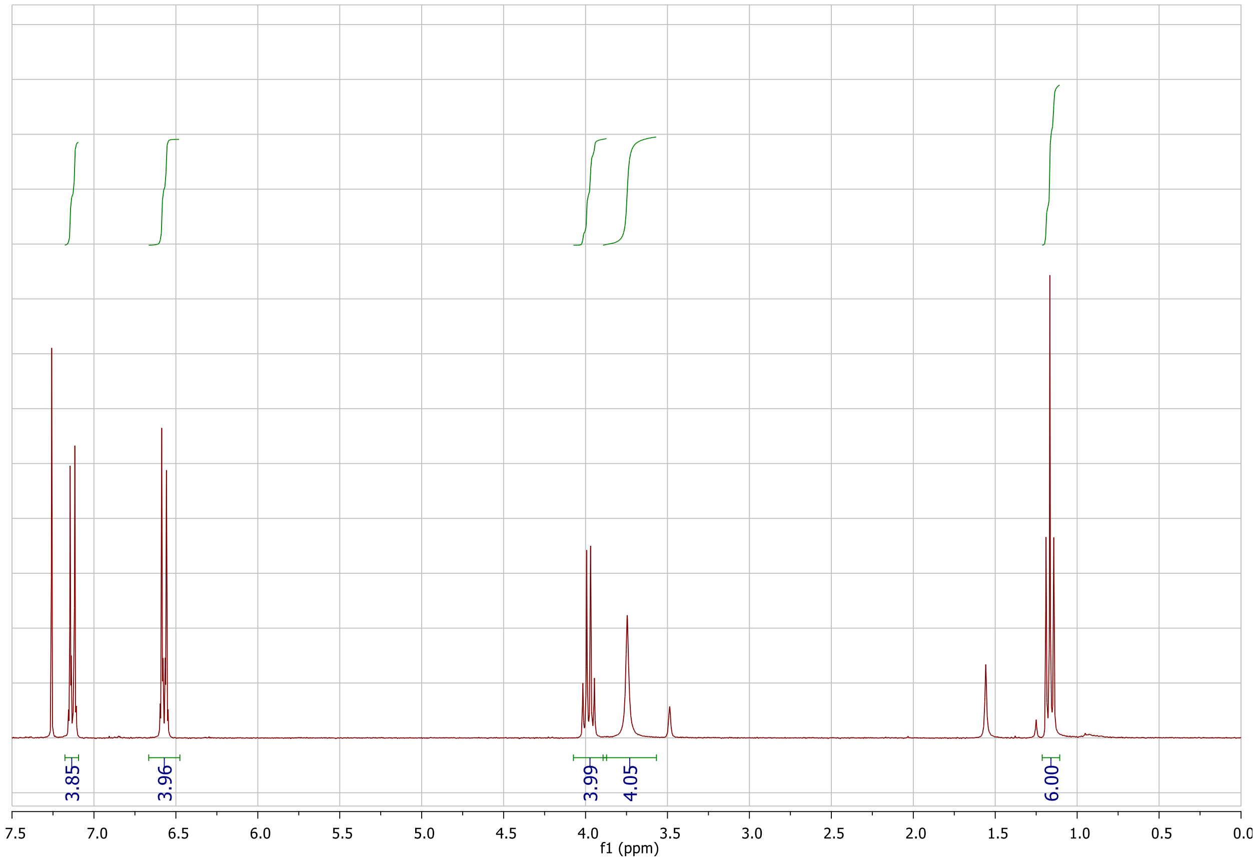

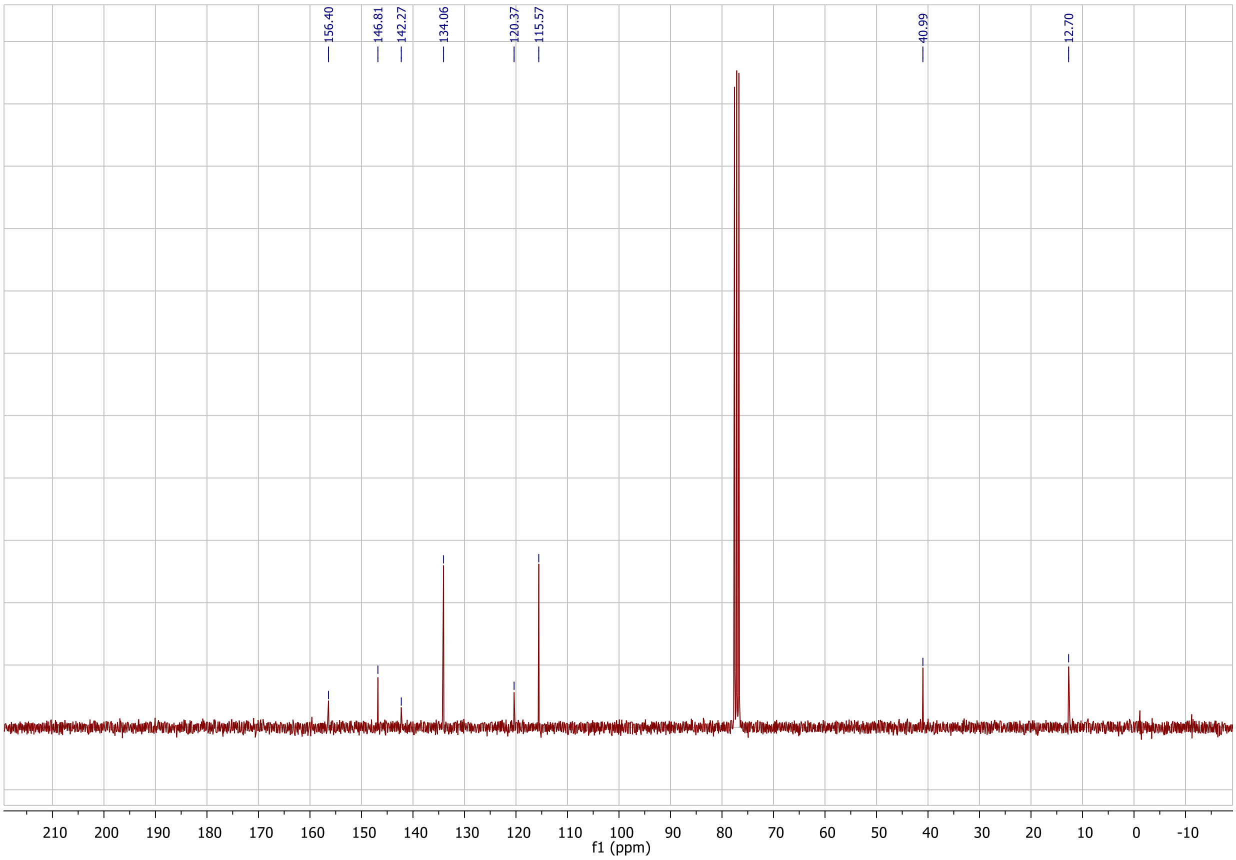

Figure **S3**. ^{1}H and ^{13}C NMR data for 4,5-bis((4-aminophenyl)thio)-1,2-diethyl-1,2-dihydropyridazine-3,6-dione (Pyridazinedione **3**).

3-(Bis(3-methoxy-3-oxopropyl)phosphanyl)propanoic acid (4)[2]

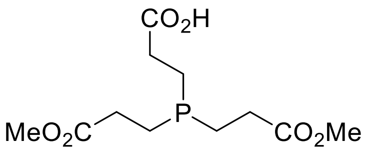

To a solution of tris(2-carboxyethyl)phosphine hydrochloride (1.0 g, 3.5 mmol) in MeOH (15 mL), was added Amberlyst® 15 beads (1.0 g), which had been freshly pre-washed with MeOH (10 mL) for 10 mins, filtered and then dried. The reaction mixture was stirred at 21 °C for 1 h. The reaction mixture was then filtered to remove the Amberlyst® beads, the filtrate was concentrated *in vacuo* and the crude residue purified by flash column chromatography (1–2% MeOH:CH_2Cl_2). The appropriate fractions were collected and concentrated *in vacuo* to give 3-(bis(3-methoxy-3-oxopropyl)phosphanyl)propanoic acid (170 mg, 0.61 mmol, 17%) as a clear oil. ^{1}H NMR (600 MHz, $CDCl_3$) δ 3.70 (s, 6H), 2.62–2.36 (m, 6H), 1.76 (t, J = 7.9 Hz, 6H); ^{13}C NMR (150 MHz, $CDCl_3$) δ 173.8 (C), 173.7 (C), 52.0 (CH_3), 30.7 (CH_2), 30.5 (CH_2), 21.5 (CH_2) (d, J_{C-P} = 13.1 Hz), 21.3 (CH_2) (d, J_{C-P} = 13.2 Hz); LRMS (ES-) 277 (100, $[M-H]^-$); HRMS (ES-) calcd for $C_{11}H_{19}O_6P$ $[M-H]^-$ 277.0841, observed 277.0838.

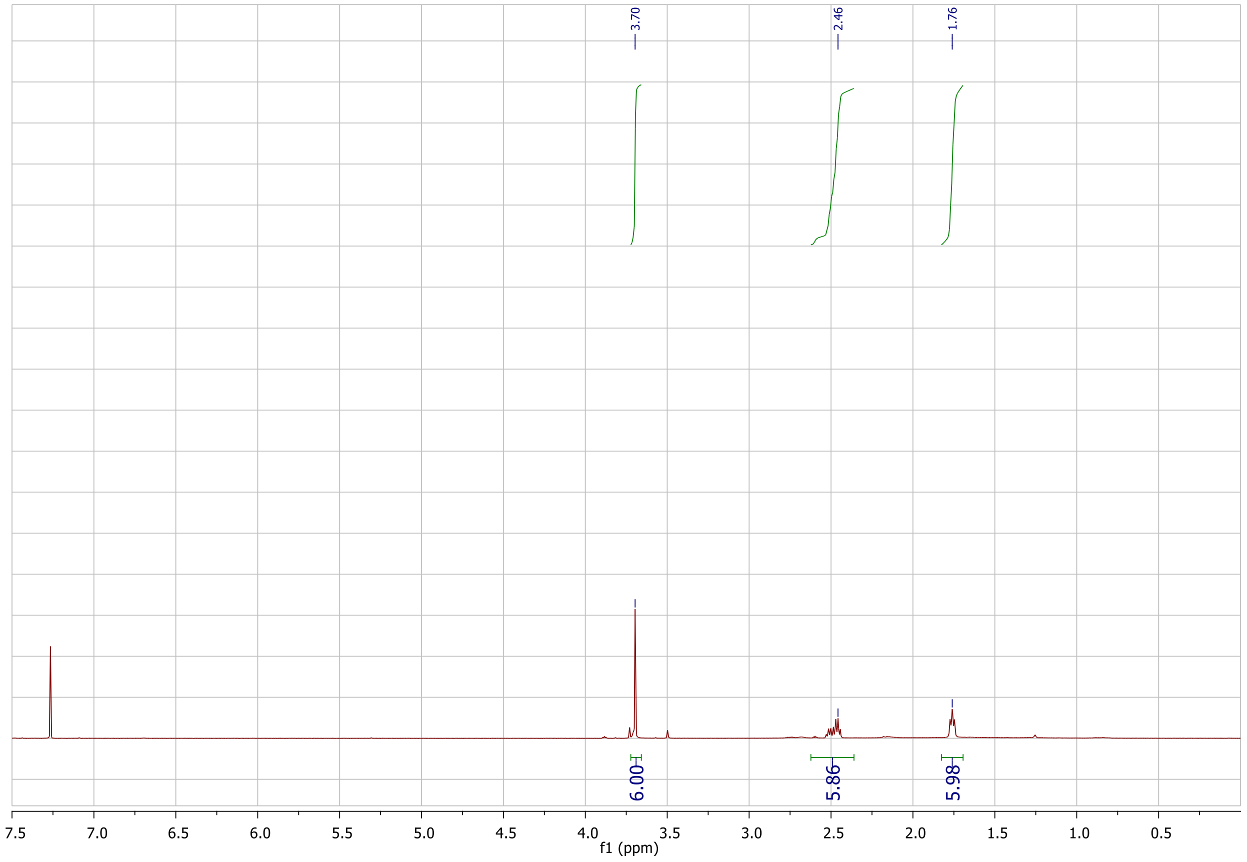

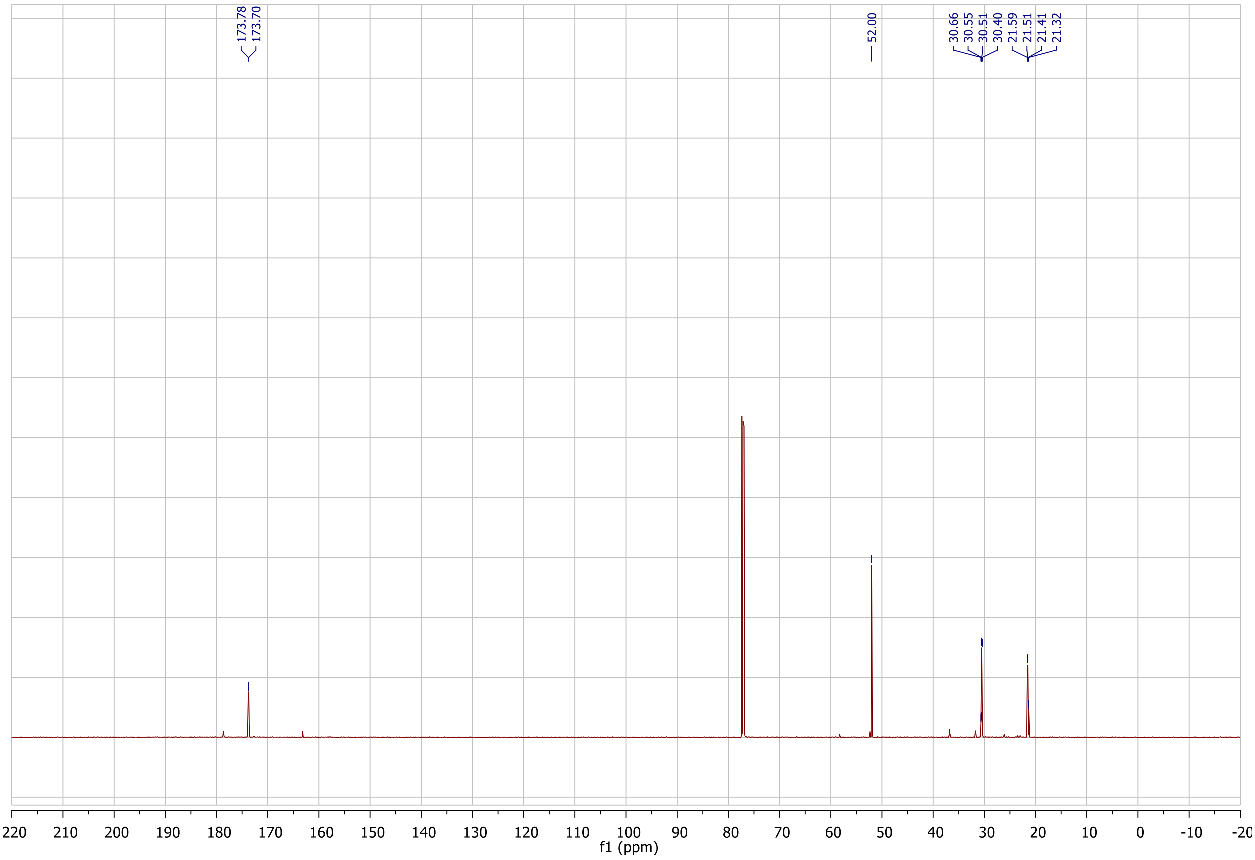

Figure **S4**. ^{1}H and ^{13}C NMR data for 3-(bis(3-methoxy-3-oxopropyl)phosphanyl)propanoic acid (**4**).

Tetramethyl 3,3',3'',3'''-((((((1,2-diethyl-3,6-dioxo-1,2,3,6-tetrahydropyridazine-4,5-diyl)bis(sulfanediyl))bis(4,1-phenylene))bis(azanediyl))bis(3-oxopropane-3,1-diyl))bis(phosphanetriyl))tetrapropionate (Pyridazinedione 5)

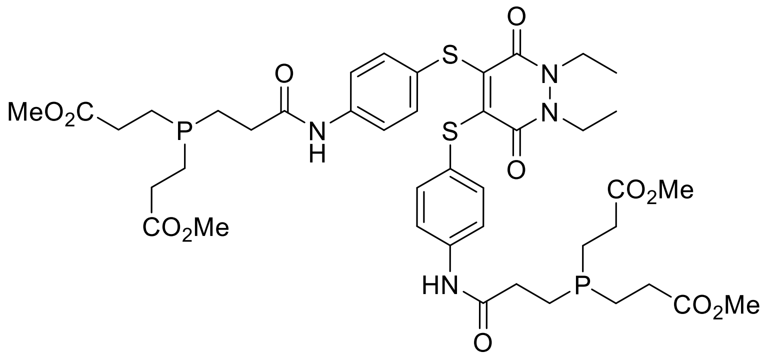

To a solution of **4** (31 mg, 0.11 mmol) in DMF (3 ml) was added HATU (42 mg, 0.11 mmol) and the reaction stirred at 21 °C for 15 mins. Following this, 4,5-bis((4-aminophenyl)thio)-1,2-diethyl-1,2-dihydropyridazine-3,6-dione **3** (12 mg, 0.03 mmol) was added and the reaction mixture stirred for a further 5 mins. After this, *N*,*N*-diisopropylethylamine (DIPEA) (20 µL, 0.11 mmol) was added and the reaction mixture stirred at 21 °C for 12 h. Following this, the reaction mixture was diluted with EtOAc (20 mL), and washed with deionised water (3 × 10 mL) and brine (3 × 10 mL). The organic phase was then concentrated *in vacuo* and the crude residue purified by flash column chromatography (0–2% MeOH:CH_2Cl_2). The appropriate fractions were then combined and concentrated *in vacuo* to give pyridazinedione **5** (6 mg, 0.01 mmol, 18%) as a yellow gum. ^{1}H NMR (600 MHz, $CDCl_3$) δ 7.80 (s, 2H), 7.45 (d, *J* = 8.4 Hz, 4H), 7.16 (d, *J* = 8.3 Hz, 4H), 4.04 (q, *J* = 7.0 Hz, 4H), 3.69 (s, 12H), 2.50 (s, 12H), 1.91–1.67 (m, 12H), 1.22 (t, *J* = 7.0 Hz, 6H); ^{13}C NMR (150 MHz, $CDCl_3$) δ 174.0 (C), 173.9 (C), 170.9 (C), 156.0 (C), 138.1 (C), 132.2 (C), 127.1 (C), 120.1 (C), 52.1 (CH_3), 41.2 (CH_2) , 33.9 (CH_2), 30.6 (d, J_{C-P} =16.5, CH_2), 22.3 (d, J_{C-P}=13.5, CH_2), 21.6 (d, J_{C-P}=13.5, CH_2), 12.9 (CH_3); IR (thin film) 3325, 2953, 1725, 1614, 1593, 1527, 1494, 1409, 1367; LRMS (ES+) 951 (100, $[M(O)+H]^+$); HRMS (ES+) calcd for $C_{42}H_{56}O_{13}P_2S_2$ $[M(O)+H]^+$ 951.2839, observed 951.2887.

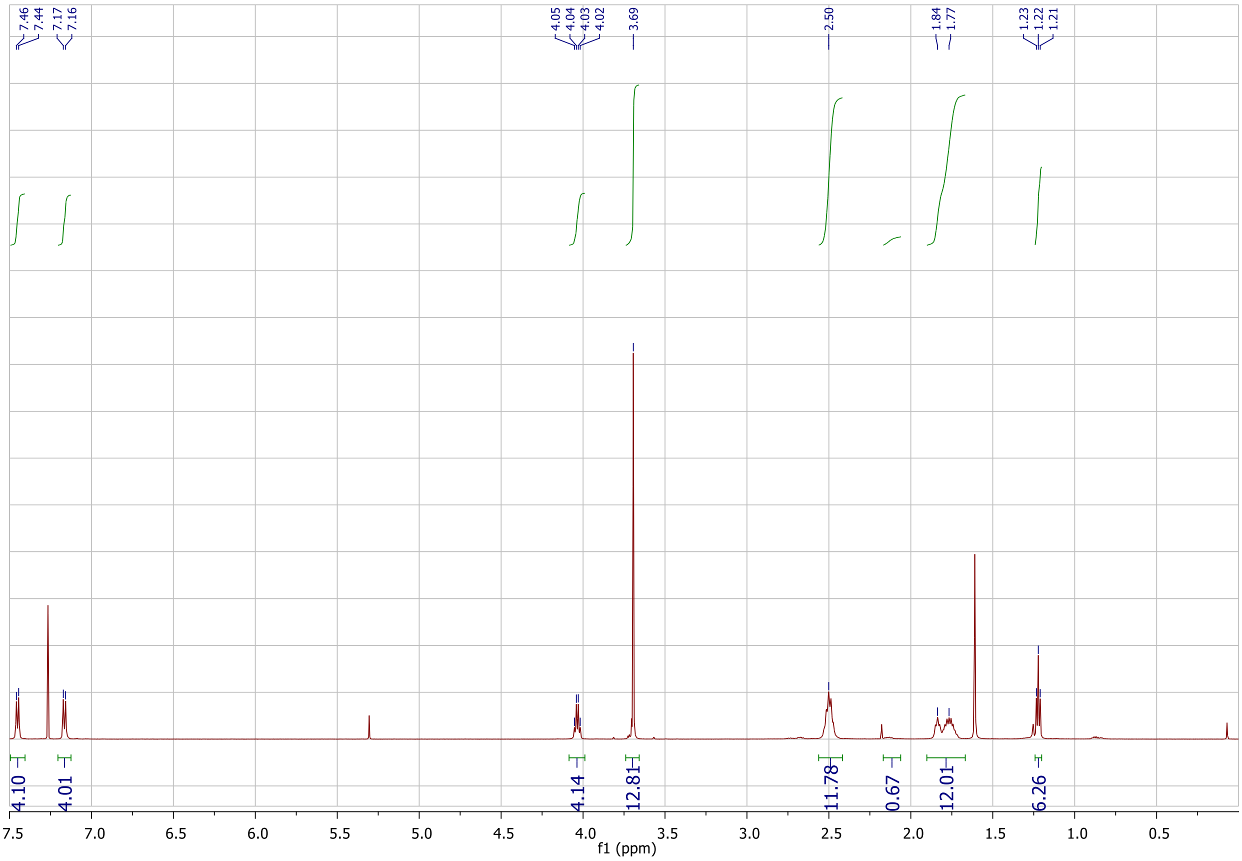

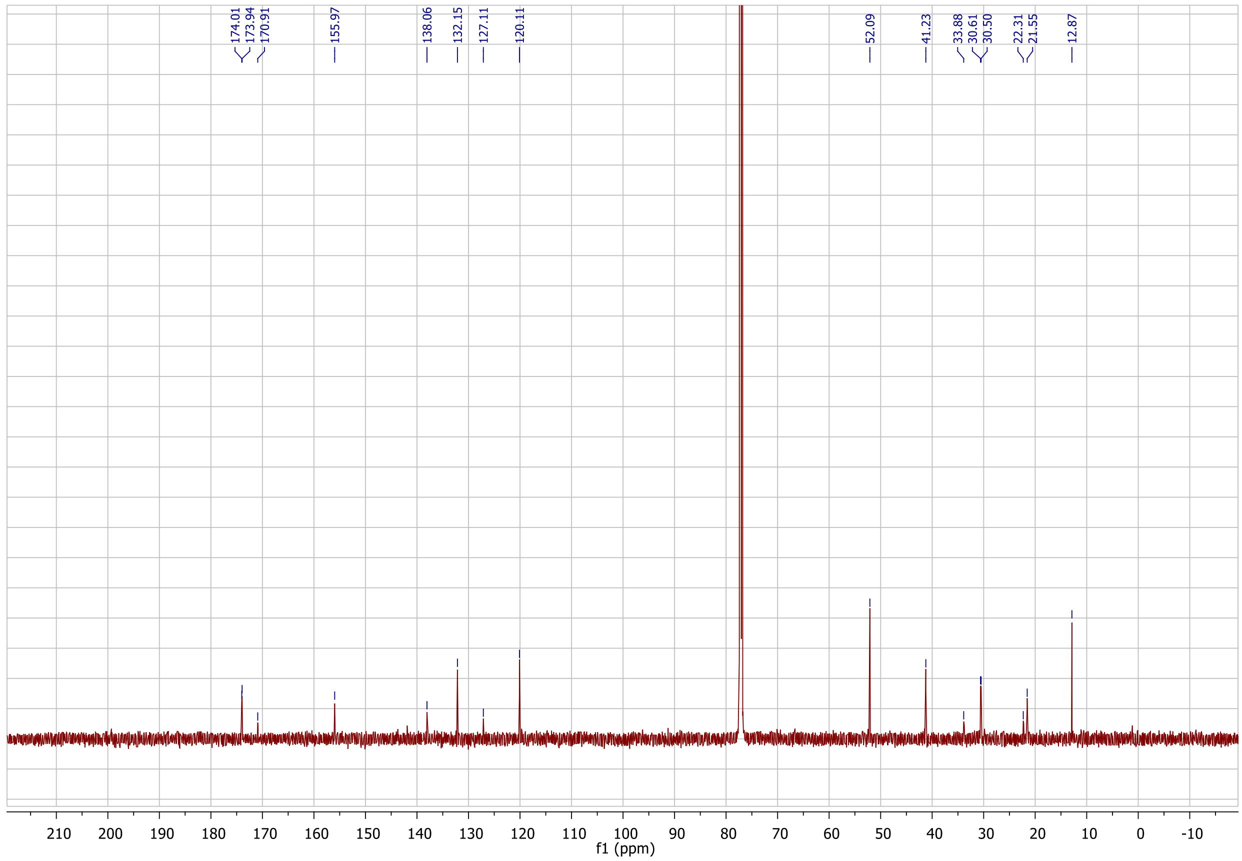

Figure **S5**. ^{1}H and ^{13}C NMR data for tetramethyl 3,3',3'',3'''-((((((1,2-diethyl-3,6-dioxo-1,2,3,6-tetrahydropyridazine-4,5-diyl)bis(sulfanediyl))bis(4,1-phenylene))bis(azanediyl))bis(3-oxopropane-3,1-diyl))bis(phosphanetriyl))tetrapropionate (Pyridazinedione **5**).

Di-*tert*-butyl 1,2-dipropylhydrazine-1,2-dicarboxylate

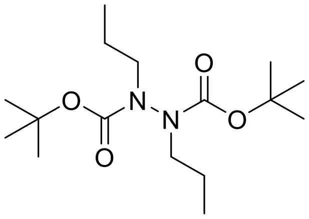

To a solution of di-*tert*-butyl hydrazine-1,2-dicarboxylate (1.0 g, 4.3 mmol) and caesium carbonate (5.6 g, 17.2 mmol) in DMF (20 mL) was added bromopropane (1.1 g, 9.5 mmol) and the reaction mixture stirred at 21 °C for 24 h. After this time, the reaction mixture was diluted with EtOAc (50 mL) and washed with deionised water (4 × 20 mL) and brine (2 × 20 mL). The organic phase was then concentrated *in vacuo* and the crude residue purified by flash column chromatography (50% EtOAc:Pet.). The appropriate fractions were combined and concentrated *in vacuo* to afford di-*tert*-butyl 1,2-dipropylhydrazine-1,2-dicarboxylate (1.1 g, 3.36 mmol, 78%) as a colourless oil as a mixture of rotamers. ^{1}H NMR (600 MHz, $CDCl_3$) δ 3.50–3.17 (m, 4H), 1.61 (s, 4H), 1.51–1.38 (m, 18H), 0.90 (t, J = 7.4 Hz, 6H); ^{13}C NMR (150 MHz, $CDCl_3$) δ 156.0 (C), 155.3 (C), 154.9 (C), 80.9 (C), 80.7 (C), 80.7 (C), 80.6 (C), 53.5 (CH_2), 51.8 (CH_2), 51.7 (CH_2), 28.4 (CH_3), 28.3 (CH_3), 22.0 (CH_3), 21.3 (CH_3), 21.1 (CH_3), 11.7 (CH_3), 11.6 (CH_3), 11.5 (CH_3); IR (thin film) 2974, 2934, 2877, 1707 cm^{-1}; LRMS (ES+) 317 (100, [M+H]$^{+}$); HRMS (ES+) calcd for $C_{16}H_{32}N_2O$ [M+H]$^{+}$ 317.2440, observed 317.2425.

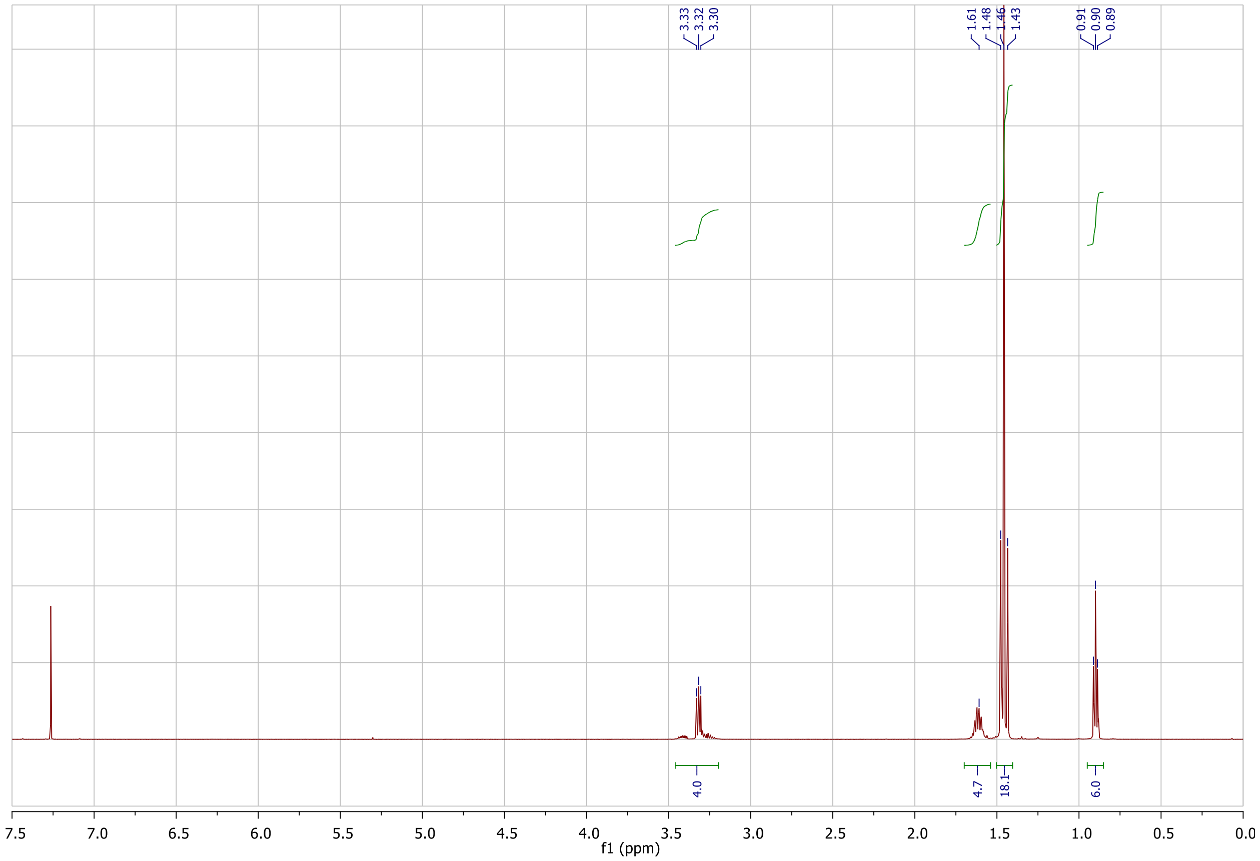

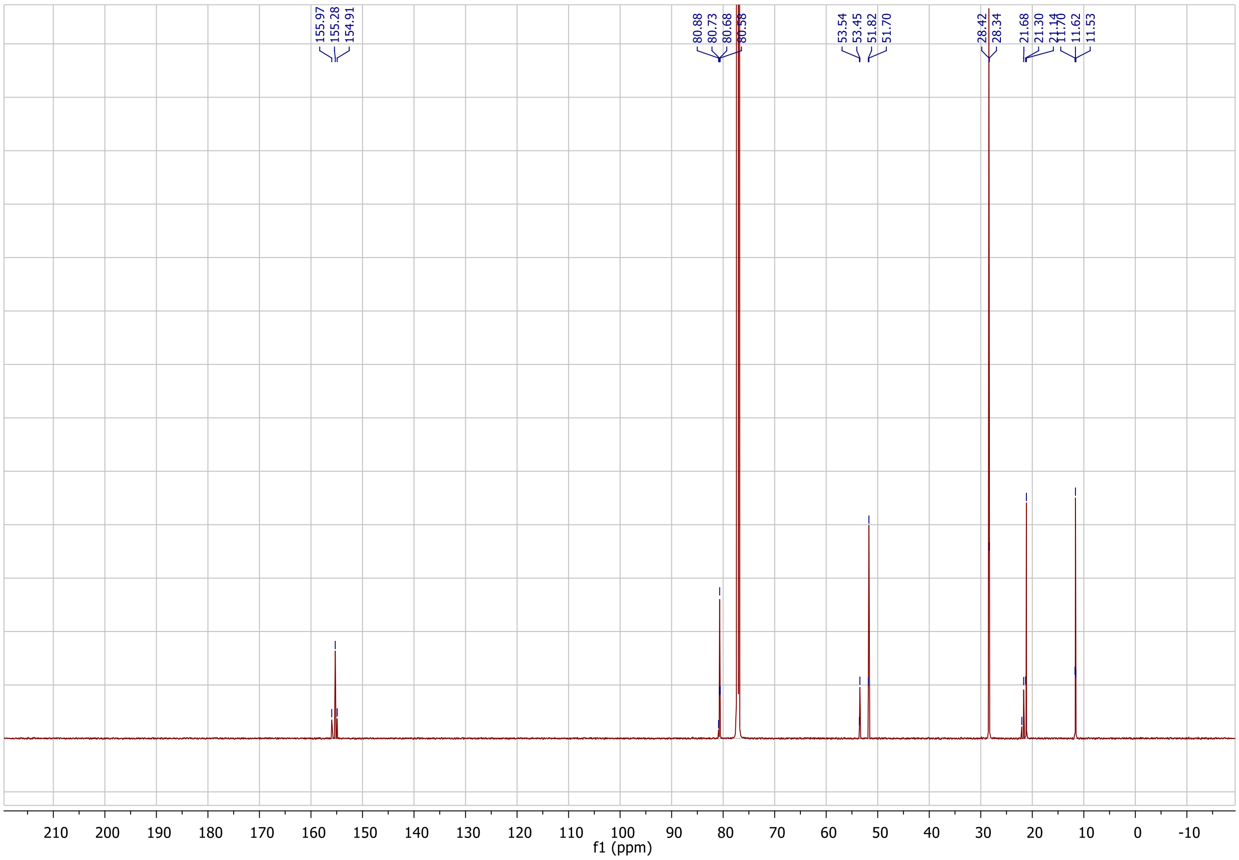

Figure **S6**. ^{1}H and ^{13}C NMR data for di-*tert*-butyl 1,2-dipropylhydrazine-1,2-dicarboxylate.

4,5-Dibromo-1,2-dipropyl-1,2-dihydropyridazine-3,6-dione

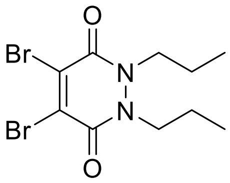

To a 1:1 solution of CH_2Cl_2:TFA (5 mL:5 mL) was added di-*tert*-butyl 1,2-dipropylhydrazine-1,2-dicarboxylate (150 mg, 0.47 mmol) and the reaction mixture stirred at 21 °C for 1 h. The reaction mixture was then concentrated *in vacuo*, taking care to ensure all the TFA was removed by use of toluene as an azeotrope. The crude residue was then dissolved in AcOH (10 mL), to which was added 3,4-dibromomaleic anhydride (121 mg, 0.47 mmol) and the reaction mixture heated under reflux for 2 h. The reaction mixture was then concentrated *in vacuo* and the crude residue was purified by flash column chromatography (30–50 % EtOAc:Pet.). The appropriate fractions were then combined and concentrated *in vacuo* to afford 4,5-dibromo-1,2-dipropyl-1,2-dihydropyridazine-3,6-dione (130 mg, 0.37 mmol, 78%) as a pale yellow gum. ^{1}H NMR (300 MHz, $CDCl_3$) δ 4.04 (t, *J* = 7.2 Hz, 4H), 1.67 (dq, *J* = 7.5 Hz, 4H), 0.93 (t, *J* = 7.5 Hz, 6H); ^{13}C NMR (75 MHz, $CDCl_3$) δ 153.6 (C), 135.9 (C), 48.5 (CH_2), 21.5 (CH_2), 11.0 (CH_3); LRMS (EI) 356 (50, $[M^{81}Br^{81}Br+H]^+$), 354 (100, $[M^{81}Br^{79}Br+H]^+$), 352 (50, $[M^{79}Br^{79}Br+H]^+$); HRMS (EI) calcd for $C_{10}H_{14}Br_2N_2O_2$ $[M^{79}Br^{79}Br]^+$ 351.9422, observed 351.9420.

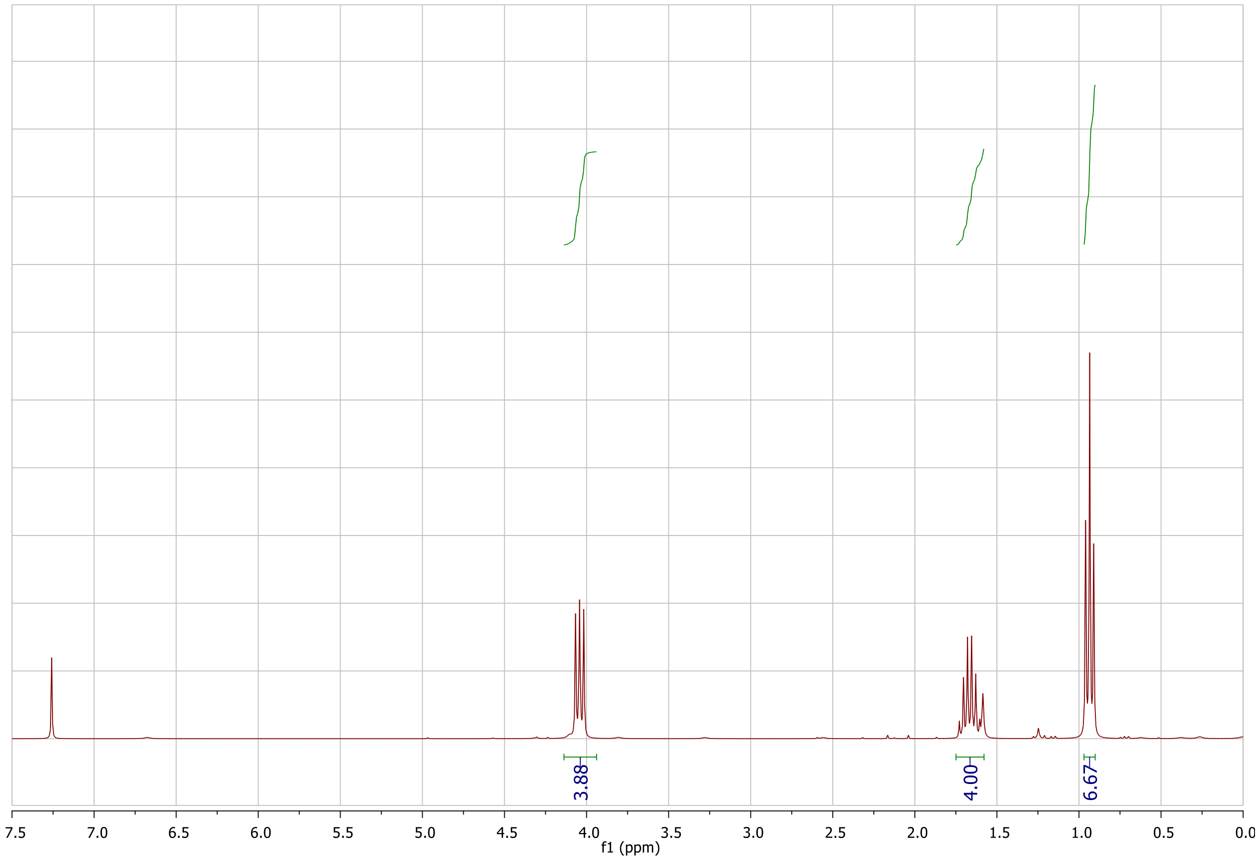

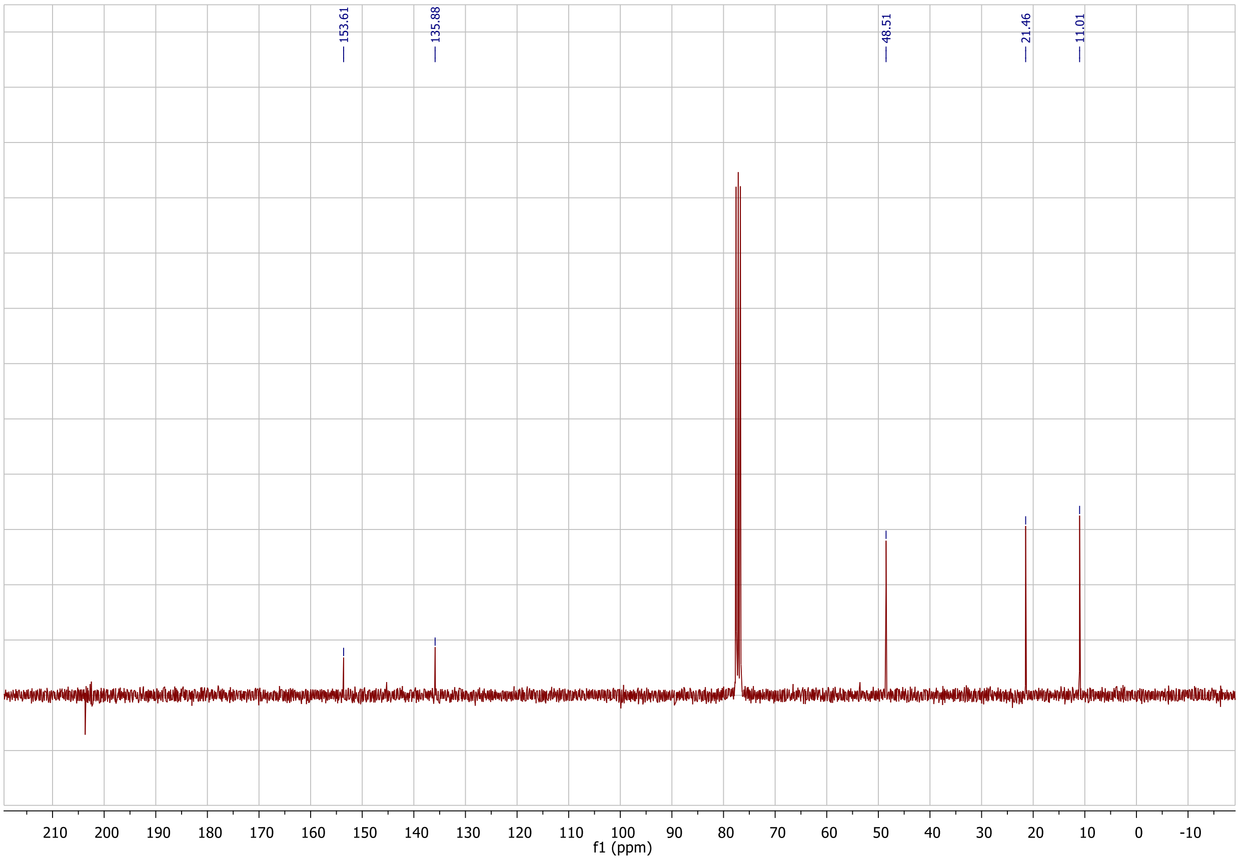

Figure **S7**. ^{1}H and ^{13}C NMR data for 4,5-dibromo-1,2-dipropyl-1,2-dihydropyridazine-3,6-dione.

***N,N'*-(((3,6-Dioxo-1,2-dipropyl-1,2,3,6-tetrahydropyridazine-4,5-diyl)bis(sulfanediyl))bis(4,1-phenylene))diacetamide (Pyridazinedione 6)**

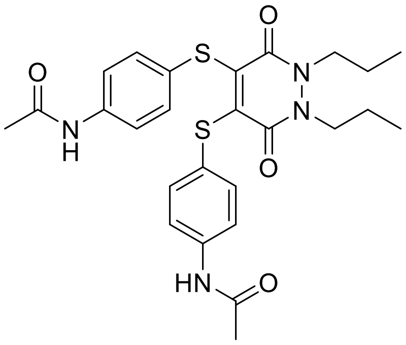

To a solution of 4-acetimidothiophenol (169 mg, 1.01 mmol) and 4,5-dibromo-1,2-dipropyl-1,2-dihydropyridazine-3,6-dione (136 mg, 0.37 mmol) in CH_2Cl_2 (20 mL) was added NEt_3 (212 µL, 1.47 mmol) and the reaction mixture stirred at 21 °C over 72 h. The reaction mixture was then washed with deionised water (3 × 15 mL) and the organic phase concentrated *in vacuo*. The crude residue was then purified by flash column chromatography (2% MeOH:CH_2Cl_2), and the appropriate fractions combined and concentrated *in vacuo* to give *N,N'*-(((3,6-dioxo-1,2-dipropyl-1,2,3,6-tetrahydropyridazine-4,5-diyl)bis(sulfanediyl))bis(4,1-phenylene))diacetamide (78 mg, 0.15 mmol, 40%) as a bright red crystalline solid. m.p. 147–150 °C; ^{1}H NMR (600 MHz, $CDCl_3$) δ 7.40 (d, *J* = 8.2 Hz, 4H), 7.15 (d, *J* = 8.5 Hz, 4H), 3.94 (t, *J* = 7.3 Hz, 4H), 2.18 (s, 4H), 1.62 (dq, *J* = 7.3 Hz, 6H), 0.89 (t, *J* = 7.4 Hz, 6H); ^{13}C NMR (150 MHz, $CDCl_3$) δ 168.5 (C), 156.5 (C), 137.9 (C), 132.1 (C), 127.3 (CH), 120.2 (CH), 120.1 (CH), 47.0 (CH_2), 24.8 (CH_2), 21.1 (CH_2), 11.0 (CH_3); IR (solid) 3314, 2966, 2929, 2876, 1694, 1632, 1592, 1527, 1493 cm^{-1}; LRMS (ES+) 527 (100, $[M+H]^+$); HRMS (ES+) calcd for $C_{26}H_{30}N_4O_4S_2$ $[M+H]^+$ 527.1787, observed 527.1798.

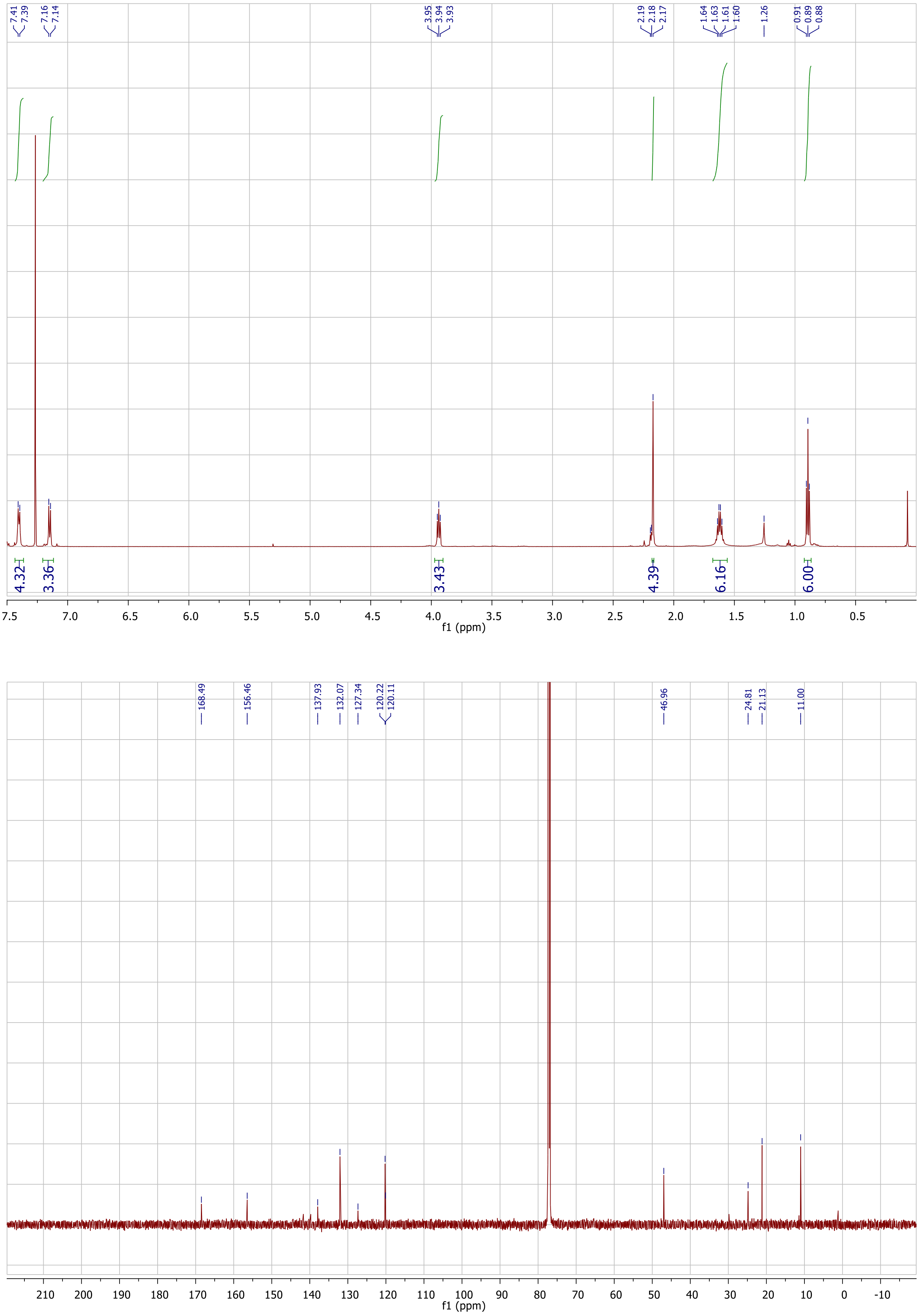

Figure **S8**. ^{1}H and ^{13}C NMR data for *N*,*N'*-(((3,6-dioxo-1,2-dipropyl-1,2,3,6-tetrahydropyridazine-4,5-diyl)bis(sulfanediyl))bis(4,1-phenylene))diacetamide (Pyridazinedione **6**).

Di-*tert*-butyl 1-ethylhydrazine-1,2-dicarboxylate

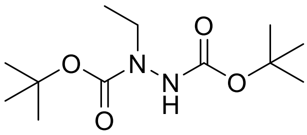

To a solution of di-*tert*-butyl hydrazine-1,2-dicarboxylate (7.0 g, 30.13 mmol) and caesium carbonate (4.0 g, 12.06 mmol) in DMF (50 mL) was added bromoethane (644 µL, 6.03 mmol) and the reaction mixture left to stir at 21 °C for 18 h. After this time, the reaction mixture was diluted with EtOAc (75 mL) and washed with deionised water (3 × 40 mL) and brine (3 × 30 mL). The organic phase was then concentrated *in vacuo*, and cold hexane was added to the crude residue to precipitate out and recover the excess di-*tert*-butyl hydrazine-1,2-dicarboxylate as a white solid through recrystallisation and filtration. The filtrate was then concentrated further *in vacuo* and purified by flash column chromatography (5–15% EtOAc:Pet.). The appropriate fractions were combined and concentrated *in vacuo* to give di-*tert*-butyl 1-ethylhydrazine-1,2-dicarboxylate (642 mg, 2.46 mmol, 41%) as a white crystalline solid (rotamers present in solution NMR). m.p. 54–57 °C ^{1}H NMR (600 MHz, $CDCl_3$) δ 6.15 (br. d, 1H), 3.49 (br. s, 2H), 1.48-1.45 (m, 18H), 1.14 (t, J = 7.2 Hz, 3H); ^{13}C NMR (150 MHz, $CDCl_3$) δ 155.3 (C), 81.2 (C), 81.0 (C), 45.8 (CH_2), 44.1 (CH_2), 28.4 (CH_3), 28.3 (CH_3), 12.7 (CH_3); IR (solid) 3313, 2978, 2934, 1706 cm^{-1}; LRMS (ES+) 261 (100, $[M+H]^+$); HRMS (ES+) calcd for $C_{12}H_{24}N_2O_4$ $[M+H]^+$ 261.1814, observed 261.1813.

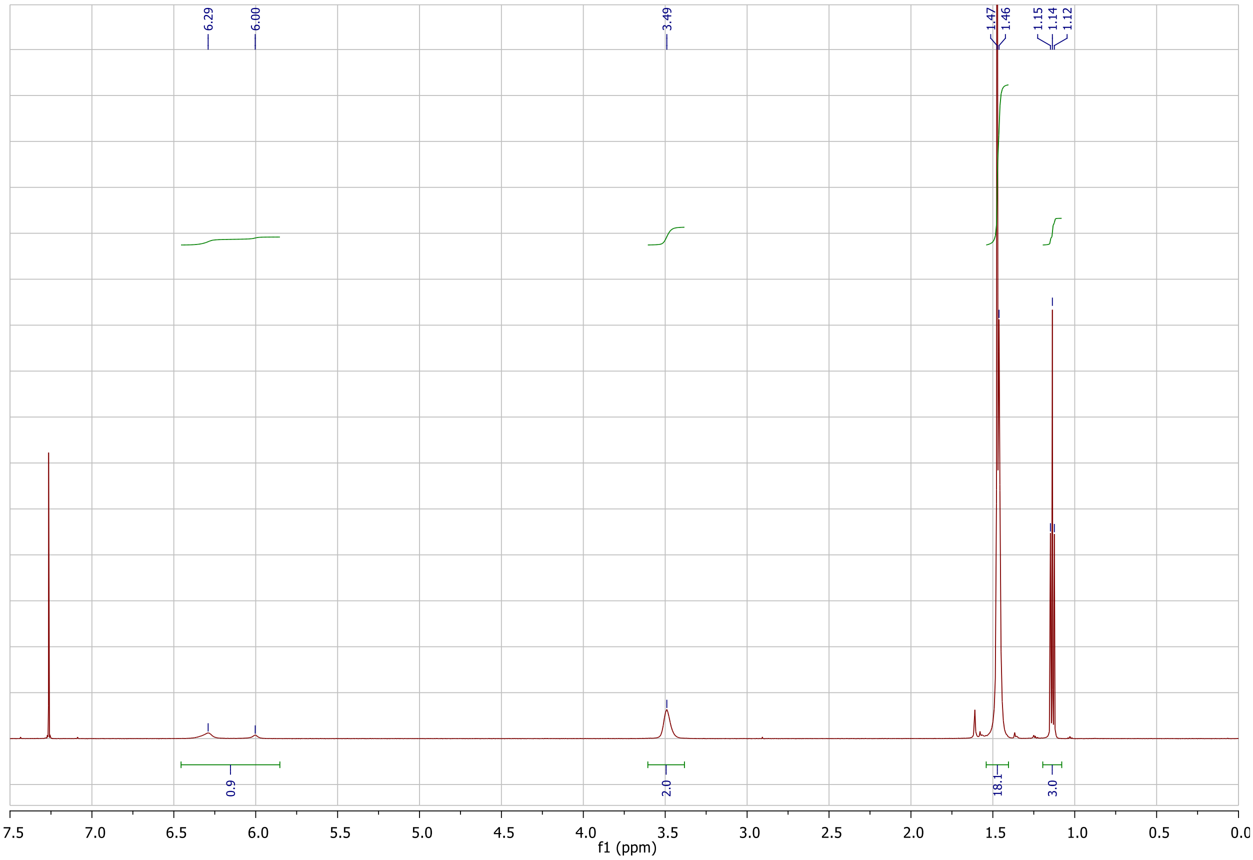

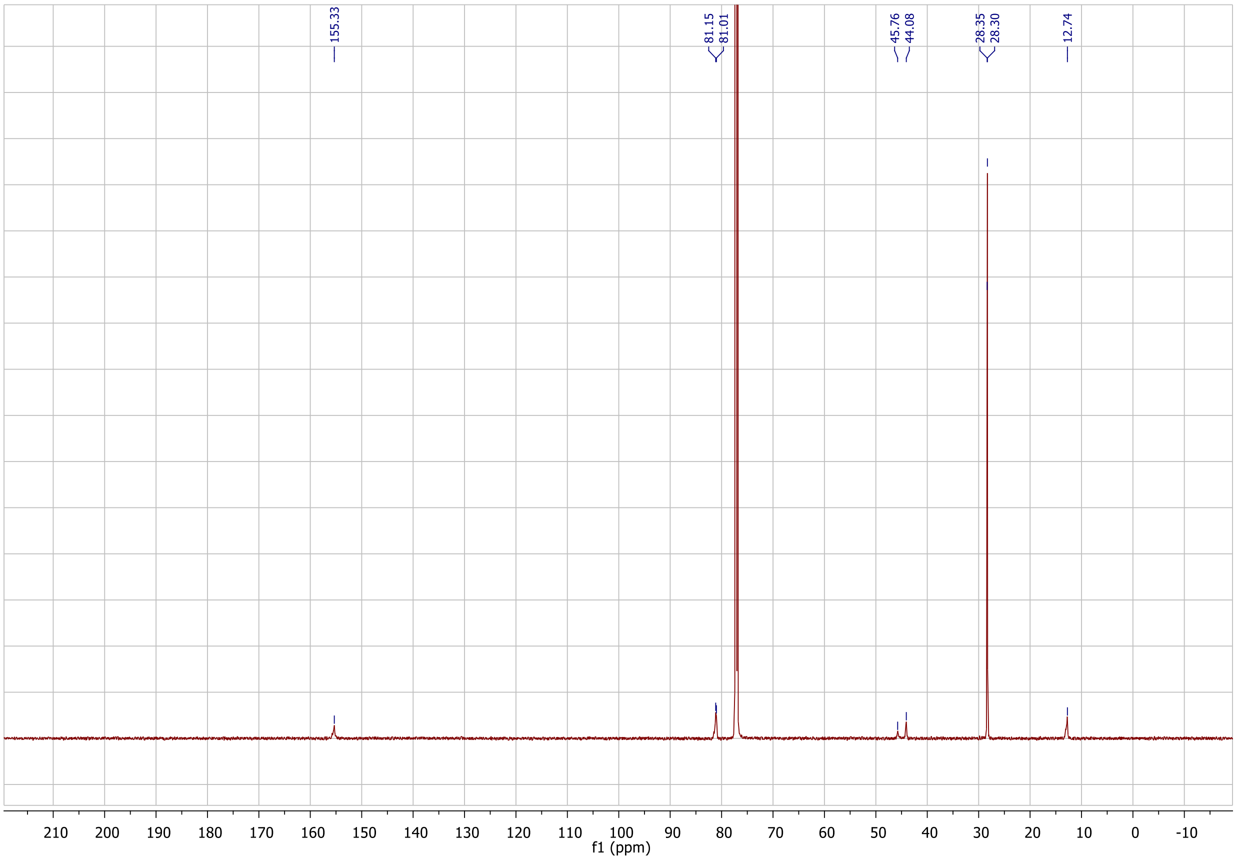

Figure **S9**. ^{1}H and ^{13}C NMR data for di-*tert*-butyl 1-ethylhydrazine-1,2-dicarboxylate.

Di-*tert*-butyl 1-ethyl-2-(prop-2-yn-1-yl)hydrazine-1,2-dicarboxylate

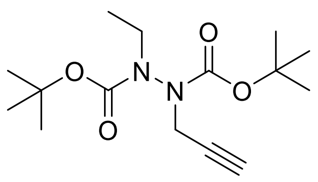

To a solution of di-*tert*-butyl 1-ethylhydrazine-1,2-dicarboxylate (642 mg, 2.47 mmol) and caesium carbonate (2.0 g, 6.14 mmol) in DMF (20 mL) was added propargyl bromide (1 mL, 19.4 mmol) and the reaction mixture stirred at 21 °C for 72 h. The reaction mixture was then diluted with EtOAc (30 mL) and washed with deionised water (3 × 20 mL) and brine (3 × 20 mL). The organic phase was then concentrated *in vacuo* and purified by flash column chromatography (60% EtOAc:Pet.). The appropriate fractions were collected and concentrated *in vacuo* to give di-*tert*-butyl 1-ethyl-2-(prop-2-yn-1-yl)hydrazine-1,2-dicarboxylate (259 mg, 0.87 mmol, 35%) as a colourless oil as a mixture of rotamers. ^{1}H NMR (600 MHz, $CDCl_3$) δ 4.70–4.26 (m, 1H), 4.04–3.89 (m, 1H), 3.71–3.35 (m, 2H), 2.25 (s, 1H), 1.53–1.41 (m, 18H), 1.30–1.14 (m, 3H); ^{13}C NMR (150 MHz, $CDCl_3$) δ 155.6 (C), 154.9 (C), 154.5 (C), 82.0 (C), 81.7 (C), 81.6 (C), 81.2 (C), 81.1 (C), 81.0 (C), 79.5 (C), 79.0 (C), 78.7 (C), 72.7 (C), 72.4 (C), 72.2 (C), 46.6 (CH_2), 44.7 (CH_2), 41.6 (CH_3), 39.6 (CH_3), 39.4 (CH_3), 28.4 (CH_3), 28.3 (CH_3), 28.3 (C), 28.2 (C), 13.7 (CH_3), 13.1 (CH_3), 13.0 (CH_3); IR (solid) 3262, 2977, 2934, 2126, 1709 cm^{-1}; LRMS (ES+) 299 (100, $[M+H]^+$); HRMS (ES+) calcd for $C_{15}H_{26}N_2O_4$ $[M+H]^+$ 299.1971, observed 299.1963.

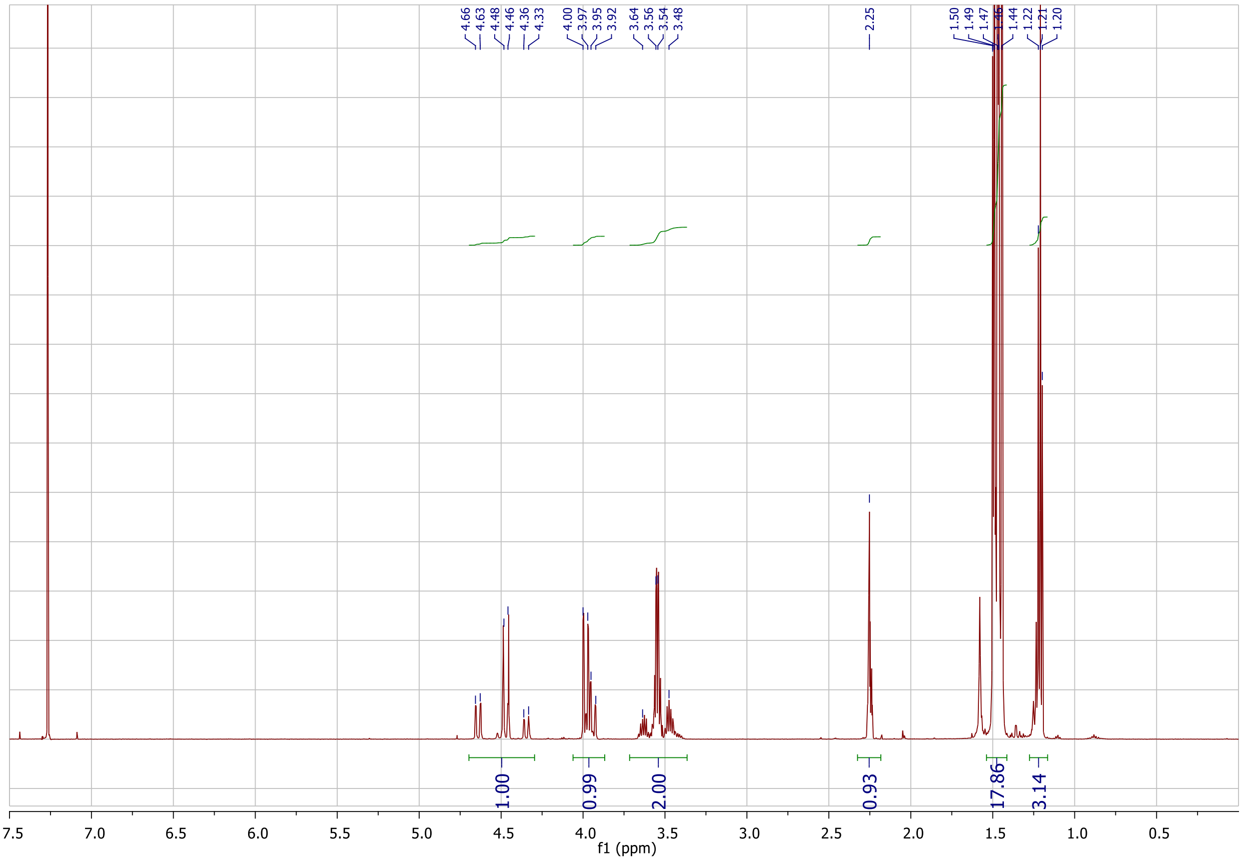

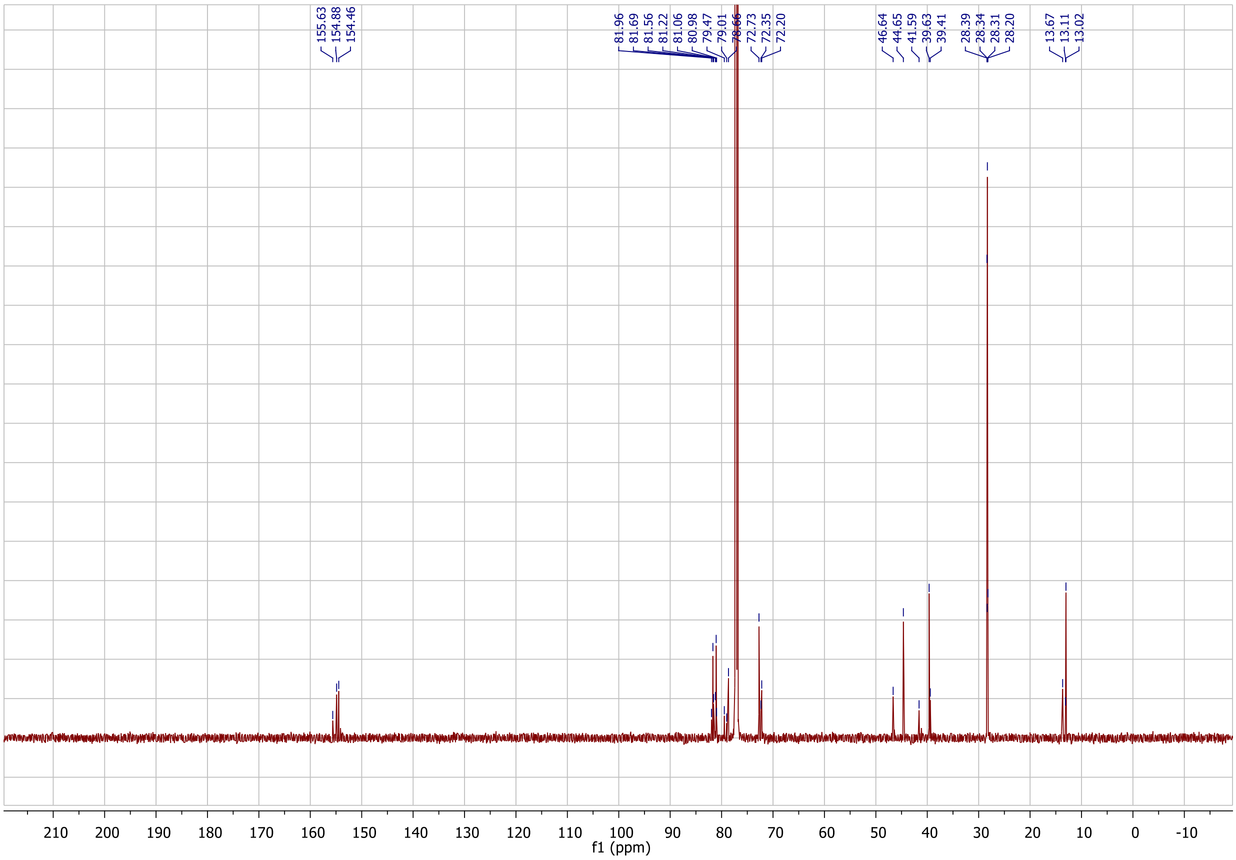

Figure **S10**. ^{1}H and ^{13}C NMR data for di-*tert*-butyl 1-ethyl-2-(prop-2-yn-1-yl)hydrazine-1,2-dicarboxylate.

4,5-Dibromo-1-ethyl-2-(prop-2-yn-1-yl)-1,2-dihydropyridazine-3,6-dione

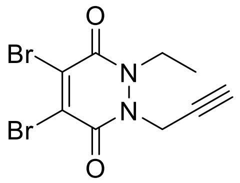

To a 1:1 solution of CH_2Cl_2:TFA (5 mL: 5 mL) was added di-*tert*-butyl 1-ethyl-2-(prop-2-yn-1-yl)hydrazine-1,2-dicarboxylate (259 mg, 0.87 mmol) and the reaction mixture stirred at 21 °C for 1 h. The reaction mixture was then concentrated *in vacuo*, taking care to ensure all the TFA was removed by use of toluene as an azeotrope. The crude residue was then dissolved in AcOH (10 mL), to which was added 3,4-dibromomaleic anhydride (222 mg, 0.87 mmol) and the reaction mixture heated under reflux for 2 h. The reaction mixture was then concentrated *in vacuo* and the crude residue purified by flash column chromatography (60% EtOAc:Pet.). The appropriate fractions were then combined and concentrated *in vacuo* to give 4,5-dibromo-1-ethyl-2-(prop-2-yn-1-yl)-1,2-dihydropyridazine-3,6-dione (124 mg, 0.37 mmol, 43%) as a pale yellow gum. ^{1}H NMR (600 MHz, $CDCl_3$) δ 4.89 (s, 2H), 4.29 (q, *J* = 7.1 Hz, 2H), 2.42 (t, *J* = 2.5 Hz, 1H), 1.32 (t, *J* = 7.1 Hz, 3H); ^{13}C NMR (150 MHz, $CDCl_3$) δ 153.6 (C), 152.9 (C), 137.3 (C), 135.2 (C), 75.6 (C), 75.0 (C), 43.0 (CH_2), 37.1 (CH_2), 13.2 (CH_3); IR (thin film) 3243, 2985, 2123, 1639, 1576 cm^{-1}; LRMS (EI) 338 (50, $[M^{81}Br^{81}Br+H]^+$), 336 (100, $[M^{81}Br^{79}Br+H]^+$), 334 (50, $[M^{79}Br^{79}Br+H]^+$); HRMS (EI) calcd for $C_9H_8Br_2N_2O_2$ $[M^{79}Br^{79}Br]^+$ 333.8953, observed 333.8950.

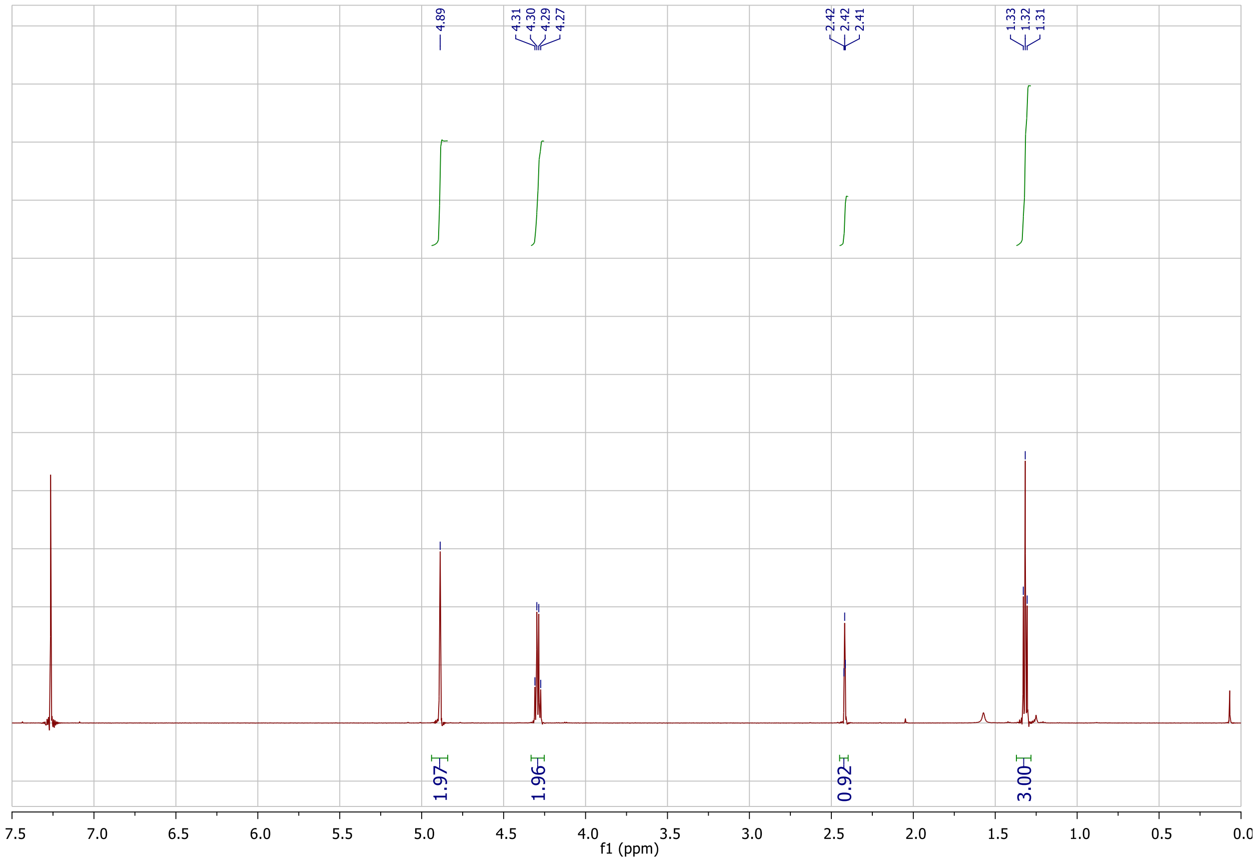

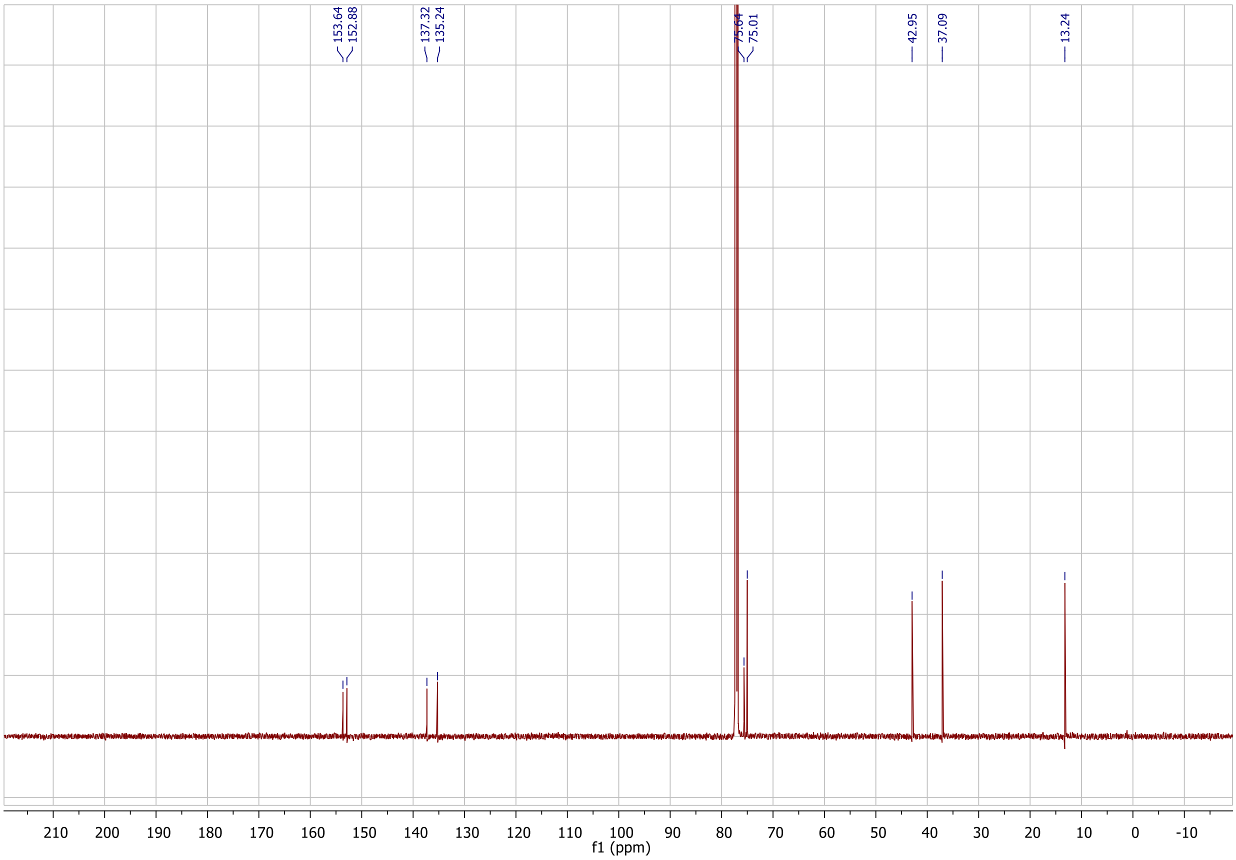

Figure **S11**. ^{1}H and ^{13}C NMR data for 4,5-dibromo-1-ethyl-2-(prop-2-yn-1-yl)-1,2-dihydropyridazine-3,6-dione.

4,5-Bis((4-aminophenyl)thio)-1-ethyl-2-(prop-2-yn-1-yl)-1,2-dihydropyridazine-3,6-dione

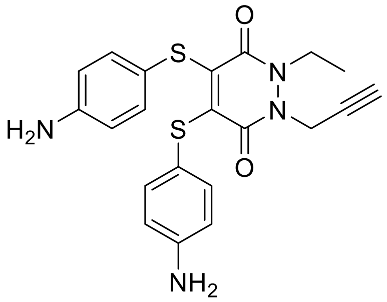

To a solution of 4,5-dibromo-1-ethyl-2-(prop-2-yn-1-yl)-1,2-dihydropyridazine-3,6-dione (124 mg, 0.37 mmol) and 4-aminothiophenol (185 mg, 1.49 mmol) in CH_2Cl_2 (15 mL), was added triethylamine (215 µL, 1.49 mmol), and the reaction mixture was left to stir at 21 °C for 18 h. The reaction mixture was then washed with deionised water (3 × 10 mL), and the organic phase was concentrated *in vacuo* and the crude residue purified by flash column chromatography (2–5% MeOH:CH_2Cl_2). The appropriate fractions were then combined and concentrated *in vacuo* to give 4,5-bis((4-aminophenyl)thio)-1-ethyl-2-(prop-2-yn-1-yl)-1,2-dihydropyridazine-3,6-dione (150 mg, 0.35 mmol, 95%) as an orange crystalline solid. m.p. 161–164 °C; ^{1}H NMR (600 MHz, $CDCl_3$) δ 7.17–7.12 (m, 4H), 6.62–6.56 (m, 4H), 4.71 (d, *J* = 2.5 Hz, 2H), 4.07 (q, *J* = 7.1 Hz, 1H), 3.77 (br. s, 4H), 2.31 (t, *J* = 2.5 Hz, 1H), 1.20 (t, *J* = 7.1 Hz, 3H); ^{13}C NMR (150 MHz, $CDCl_3$) δ 156.8 (C), 156.0 (C), 146.9 (C), 143.7 (C), 141.0 (C), 134.12 (C), 134.1 (C), 120.1 (C), 120.0 (C), 115.6 (C), 115.5 (C), 76.5 (C), 74.0 (CH), 41.5 (CH_2), 36.2 (CH_2), 12.7 (CH_3); IR (solid) 3450, 3359, 3275, 2128, 1619, 1595, 1517, 1495 cm^{-1}; LRMS (ES+) 425 (100, $[M+H]^+$); HRMS (ES+) calcd for $C_{21}H_{20}N_4O_2S_2$ $[M+H]^+$ 425.1106, observed 425.1107.

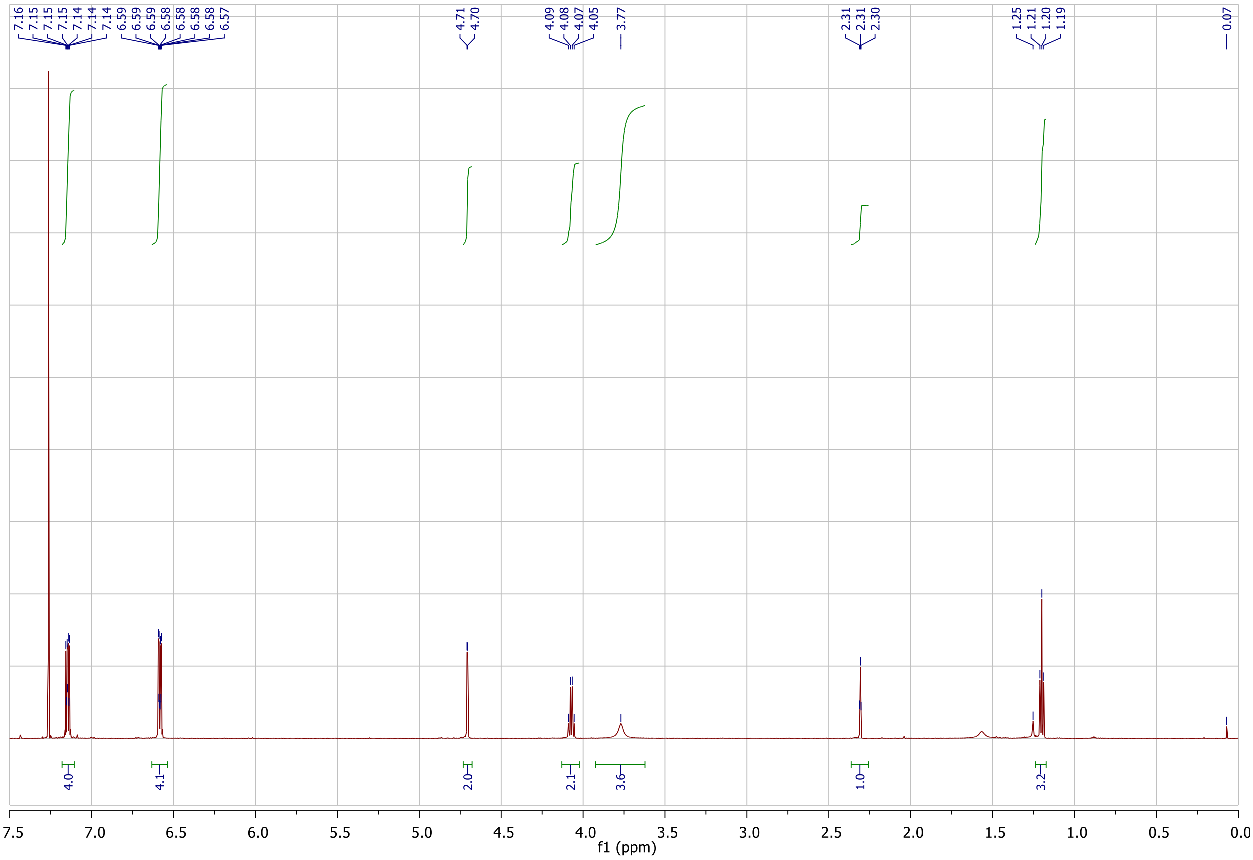

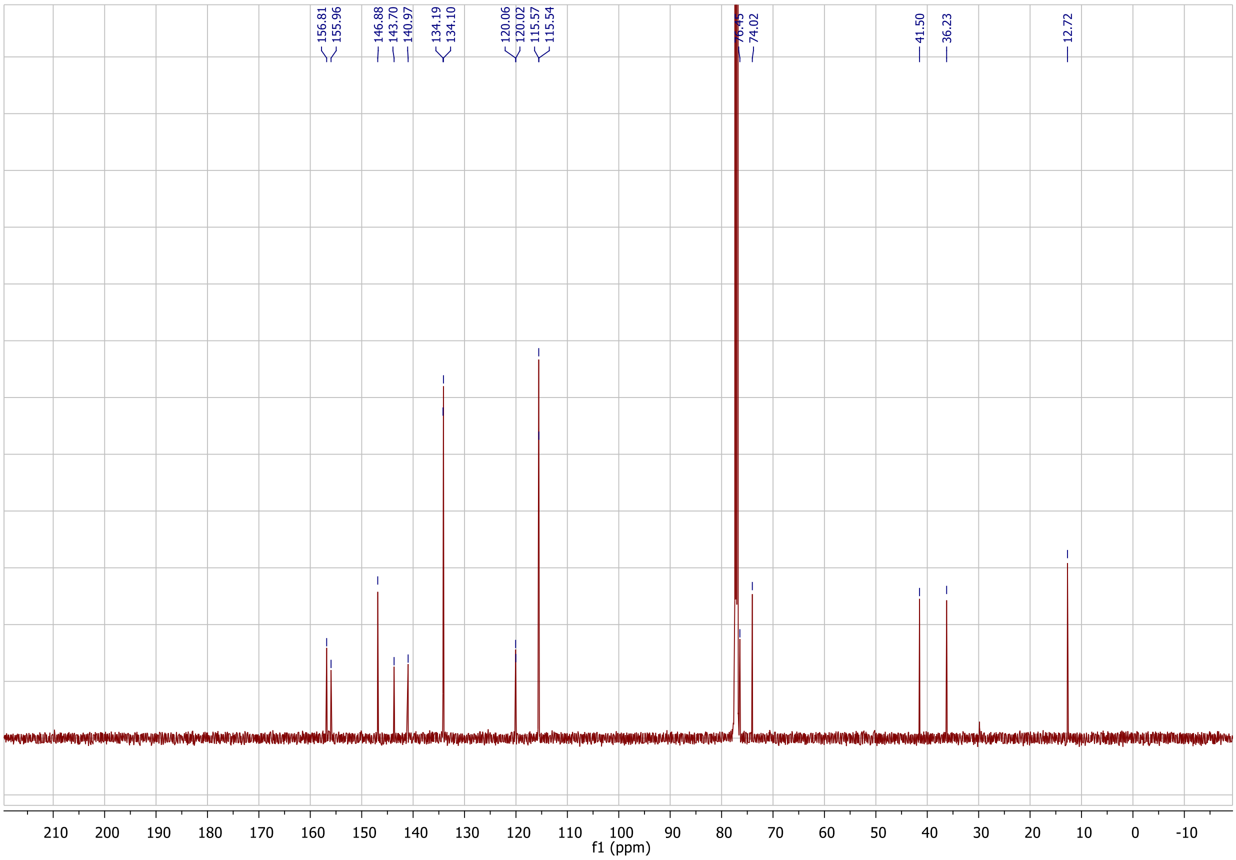

Figure **S12**. ^{1}H and ^{13}C NMR data for 4,5-bis((4-aminophenyl)thio)-1-ethyl-2-(prop-2-yn-1-yl)-1,2-dihydropyridazine-3,6-dione.

Tetramethyl 3,3',3'',3'''-((((((1-ethyl-3,6-dioxo-2-(prop-2-yn-1-yl)-1,2,3,6-tetrahydropyridazine-4,5-diyl)bis(sulfanediyl))bis(4,1-phenylene))bis(azanediyl))bis(3-oxopropane-3,1-diyl))bis(phosphanetriyl))tetrapropionate (Pyridazinedione 7)

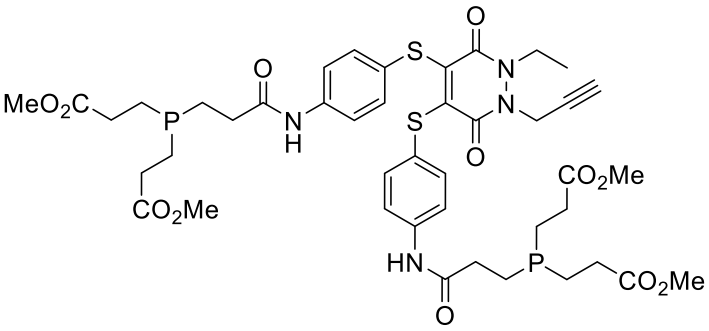

To a solution of 3-(bis(3-methoxy-3-oxopropyl)phosphanyl)propanoic acid (170 mg, 0.61 mmol) in DMF (10 mL), was added HATU (232 mg, 0.61 mmol) and the reaction mixture stirred at 21 °C for 15 mins. After this time, to the reaction mixture was added 4,5-bis((4-aminophenyl)thio)-1-ethyl-2-(prop-2-yn-1-yl)-1,2-dihydropyridazine-3,6-dione (118 mg, 0.28 mmol) and the reaction mixture stirred for a further 5 mins. Following this, to the reaction mixture was added *N*,*N*-diisopropylethylamine (DIPEA) (195 µL, 1.12 mmol), and the mixture left to stirr at 21 °C for 12 h. After this time, the reaction mixture was diluted with EtOAc (20 mL) and washed with deionised water (3 × 10 mL) and brine (3 × 10 mL). The organic phase was then concentrated *in vacuo* and the crude residue purified by flash column chromatography (0–2% MeOH:CH_2Cl_2). The appropriate fractions were then combined and concentrated *in vacuo* to give pyridazinedione **7** (84 mg, 0.09 mmol, 32%) as a yellow oil. ^{1}H NMR (600 MHz, $CDCl_3$) δ 7.53–7.41 (m, 3H), 7.25–7.02 (m, 5H), 6.59 (d, J = 8.4 Hz, 2H), 4.82–4.65 (m, 3H), 4.21–3.94 (m, 4H), 3.80–3.59 (m, 12H), 2.99–1.74 (m, 24H), 1.33–1.16 (m, 6H); ^{13}C NMR (150 MHz, $CDCl_3$) δ 174.0 (C), 156.7 (C), 155.8 (C), 147.3 (C), 137.9 (C), 134.2 (C), 132.0 (CH), 120.0 (CH), 115.7 (CH), 76.3 (C), 74.2 (CH), 52.5 (CH_2), 52.1 (CH_2), 41.7 (CH_2), 36.3 (CH_2), 30.5 (CH_2), 29.8 (CH_2), 26.3 (CH_3), 23.8 (CH_2), 21.4 (CH_2), 12.8 (CH_3); IR (thin film) 3212, 2925, 2852, 2120, 1734, 1687, 1624, 1531, 1494 cm^{-1}; LRMS (ES+) 944 (100, $[M+H]^+$); HRMS (ES+) calcd for $C_{43}H_{54}N_4O_{12}P_2S_2$ $[M+H]^+$ 944.2655, observed 944.2651.

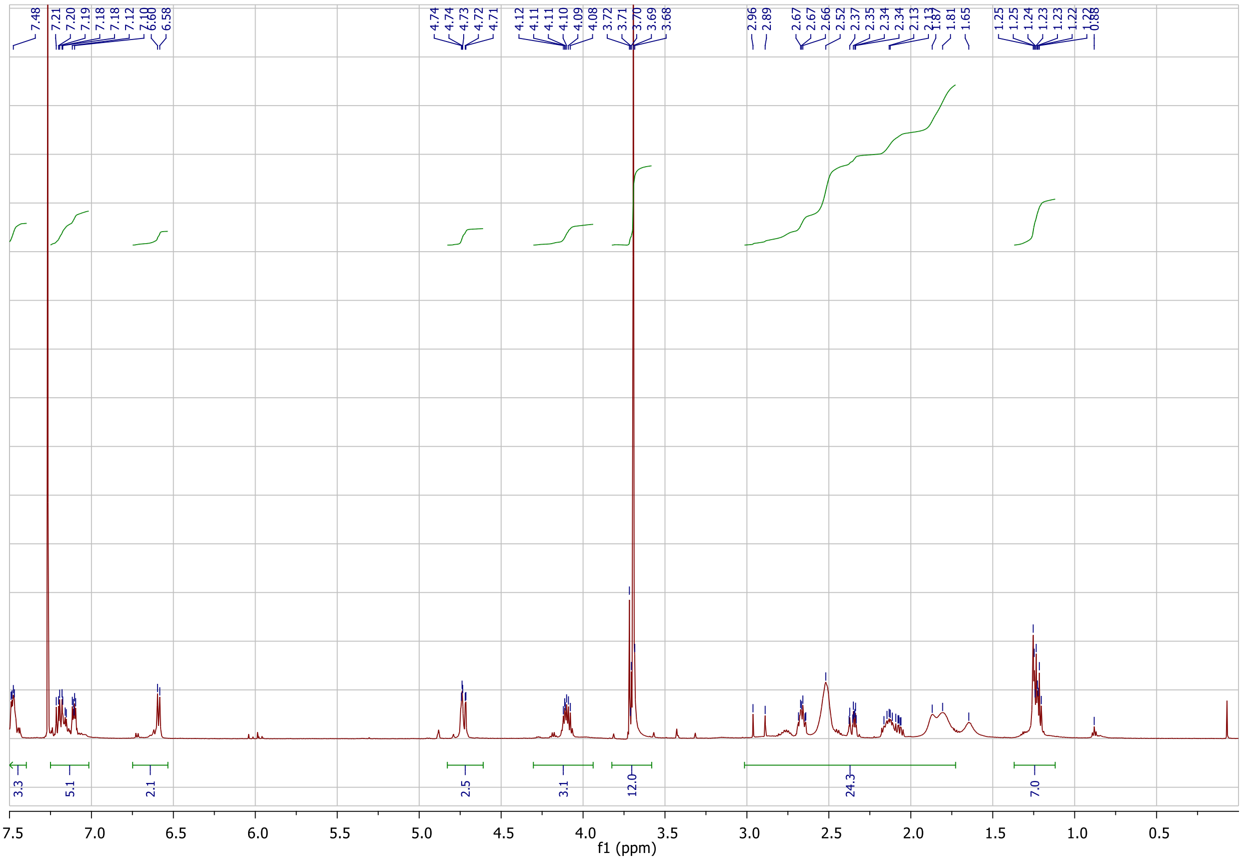

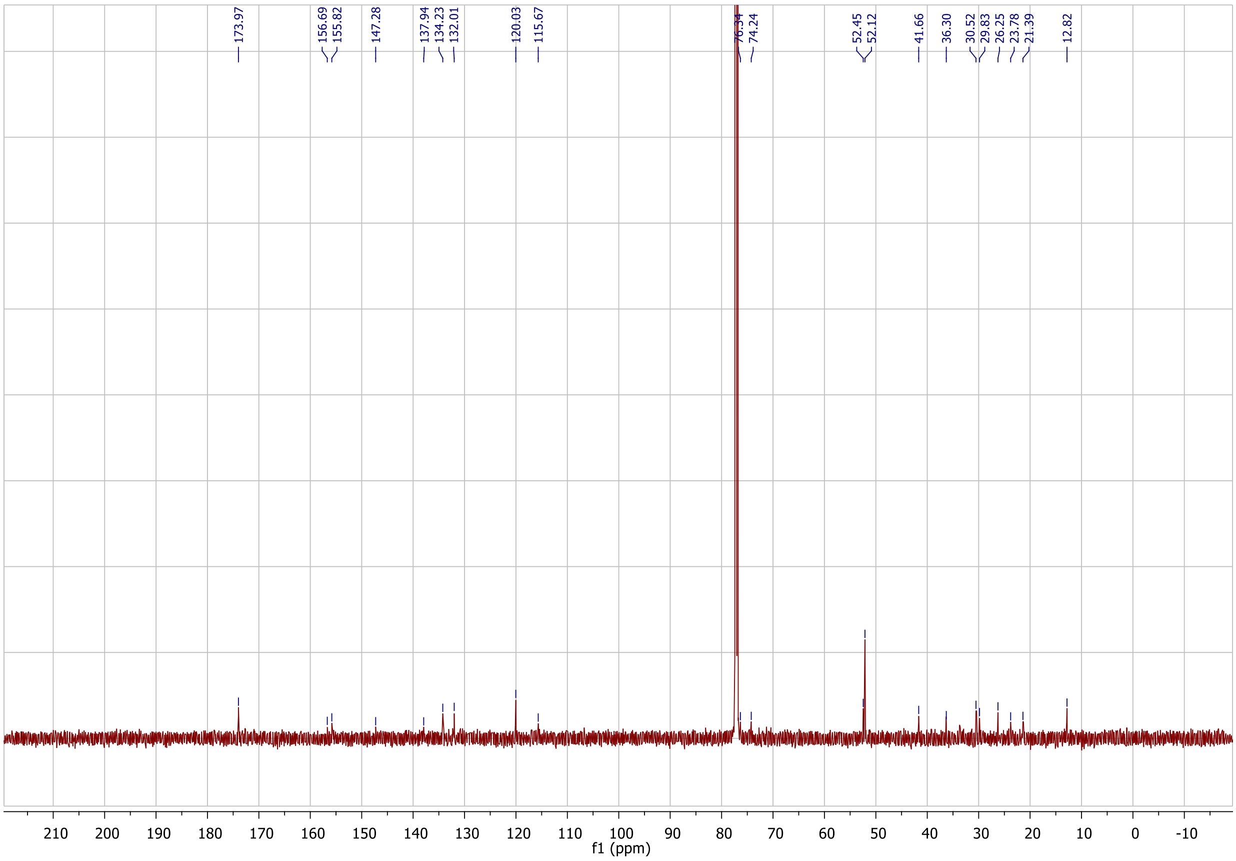

Figure **S13**. ^{1}H and ^{13}C NMR data for tetramethyl 3,3',3'',3'''-((((((1-ethyl-3,6-dioxo-2-(prop-2-yn-1-yl)-1,2,3,6-tetrahydropyridazine-4,5-diyl)bis(sulfanediyl))bis(4,1-phenylene))bis(azanediyl))bis(3-oxopropane-3,1-diyl))bis(phosphanetriyl))tetrapropionate (Pyridazinedione **7**).

Trastuzumab Fab fragment (Fab fragment) preparation[3]

Preparation of Fab fragment using sequential digests with pepsin and papain

Immobilized pepsin (0.15 mL) was washed with digestion buffer (20 mM sodium acetate trihydrate, pH 3.1) four times and trastuzumab (0.5 mL, 6.41 mg·mL^{-1} in digestion buffer) was added. The mixture was incubated for 5 h at 37 °C whilst shaking (1100 rpm). The resin was separated from the digest using a filter column, and washed with digest buffer (50 mM phosphate, 1 mM EDTA, 150 mM NaCl, pH 6.8) three times. The digest was combined with the washes and the volume adjusted to 0.5 mL.

After this, immobilized papain (0.5 mL, 0.25 mg·mL^{-1}) was activated with 10 mM DTT (in digest buffer: 50 mM phosphate, 1 mM EDTA, 150 mM NaCl, pH 6.8) whilst shaking (1100 rpm) for 1 h at 37 °C. The resin was washed with digest buffer (without DTT) four times and the 0.5 mL of Herceptin-F(ab')$_2$ added. The mixture was incubated for 16 h at 37 °C whilst shaking (1100 rpm). Then the resin was separated from the digest using a filter column, and washed with BBS (25 mM sodium borate, 25 mM NaCl, 0.5 mM EDTA, pH 8.0) three times. The digest was combined with the washes and the buffer was exchanged completely for BBS using diafiltration columns (GE Healthcare, 10000 MWCO) and the volume adjusted to 0.5 mL. The digest was analysed by SDS-PAGE and LCMS to reveal formation of a single trastuzumab Fab fragment: observed mass 47585. The concentration of Fab fragment **2** was determined by UV/VIS using a molecular extinction coefficient of ε_{280} = 68590 M^{-1}·cm^{-1}. [Fab-Her fragment] 2.6 mg·mL^{-1} (0.5 mL), 62%.

Bioconjugation reactions involving Fab fragment of Herceptin

Control for untreated Fab fragment of Herceptin for experiments using pyridazinediones 5 and 6

(a)

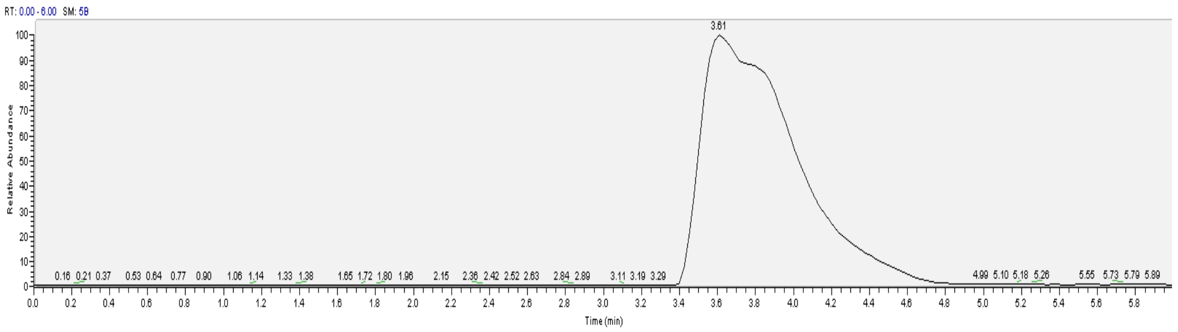

(b)

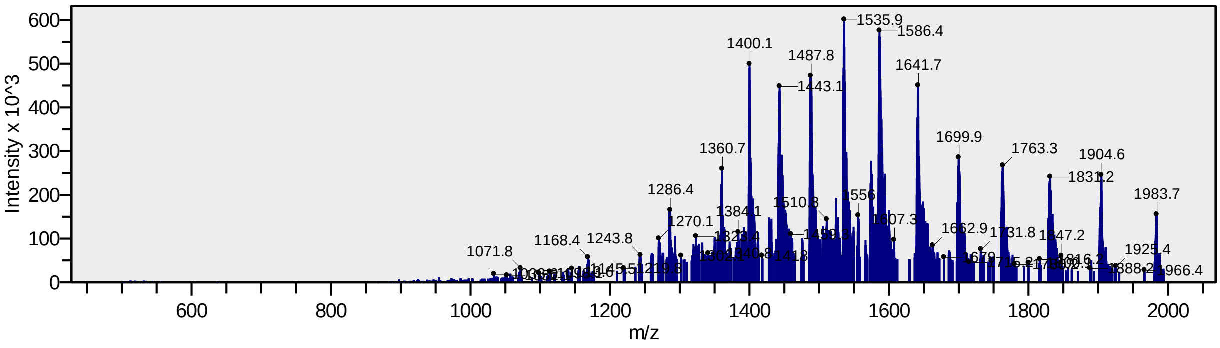

(c)

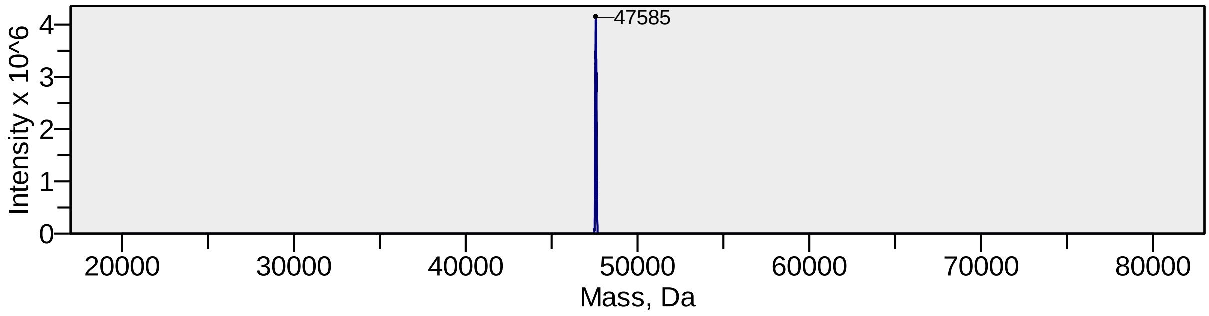

Figure **S14**. (a) TIC, (b) non-deconvoluted and (c) deconvoluted MS data for untreated Fab fragment of Herceptin for experiments using pyridazinediones **5** and **6**.

Reaction of Fab fragment of Herceptin with pyridazinedione 5 (1.25 eq.)

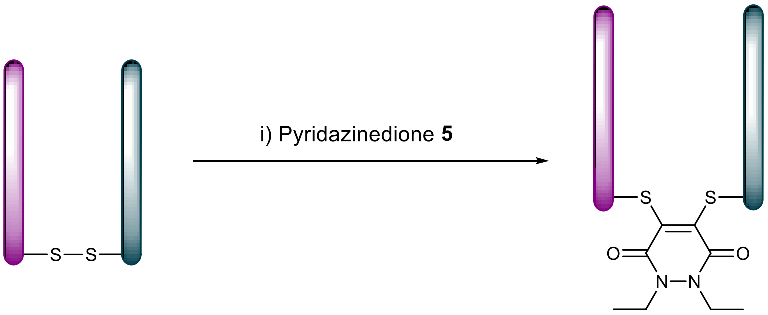

Pyridazinedione **5** (0.56 µL, 2.0 mM in DMF, 1.25 eq) was added to Fab fragment of Herceptin (30 µL, 1.4 mg/mL, 30 µM) in BBS (25 mM sodium borate, 25 mM NaCl, 0.5 mM EDTA, pH 8.0). The reaction mixture was incubated at 37 °C for 1 h. Excess reagents were removed by repeated diafiltration into deionised water using VivaSpin sample concentrators (GE Healthcare, 10,000 MWCO). The samples were analysed by LCMS. Expected mass: 47,751 Da. Observed mass: 47,755 Da.

(a)

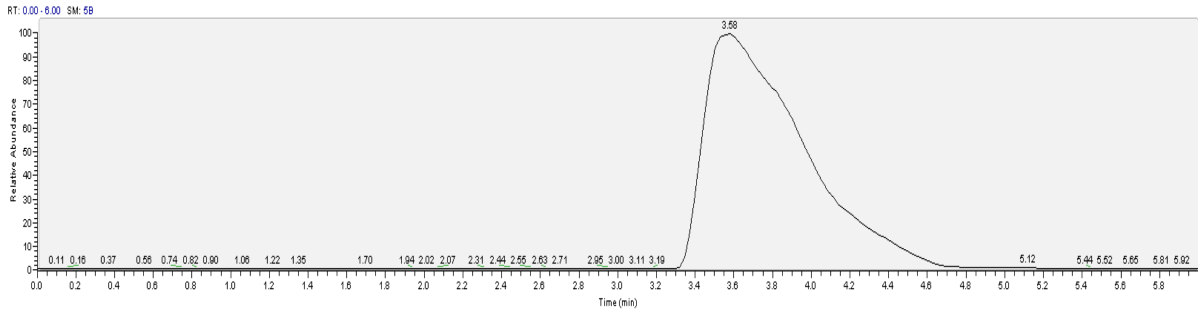

(b)

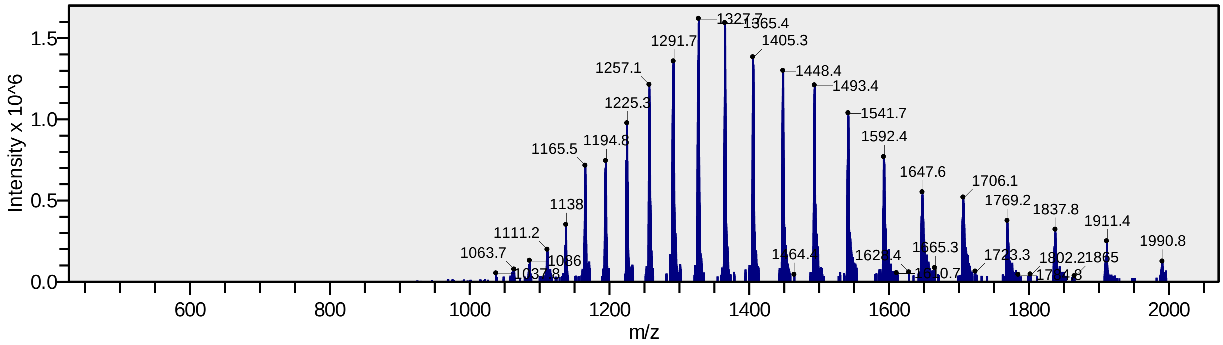

(c)

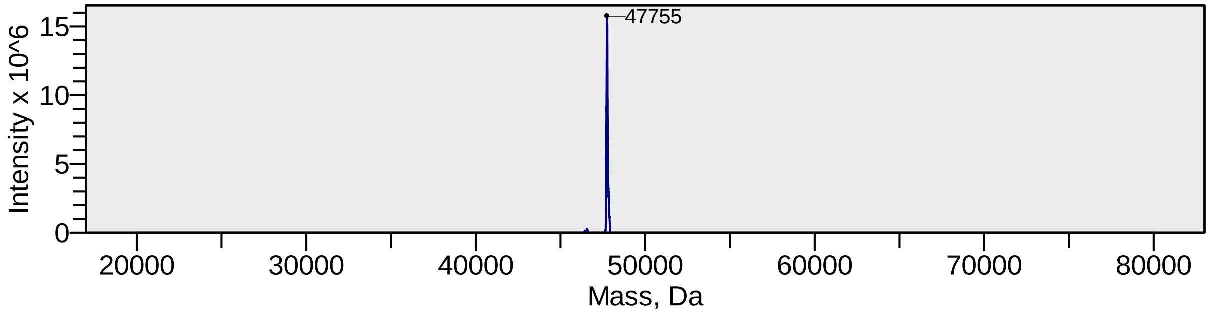

Figure **S15**. (a) TIC, (b) non-deconvoluted and (c) deconvoluted MS data for Fab fragment of Herceptin reacted with pyridazinedione **5** (1.25 eq.).

Reaction of Fab fragment of Herceptin with pyridazinedione 5 (2 eq.)

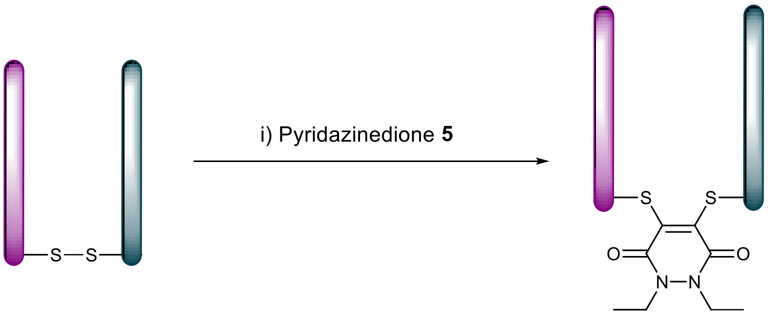

Pyridazinedione **5** (0.9 µL, 2.0 mM in DMF, 2 eq) was added to Fab fragment of Herceptin (30 µL, 1.4 mg/mL, 30 µM) in BBS (25 mM sodium borate, 25 mM NaCl, 0.5 mM EDTA, pH 8.0). The reaction mixture was incubated at 37 °C for 1 h. Excess reagents were removed by repeated diafiltration into deionised water using VivaSpin sample concentrators (GE Healthcare, 10,000 MWCO). The samples were analysed by LCMS. Expected mass: 47,751 Da. Observed mass: 47,743 Da.

(a)

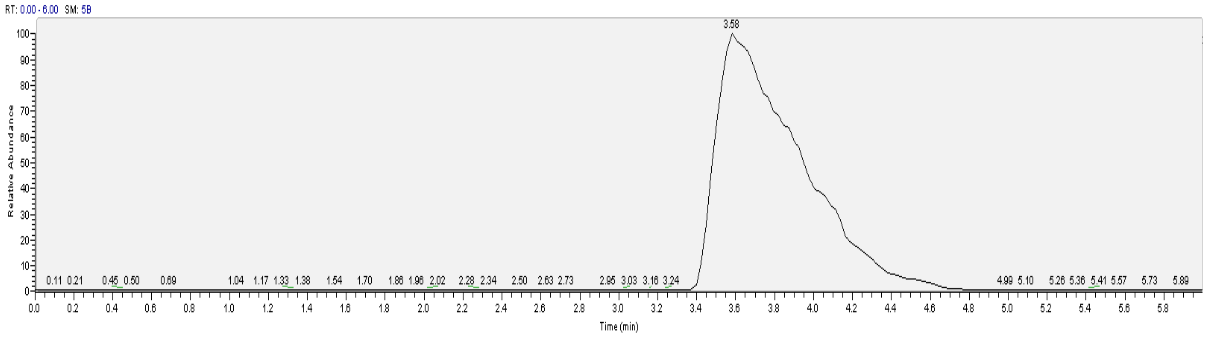

(b)

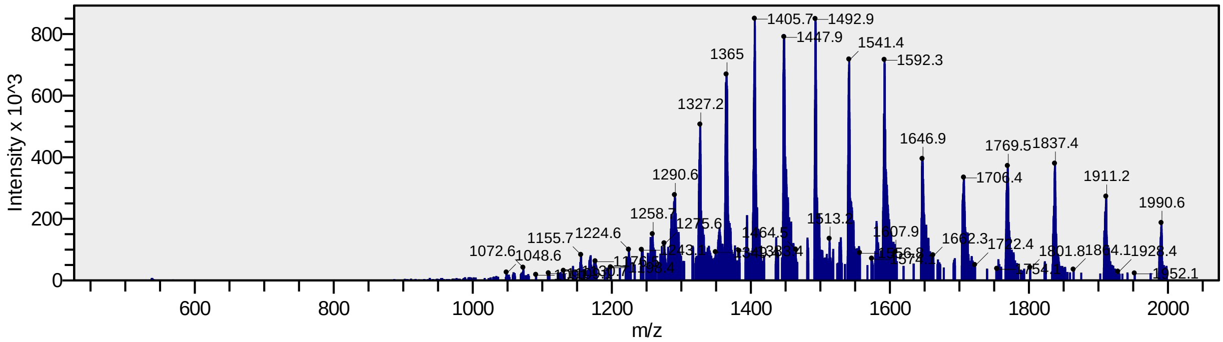

(c)

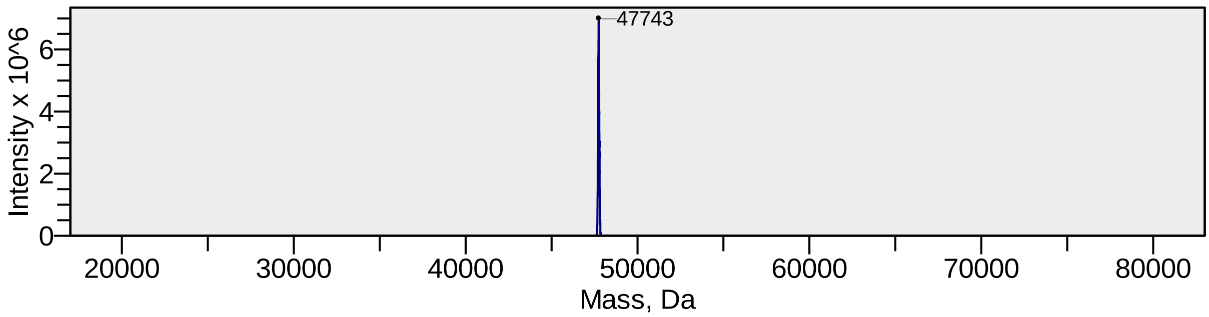

Figure **S16**. (a) TIC, (b) non-deconvoluted and (c) deconvoluted MS data for Fab fragment of Herceptin reacted with pyridazinedione **5** (2 eq.).

Reaction of Fab-Her fragment with pyridazinedione 5 (5 eq.)

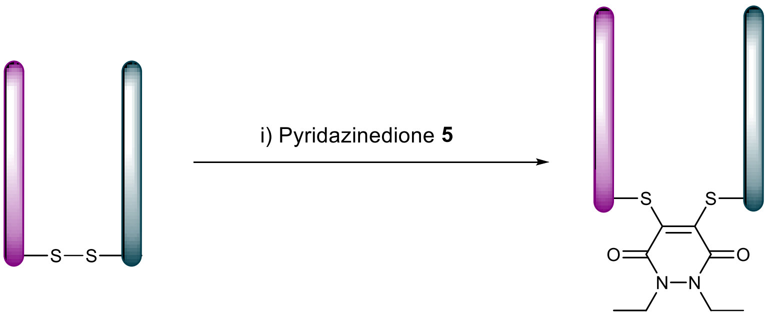

Pyridazinedione **5** (2.3 µL, 2.0 mM in DMF, 5 eq) was added to Fab fragment of Herceptin (30 µL, 1.4 mg/mL, 30 µM) in BBS (25 mM sodium borate, 25 mM NaCl, 0.5 mM EDTA, pH 8.0). The reaction mixture was incubated at 37 °C for 1 h. Excess reagents were removed by repeated diafiltration into

deionised water using VivaSpin sample concentrators (GE Healthcare, 10,000 MWCO). The samples were analysed by LCMS. Expected mass: 47,751 Da. Observed mass: 47,753 Da.

(a)

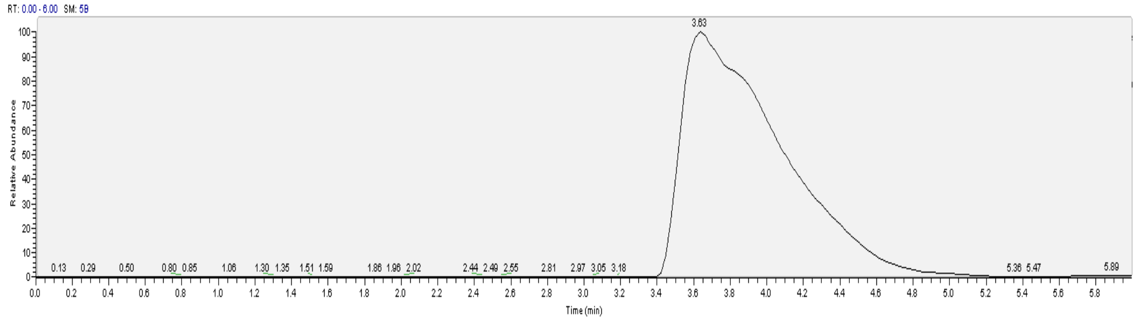

(b)

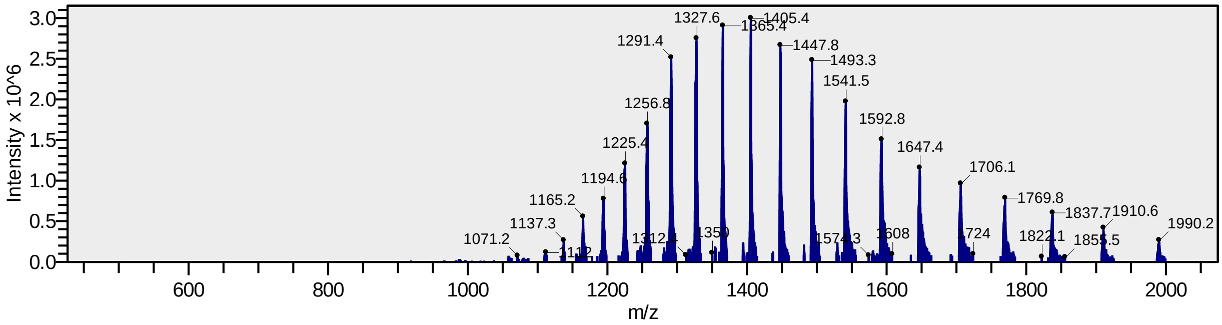

(c)

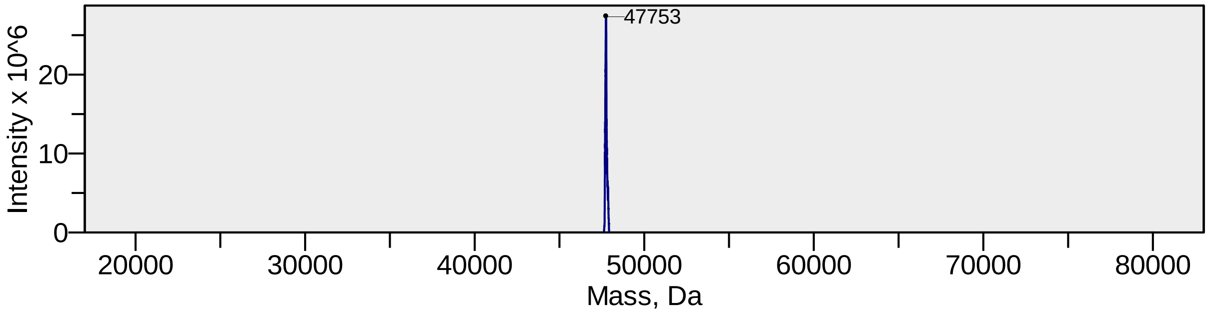

Figure **S17**. (a) TIC, (b) non-deconvoluted and (c) deconvoluted MS data for Fab fragment of Herceptin reacted with pyridazinedione **5** (5 eq.).

Reaction of Fab fragment of Herceptin with pyridazinedione 5 (10 eq.)

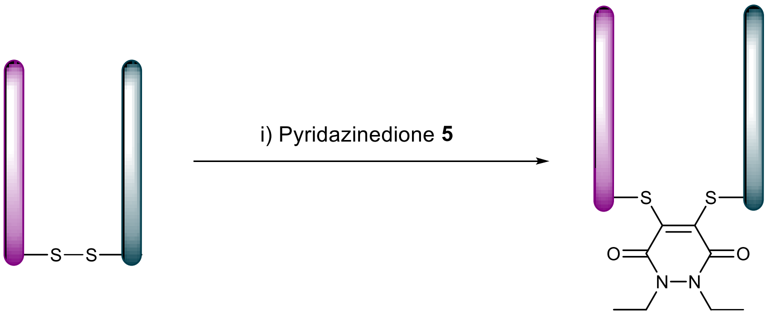

Pyridazinedione **5** (4.5 µL, 2.0 mM in DMF, 10 eq) was added to Fab fragment of Herceptin (30 µL, 1.4 mg/mL, 30 µM) in BBS (25 mM sodium borate, 25 mM NaCl, 0.5 mM EDTA, pH 8.0). The reaction mixture was incubated at 37 °C for 1 h. Excess reagents were removed by repeated diafiltration into deionised water using VivaSpin sample concentrators (GE Healthcare, 10,000 MWCO). The samples were analysed by LCMS. Expected mass: 47,751 Da. Observed mass: 47,750 Da.

(a)

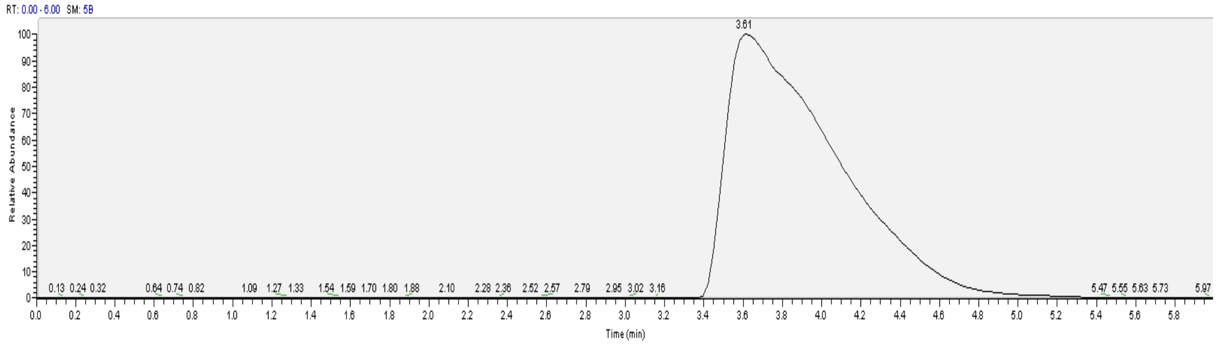

(b)

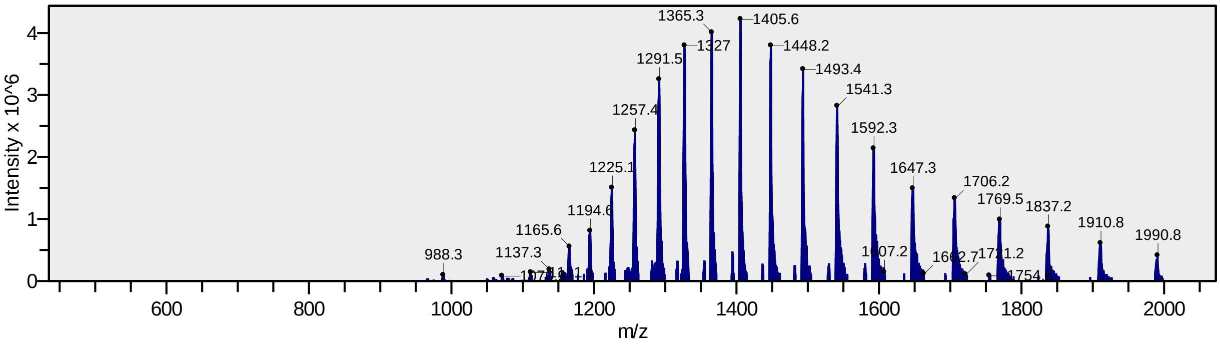

(c)

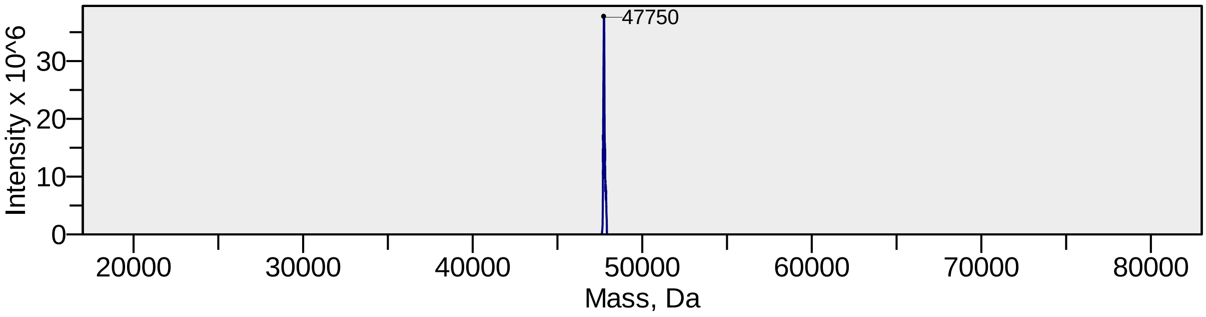

Figure **S18**. (a) TIC, (b) non-deconvoluted and (c) deconvoluted MS data for Fab fragment of Herceptin reacted with pyridazinedione **5** (10 eq.).

Reduction of Fab fragment of Herceptin and subsequent reaction with pyridazinedione 6

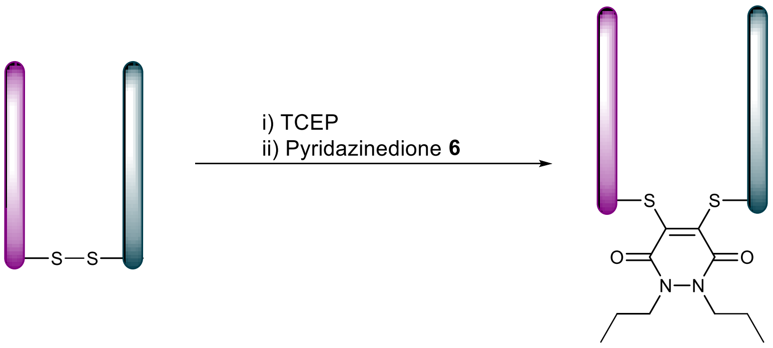

TCEP (4.5 µL, 2.0 mM, 10 eq) was added to the Fab fragment of Herceptin (30 µL, 1.4 mg/mL, 30 µM) in BBS (25 mM sodium borate, 25 mM NaCl, 0.5 mM EDTA, pH 8.0). The reaction was incubated at 37 °C for 1 h. Excess reagents were removed by repeated diafiltration into fresh BBS (25 mM sodium borate, 25 mM NaCl, 0.5 mM EDTA, pH 8.0) using VivaSpin sample concentrators (GE Healthcare, 10,000 MWCO). Pyridazinedione **6** (4.5 µL, 2.0 mM, 10 eq) was subsequently added to the reaction mixture and incubated at 37 °C for 1 h. Excess reagents were removed by repeated diafiltration into deionised water using VivaSpin sample concentrators (GE Healthcare, 10,000 MWCO). The samples were analysed by LCMS. Expected mass: 47,779 Da. Observed mass: 47,782 Da.

(a)

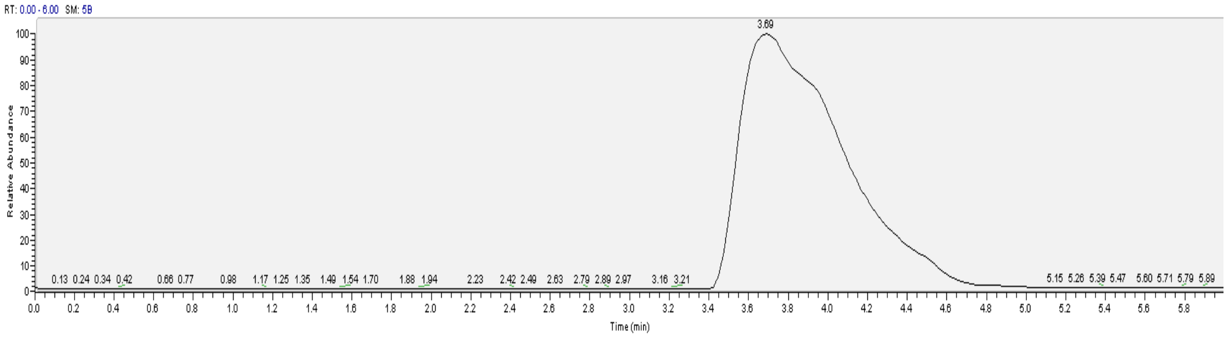

(b)

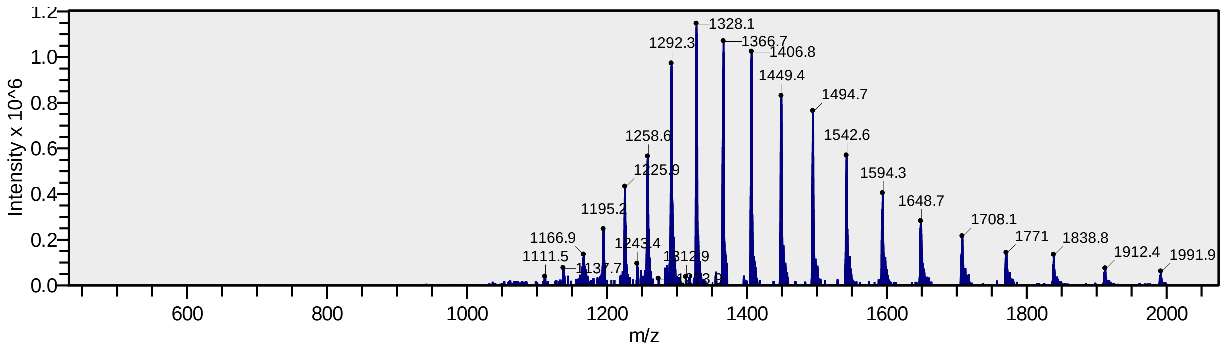

(c)

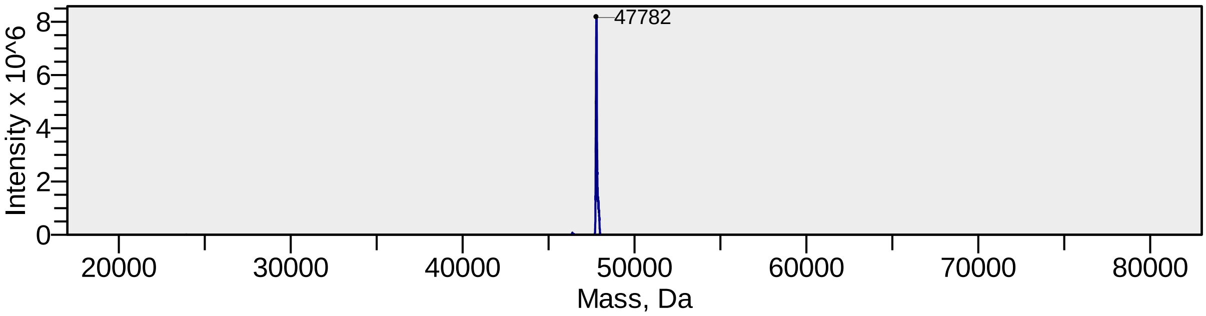

Figure **S19**. (a) TIC, (b) non-deconvoluted and (c) deconvoluted MS data for Fab fragment of Herceptin reacted with pyridazinedione **6**.

Reaction of Fab fragment of Herceptin with pyridazinedione 5 (2 eq.) in the presence of pre-incubated pyridazinedione 6 (1 eq.)

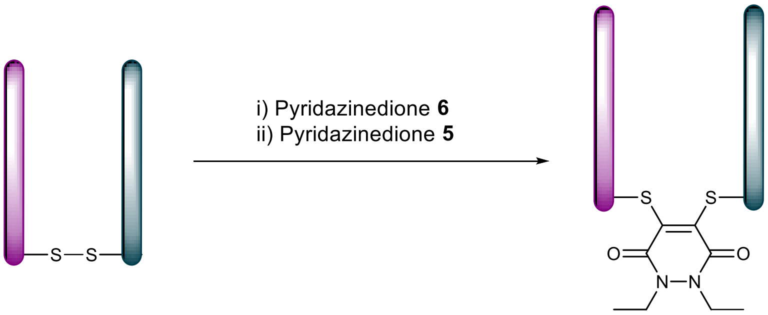

Pyridazinedione **5** (0.9 µL, 2.0 mM in DMF, 2 eq) was added to Fab fragment of Herceptin (30 µL, 1.4 mg/mL, 30 µM) in BBS (25 mM sodium borate, 25 mM NaCl, 0.5 mM EDTA, pH 8.0), which had been pre-incubated at 37 °C for 0.5 h with pyridazinedione **6** (0.45 µL, 2.0 mM in DMF, 1 eq). The reaction mixture was incubated further at 37 °C for 1 h. Excess reagents were removed by repeated diafiltration into deionised water using VivaSpin sample concentrators (GE Healthcare, 10,000 MWCO). The samples were analysed by LCMS. Expected mass: 47,751 Da. Observed mass: 47,745 Da.

(a)

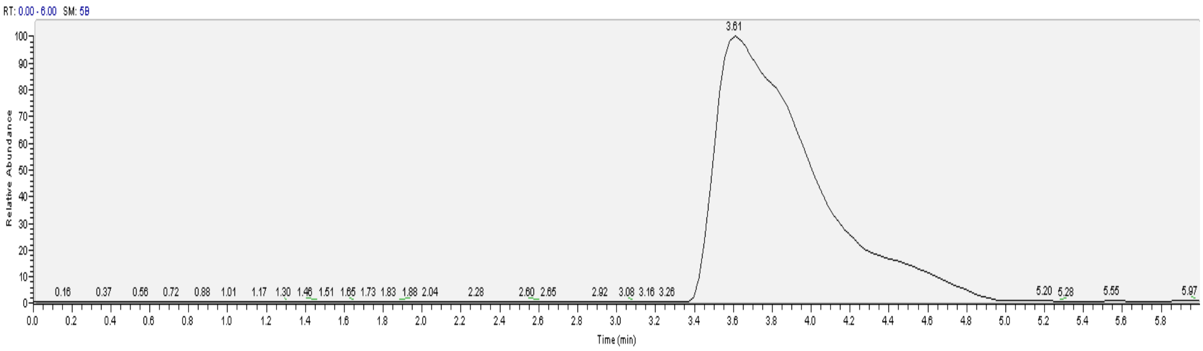

(b)

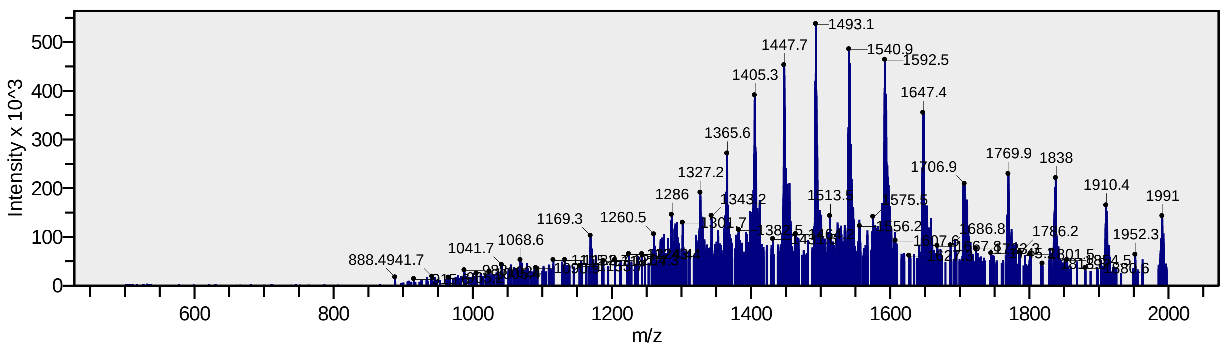

(c)

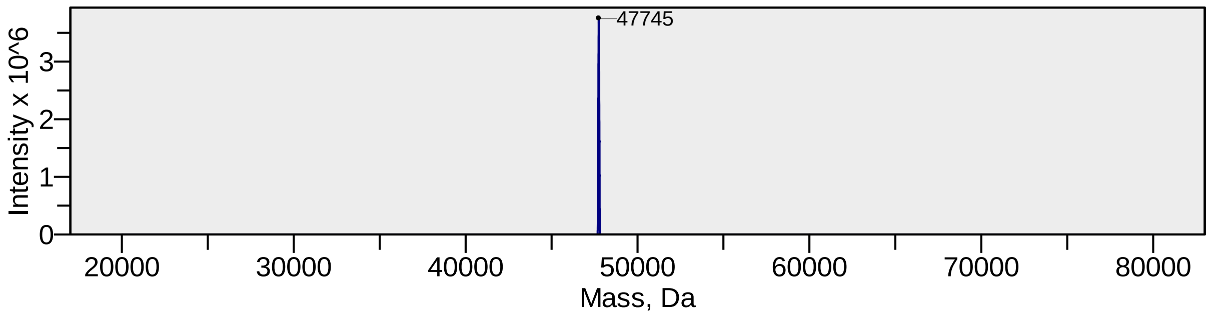

Figure **S20**. (a) TIC, (b) non-deconvoluted and (c) deconvoluted MS data for Fab fragment of Herceptin reacted with pyridazinedione **5** in the presence of pyridazinedione **6** (ratio of **5**:**6** = 2:1).

Reaction of Fab fragment of Herceptin with pyridazinedione 5 (2 eq.) in the presence of pre-incubated pyridazinedione 6 (2 eq.)

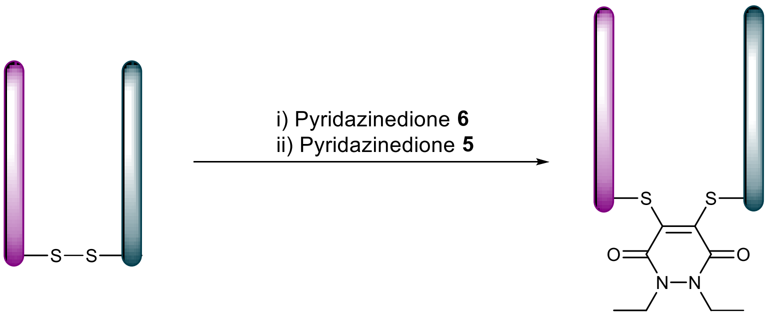

Pyridazinedione **5** (0.9 µL, 2.0 mM in DMF, 2 eq) was added to Fab fragment of Herceptin (30 µL, 1.4 mg/mL, 30 µM) in BBS (25 mM sodium borate, 25 mM NaCl, 0.5 mM EDTA, pH 8.0), which had been pre-incubated at 37 °C for 0.5 h with pyridazinedione **6** (0.9 µL, 2.0 mM in DMF, 2 eq). The reaction mixture was incubated further at 37 °C for 1 h. Excess reagents were removed by repeated diafiltration into deionised water using VivaSpin sample concentrators (GE Healthcare, 10,000 MWCO). The samples were analysed by LCMS. Expected mass: 47,751 Da. Observed mass: 47,749 Da.

(a)

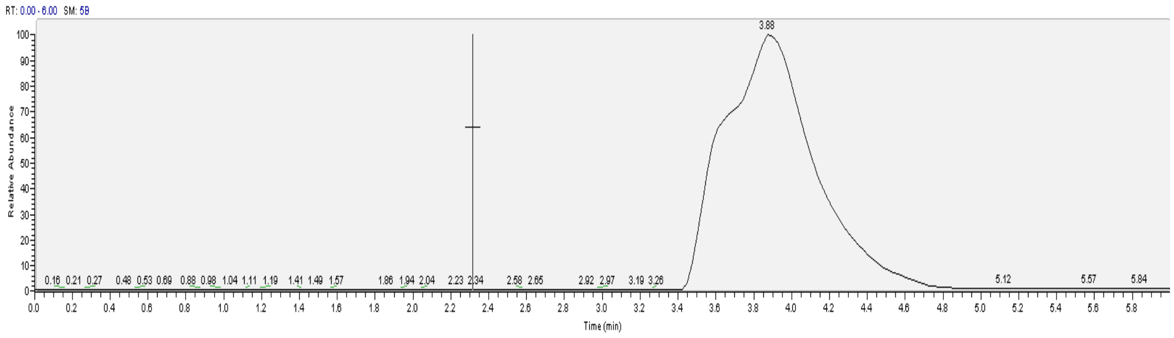

(b)

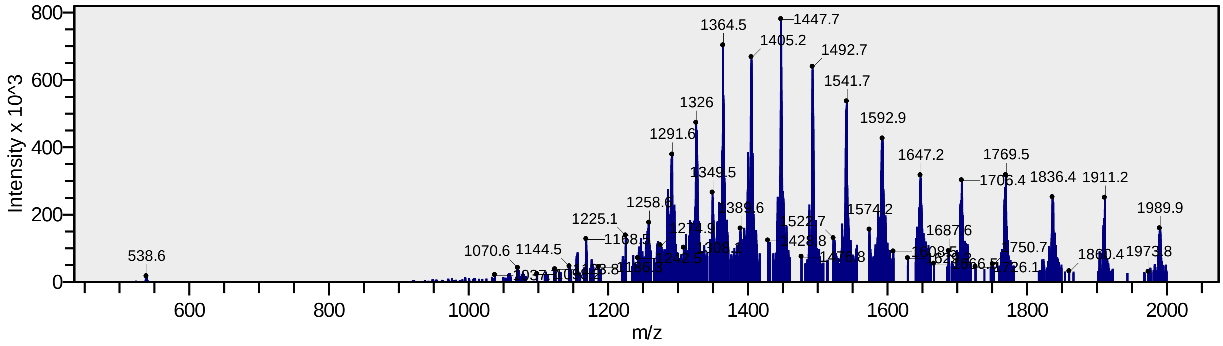

(c)

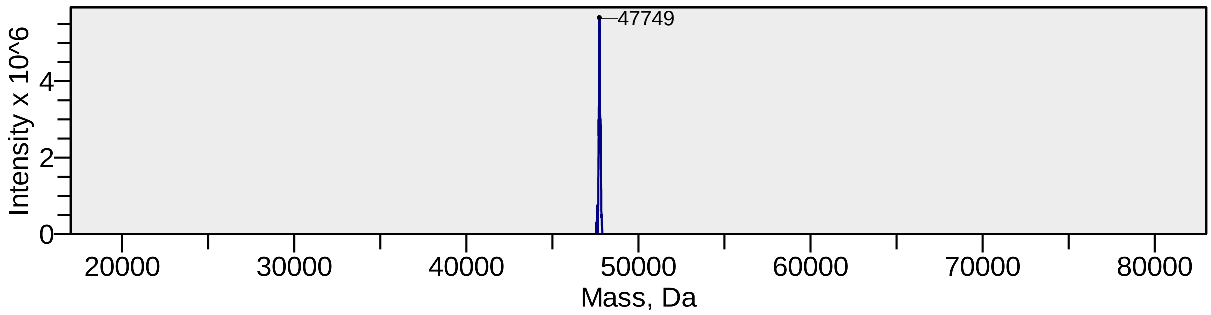

Figure **S21**. (a) TIC, (b) non-deconvoluted and (c) deconvoluted MS data for Fab fragment of Herceptin reacted with pyridazinedione **5** in the presence of pyridazinedione **6** (ratio of **5**:**6** = 2:2).

Reaction of Fab fragment of Herceptin with pyridazinedione 5 (2 eq.) in the presence of pre-incubated pyridazinedione 6 (5 eq.)

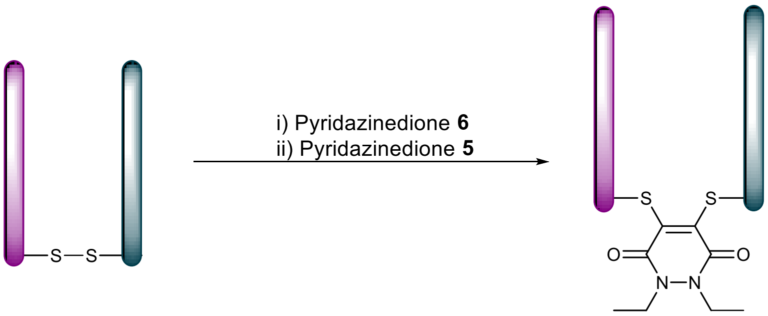

Pyridazinedione **5** (0.9 µL, 2.0 mM in DMF, 2 eq) was added to Fab fragment of Herceptin (30 µL, 1.4 mg/mL, 30 µM) in BBS (25 mM sodium borate, 25 mM NaCl, 0.5 mM EDTA, pH 8.0), which had

been pre-incubated at 37 °C for 0.5 h with pyridazinedione **6** (2.3 µL, 2.0 mM in DMF, 5 eq). The reaction mixture was incubated further at 37 °C for 1 h. Excess reagents were removed by repeated diafiltration into deionised water using VivaSpin sample concentrators (GE Healthcare, 10,000 MWCO). The samples were analysed by LCMS. Expected mass: 47,751 Da. Observed mass: 47,751 Da.

(a)

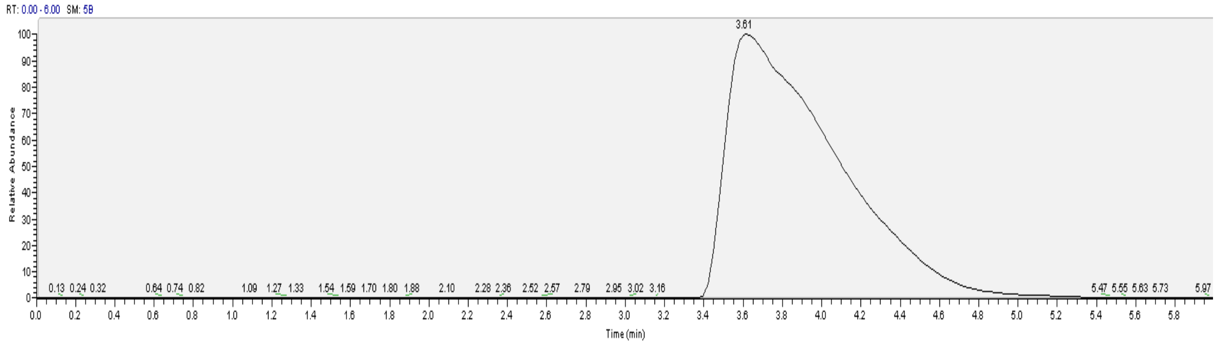

(b)

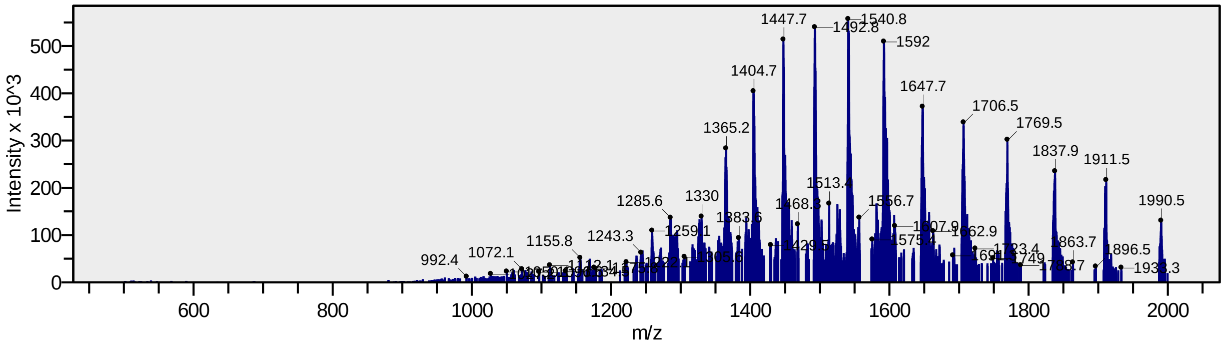

(c)

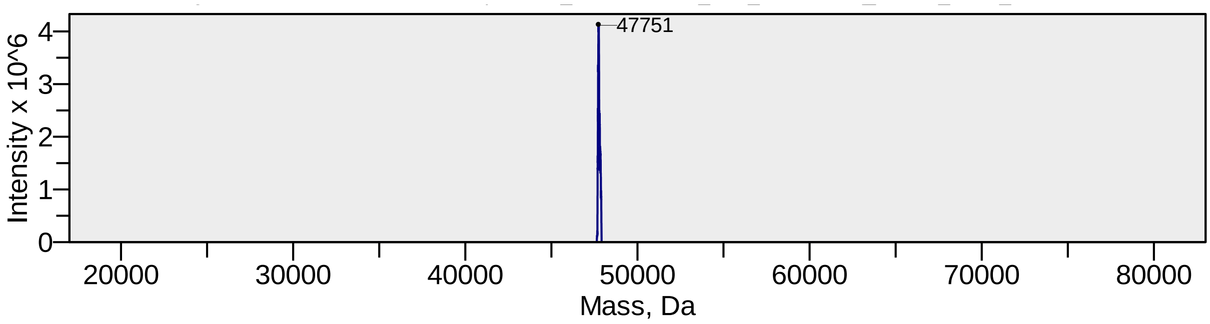

Figure **S22**. (a) TIC, (b) non-deconvoluted and (c) deconvoluted MS data for Fab fragment of Herceptin reacted with pyridazinedione **5** in the presence of pyridazinedione **6** (ratio of **5**:**6** = 2:5).

Reduction of Fab fragment of Herceptin and subsequent reaction with pyridazinedione 6 and pyridazinedione 5 (1:1 ratio 0.5 eq.:0.5 eq)

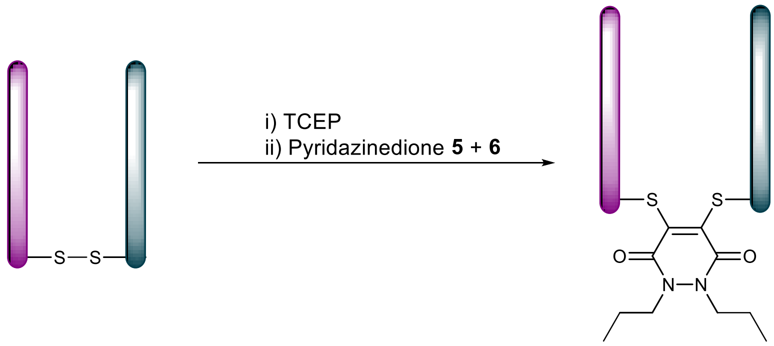

TCEP (4.5 µL, 2.0 mM, 10 eq) was added to the Fab fragment of Herceptin (30 µL, 1.4 mg/mL, 30 µM) in BBS (25 mM sodium borate, 25 mM NaCl, 0.5 mM EDTA, pH 8.0). The reaction was incubated at 37 °C for 1.0 h. Excess reagents were removed by repeated diafiltration into fresh BBS (25 mM sodium borate, 25 mM NaCl, 0.5 mM EDTA, pH 8.0) using VivaSpin sample concentrators (GE Healthcare, 10,000 MWCO). Pre-mixed pyridazinediones **5** and **6** ([1.1 µL, 0.2 mM in DMF, 0.5 eq] each) were subsequently added to the reaction mixture and incubated at 37 °C for 1.0 h. Excess reagents were removed by repeated diafiltration into deionised water using VivaSpin sample concentrators (GE Healthcare, 10,000 MWCO). The samples were analysed by LCMS. Expected mass: 47,751 Da [Fab fragment reacted with pyridazinedione **5**] and 47,779 Da [Fab fragment reacted with pyridazinedione **6**]. Observed mass:47,585 Da [unreacted Fab fragment], 47,746 Da [Fab fragment reacted with pyridazinedione **5**] and 47,776 Da [Fab fragment reacted with pyridazinedione **6**].

(a)

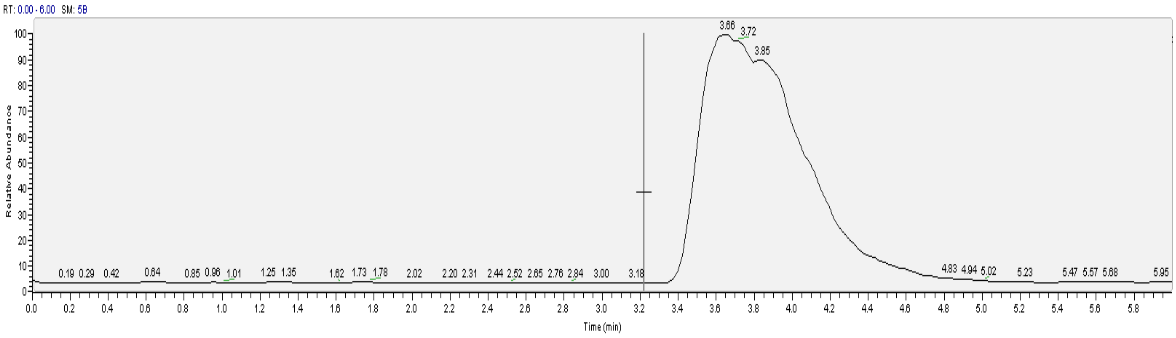

(b)

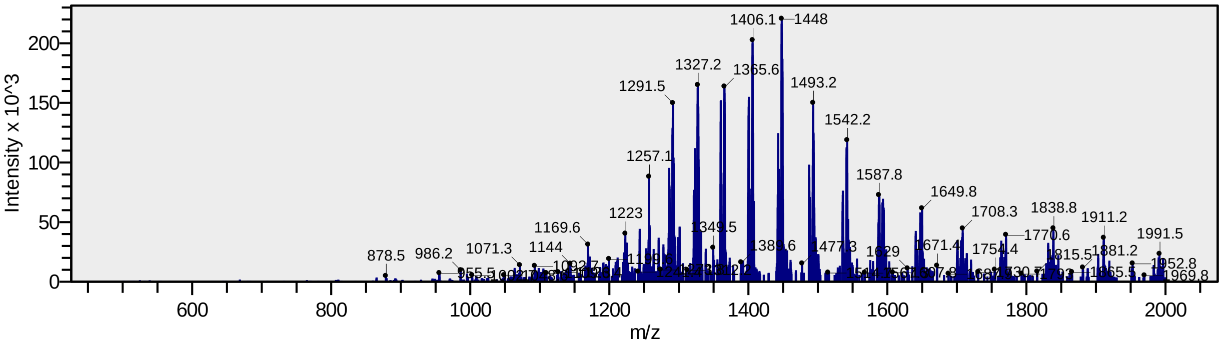

(c)

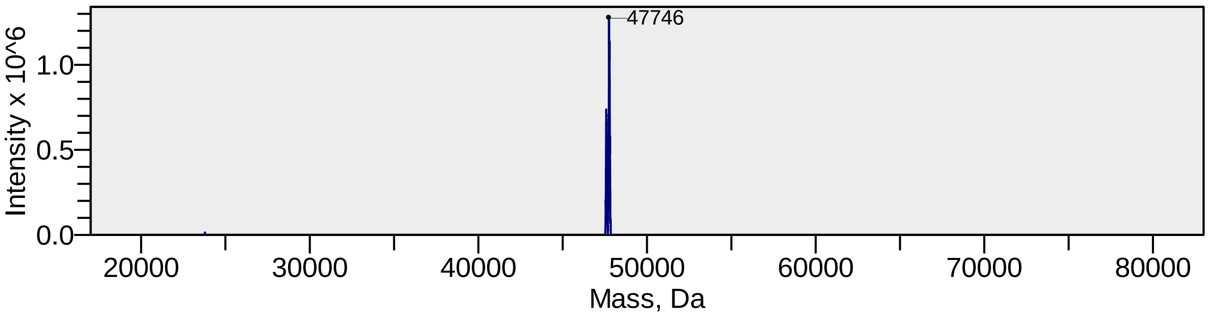

(d)

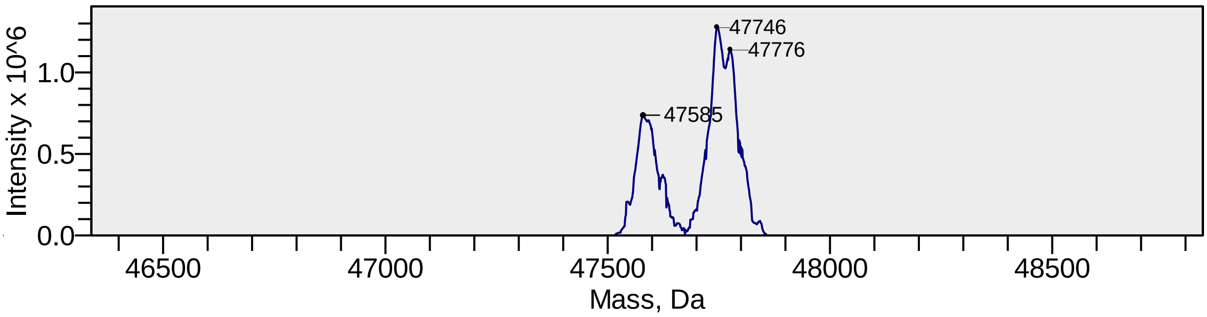

Figure **S23**. (a) TIC, (b) non-deconvoluted, (c) deconvoluted MS data over range 20,000–80,000 Da and (d) deconvoluted data over range 46,500–48,500 Da for reduction of Fab fragment of Herceptin and subsequent reaction with pyridazinedione **5** and pyridazinedione **6** (1:1 ratio 0.5 eq.:0.5 eq).

Reaction of Octreotide peptide with pyridazinedione 5 (1.25 eq.)

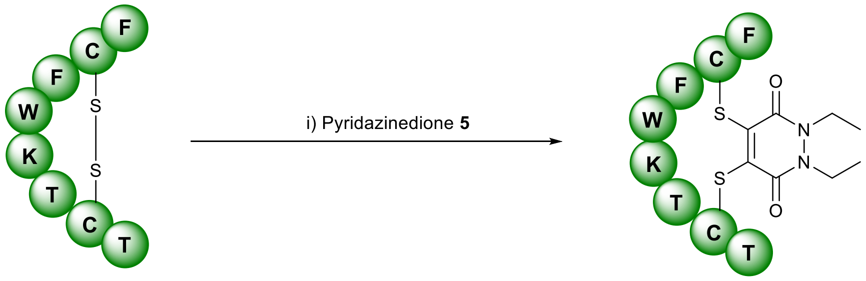

Pyridazinedione **5** (0.56 µL, 2.0 mM in DMF, 1.25 eq) was added to octreotide (30 µL, 30 µg/mL, 30 µM) in peptide buffer (40% MeCN, 2.5% DMF, 67.5% phosphate buffer [50 mM, pH 6.2]). The reaction mixture was incubated at 37 °C for 1 h. The samples were analysed by LCMS. Expected mass: 1185 Da. Observed mass: 1184 Da.

(a)

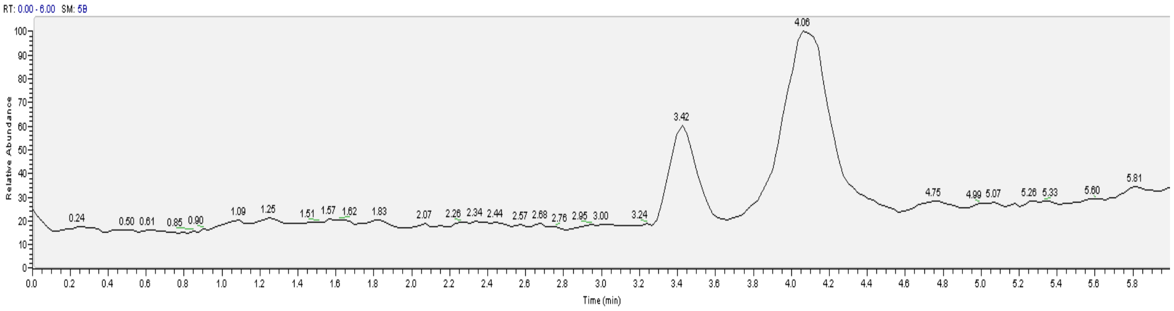

(b)

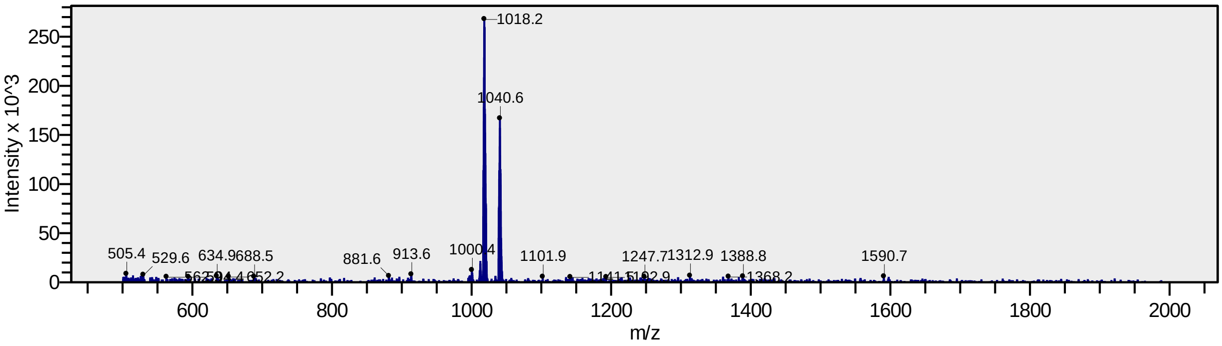

(c)

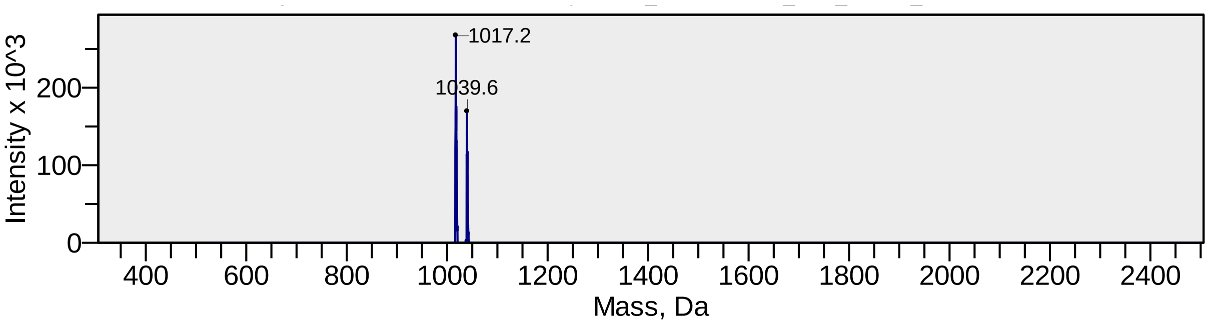

(d)

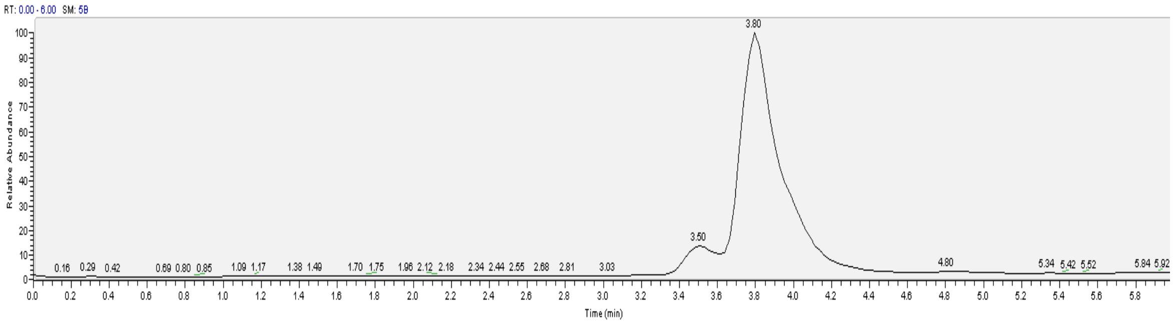

(e)

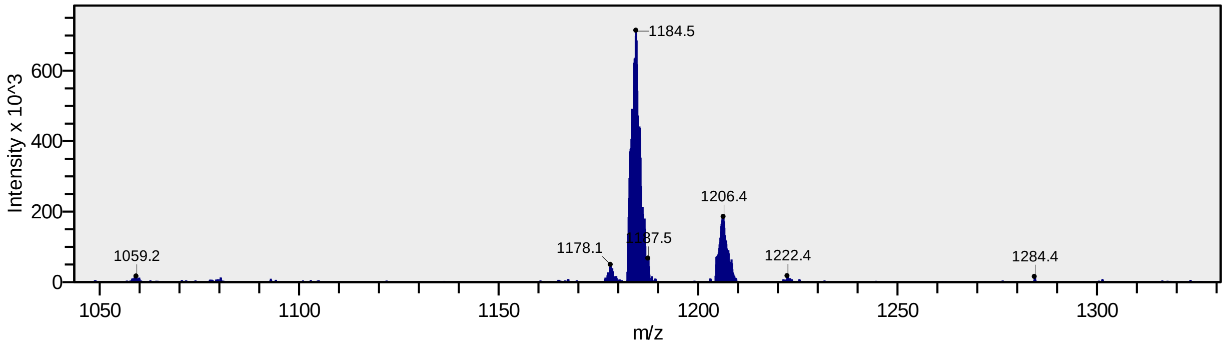

(f)

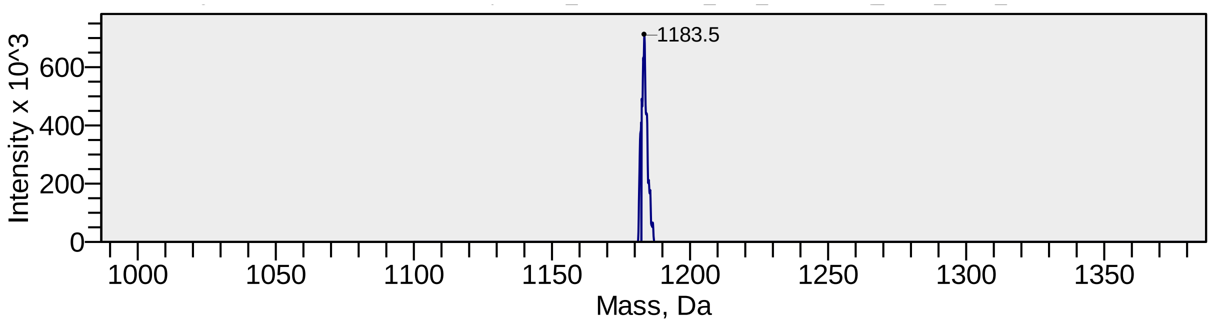

Figure **S24**. (a) TIC, (b) non-deconvoluted and (c) deconvoluted MS data for native octreotide; (d) TIC, (e) non-deconvoluted and (f) deconvoluted MS data for modified octreotide.

Reaction of Somatostatin peptide with pyridazinedione 5 (1.25 eq.)

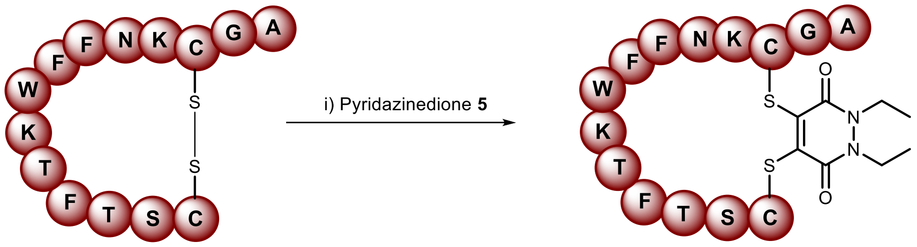

Pyridazinedione **5** (0.56 µL, 2.0 mM in DMF, 1.25 eq) was added to somatostatin (30 µL, 30 µg/mL, 30 µM) in peptide buffer (40% MeCN, 2.5% DMF, 67.5% phosphate buffer [50 mM, pH 6.2]) The reaction

mixture was incubated at 37 °C for 1 h. The samples were analysed by MALDI-TOF. Expected mass: 1,804 Da. Observed mass: 1,808 Da [M+H], 1,830 Da [M+Na], 1,847 Da [M+K].

(a)

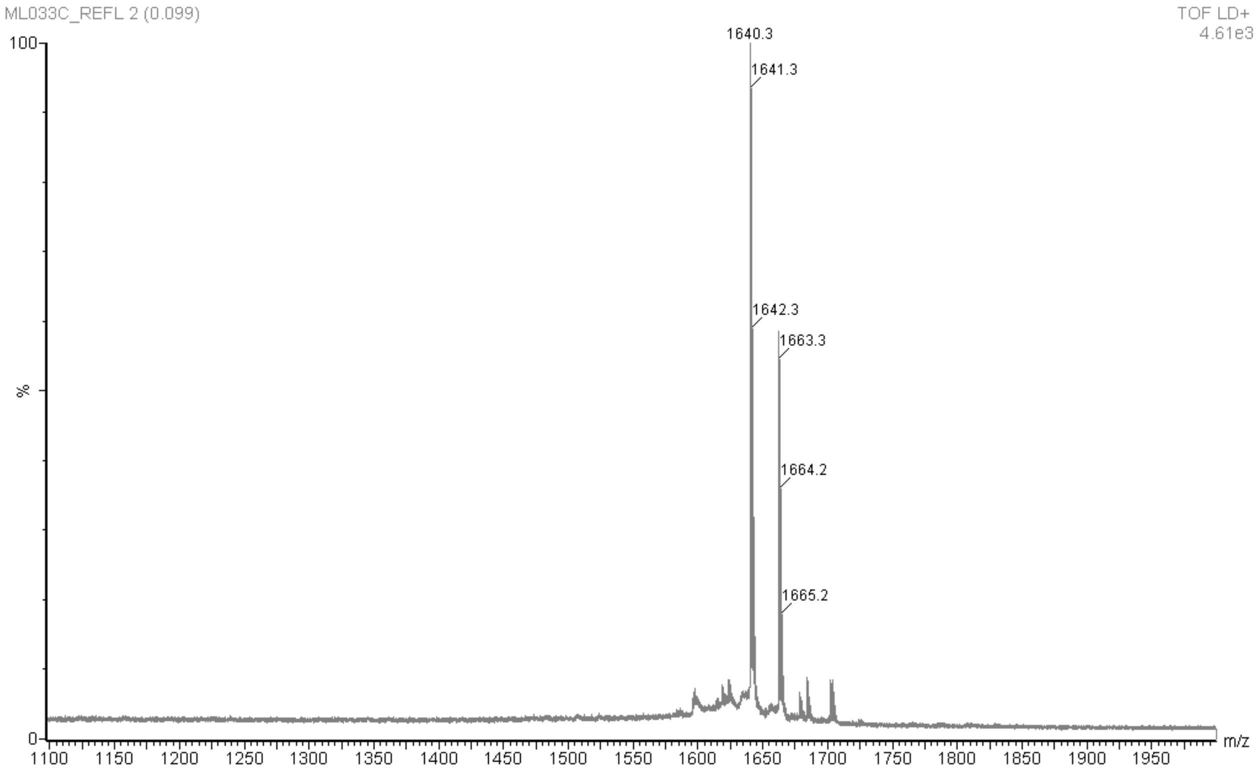

(b)

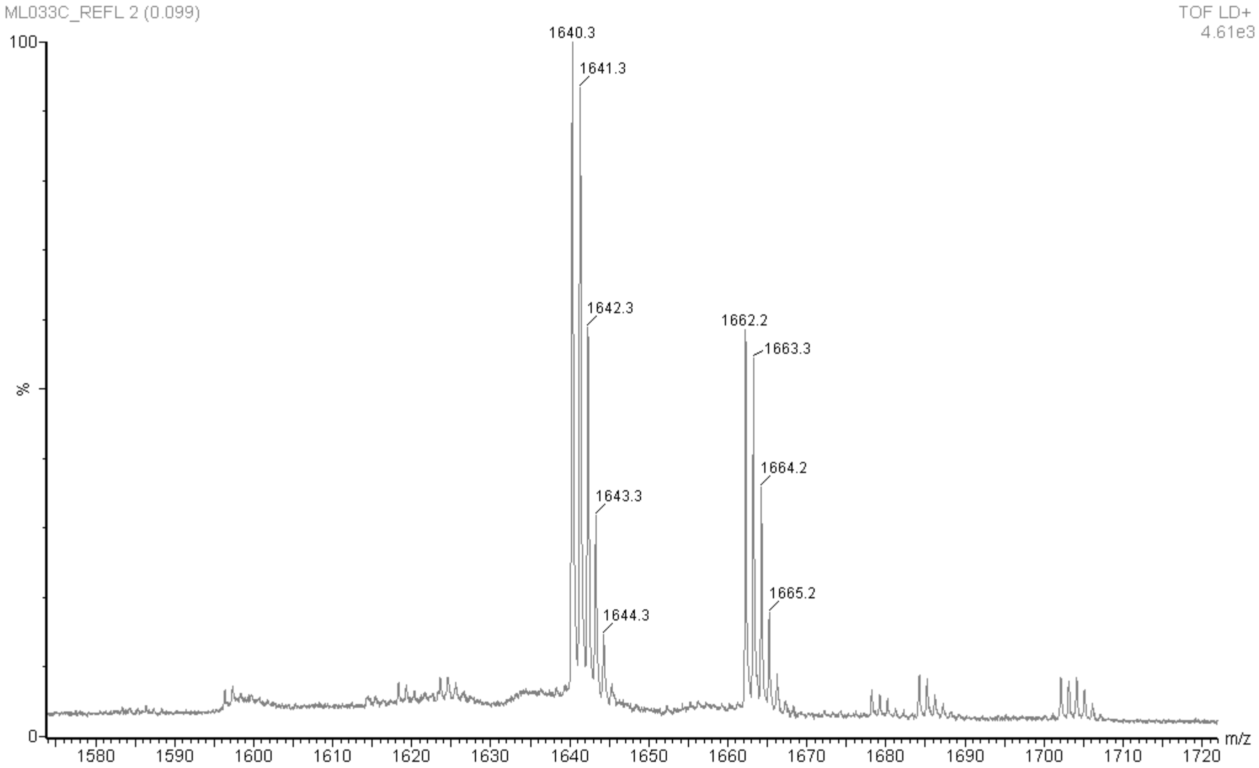

(c)

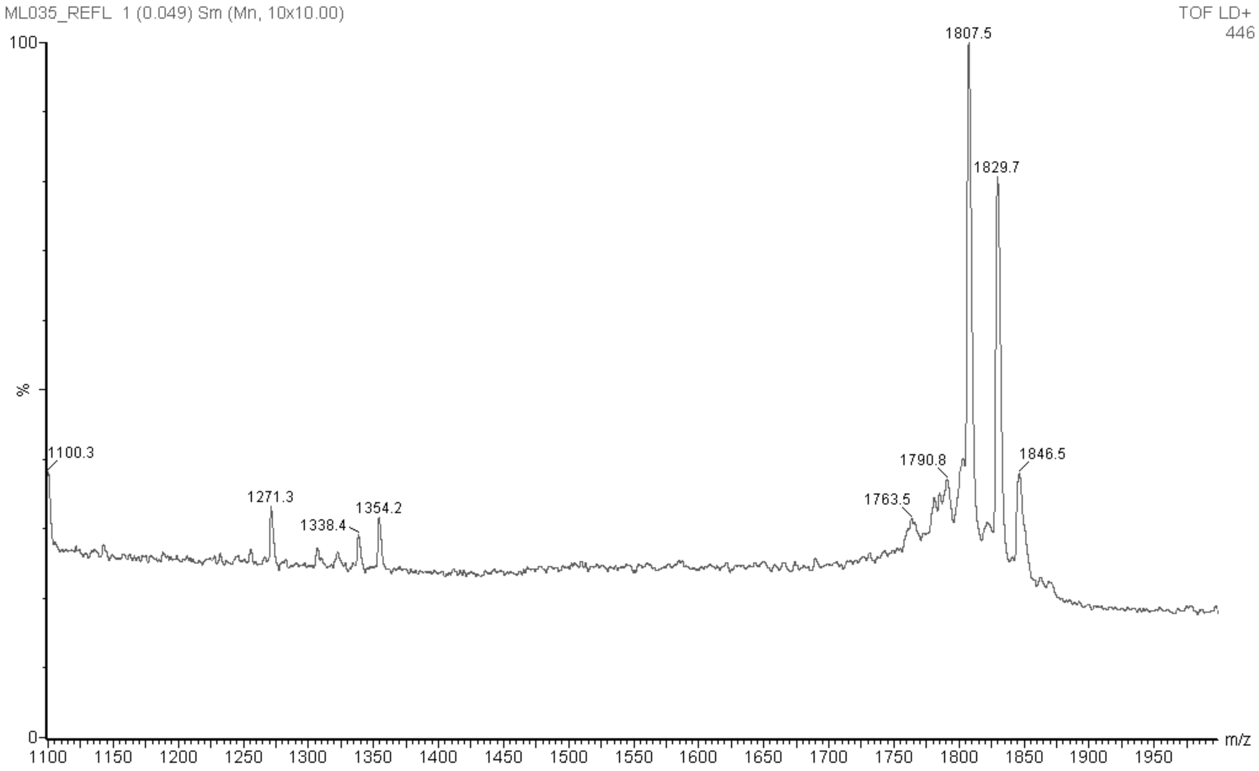
ML035_REFL 1 (0.049) Sm (Mn, 10x10.00)
TOF LD+
446
1807.5
1829.7
1846.5
1790.8
1763.5
1100.3
1271.3
1338.4
1354.2
100
%
0
m/z
1100
1150
1200
1250
1300
1350
1400
1450
1500
1550
1600
1650
1700
1750
1800
1850
1900
1950

(d)

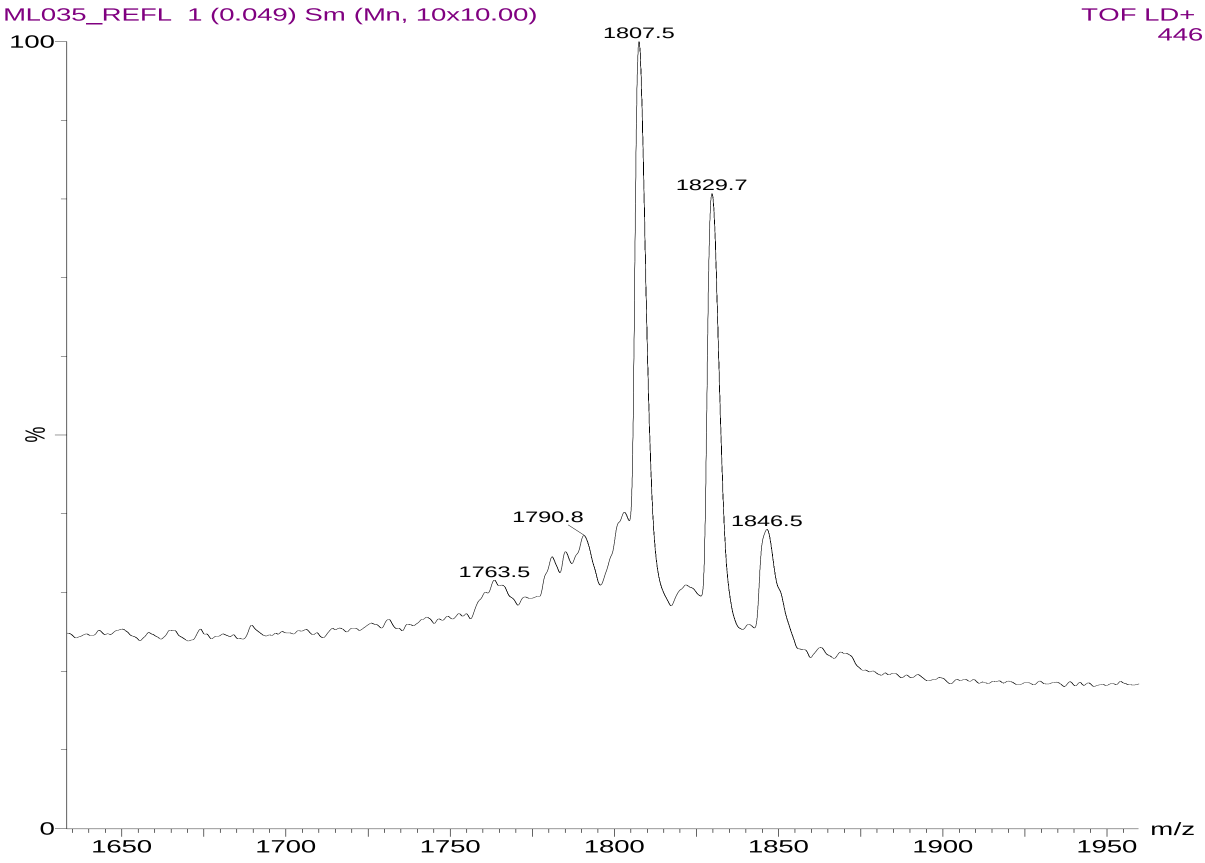
ML035_REFL 1 (0.049) Sm (Mn, 10x10.00)
TOF LD+
446
1807.5
1829.7
1790.8
1846.5
1763.5
100
%
0
m/z
1650
1700
1750
1800
1850
1900
1950

Figure **S25**. (a) MS data for native Somatostatin (1100–2000 m/z), (b) MS data for native Somatostatin (1580–2000 m/z), (c) MS data for modified Somatostatin (1100–2000 m/z) and (d) MS data for modified Somatostatin (1650–2000 m/z).

***In situ* reduction of Herceptin mAb and reaction with pyridazinedione 3 at 4 °C (25 eq.)**

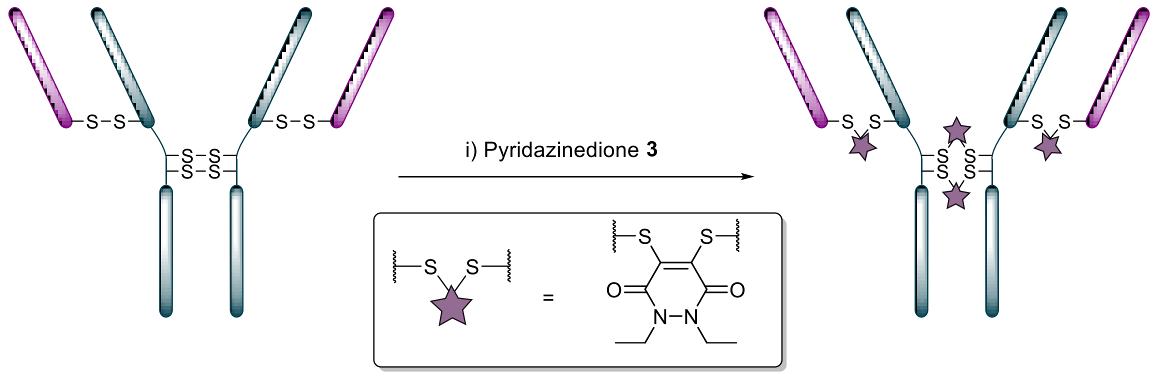

TCEP.HCl (2.3 µL, 20 mM in deionised water, 50 eq.) was added to Herceptin (30 µL, 4.4 mg/mL, 30 µM) in BBS (25 mM sodium borate, 25 mM NaCl, 0.5 mM EDTA, pH 8.0) which had been pretreated and stored at 4 °C for 1 h previously with pyridazinedione **3** (5.6 µL, 4 mM in DMF, 25 eq.). The reaction mixture was then stored at 4 °C for 15 h. Excess reagents were removed by repeated diafiltration into deionised water using VivaSpin sample concentrators (GE Healthcare, 10,000 MWCO). The samples were analysed by SDS-PAGE gel (see Figure **S37**. [lane 2]) and UV-vis spectroscopy was used to determine a PAR of 4.2. PAR was calculated according to that previously described.[3]

(a) 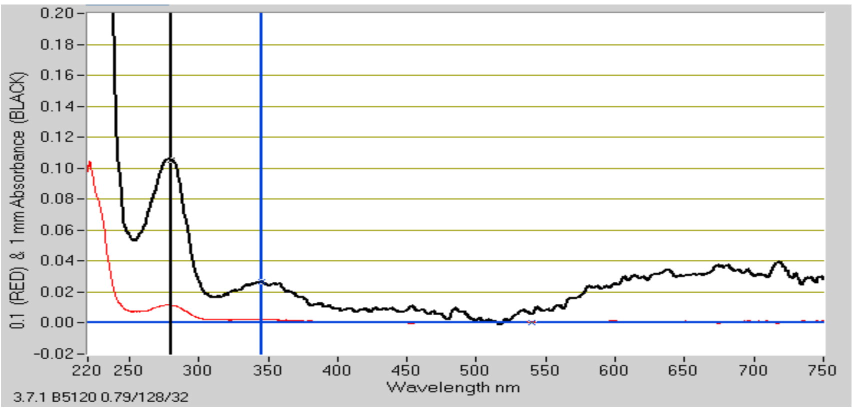

Figure **S26**. UV-vis data for Herceptin modified with pyridazinedione **3** at 4 °C *in situ*.

Portion-wise *in situ* reduction of Herceptin mAb and reaction with pyridazinedione 3 at 4 °C (25 eq.)

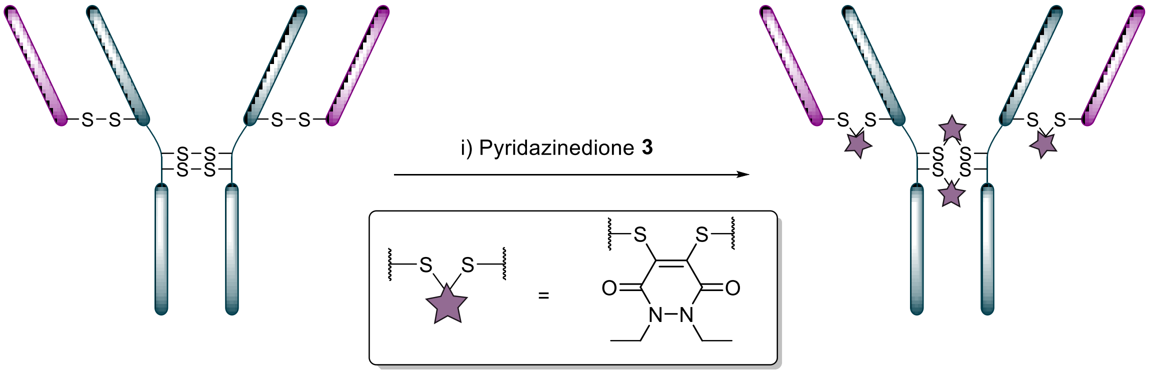

TCEP.HCl (1.2 µL, 0.2 mM in deionised water, 1.34 eq. [0.33 eq. per disulfide]) was added once every 0.5 h for 2.5 h (6 additions in total) to Herceptin (30 µL, 4.4 mg/mL, 30 µM) in BBS (25 mM sodium borate, 25 mM NaCl, 0.5 mM EDTA, pH 8.0) which had been pretreated and stored at 4 °C for 1 h previously with pyridazinedione **3** (5.6 µL, 4 mM in DMF, 25 eq.). The reaction mixture was then stored at 4 °C for 15 h. Excess reagents were removed by repeated diafiltration into deionised water using VivaSpin sample concentrators (GE Healthcare, 10,000 MWCO). The samples were analysed by SDS-PAGE gel (see Figure **S37**. [lane 3]) and UV-vis spectroscopy was used to determine a PAR of 4.0. PAR was calculated according to that previously described.[3]

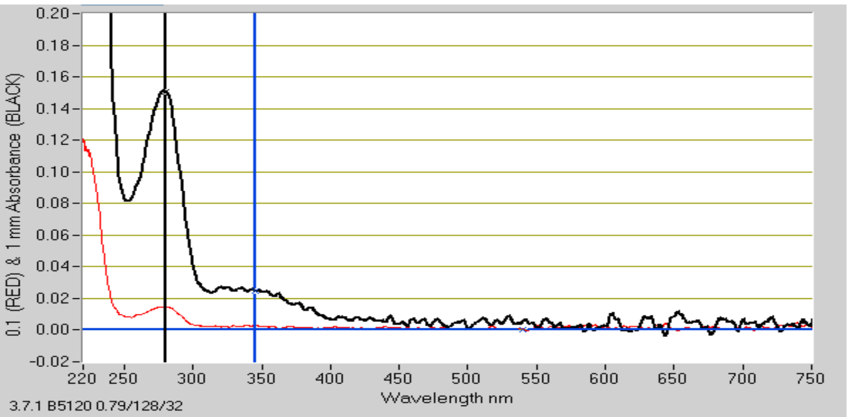

Figure **S27**. UV-vis data for Herceptin modified with pyridazinedione **3** *in situ* with portion-wise addition of TCEP.HCl.

Reaction of Herceptin mAb with pyridazinedione 5 at 4 °C (8 eq.)

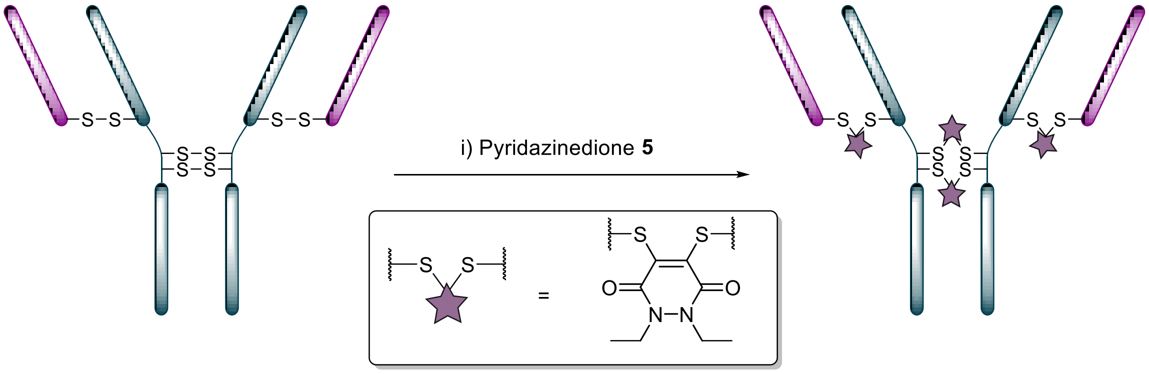

Pyridazinedione **5** (1.9 µL, 4.0 mM in DMF, 8 eq.) was added to Herceptin (30 µL, 4.4 mg/mL, 30 µM) in BBS (25 mM sodium borate, 25 mM NaCl, 0.5 mM EDTA, pH 8.0) which had been stored at 4 °C for 1 h previously. The reaction mixture was then stored at 4 °C for 15 h. Excess reagents were removed by repeated diafiltration into deionised water using VivaSpin sample concentrators (GE Healthcare, 10,000 MWCO). The samples were analysed by SDS-PAGE gel (see Figure **S37**. [lane 4]) and UV-vis spectroscopy was used to determine a PAR of 4.0. PAR was calculated according to that previously described.[3]

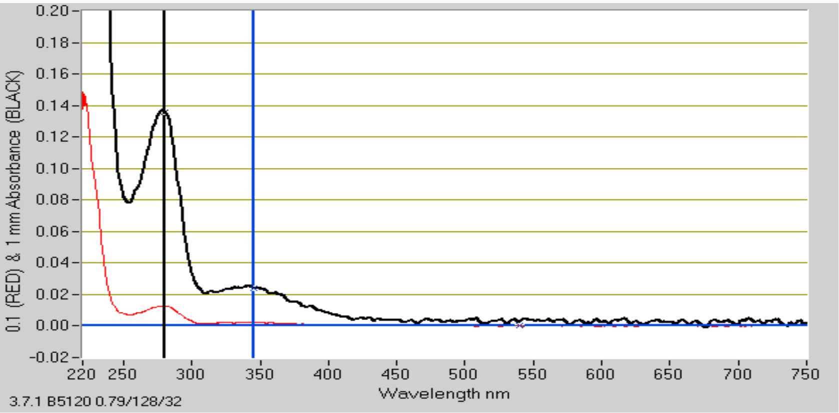

Figure **S28**. UV-vis data for Herceptin modified with pyridazinedione **5** at 4 °C.

***In situ* reduction of Herceptin mAb and reaction with pyridazinedione 3 at 37 °C (25 eq.)**

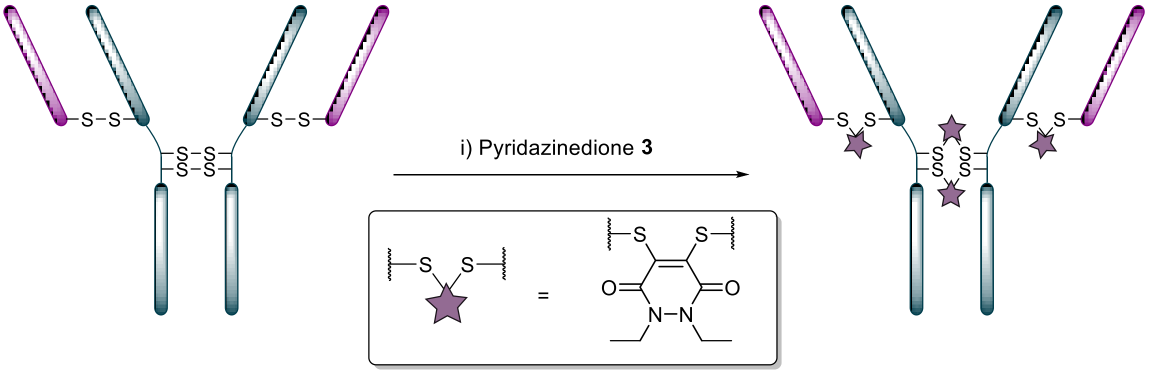

TCEP.HCl (2.3 µL, 20 mM in deionised water, 50 eq.) was added to Herceptin (30 µL, 4.4 mg/mL, 30 µM) in BBS (25 mM sodium borate, 25 mM NaCl, 0.5 mM EDTA, pH 8.0) which had been pretreated and stored at 37 °C for 1 h previously with pyridazinedione **3** (5.6 µL, 4.0 mM in DMF, 25 eq). The reaction mixture was then stored at 37 °C for 2 h. Excess reagents were removed by repeated diafiltration into deionised water using VivaSpin sample concentrators (GE Healthcare, 10,000 MWCO). The samples were analysed by SDS-PAGE gel (see Figure **S37**. [lane 5]) and UV-vis spectroscopy was used to determine a PAR of 4.2. PAR was calculated according to that previously described.[3]

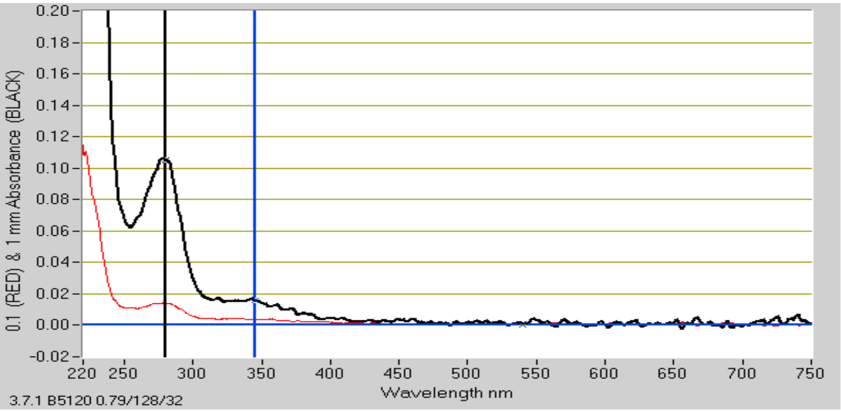

Figure **S29**. UV-vis data for Herceptin modified with pyridazinedione **3** at 37 °C *in situ*.

***In situ* reduction of Herceptin mAb and reaction with pyridazinedione 2 at 37 °C (25 eq.)**

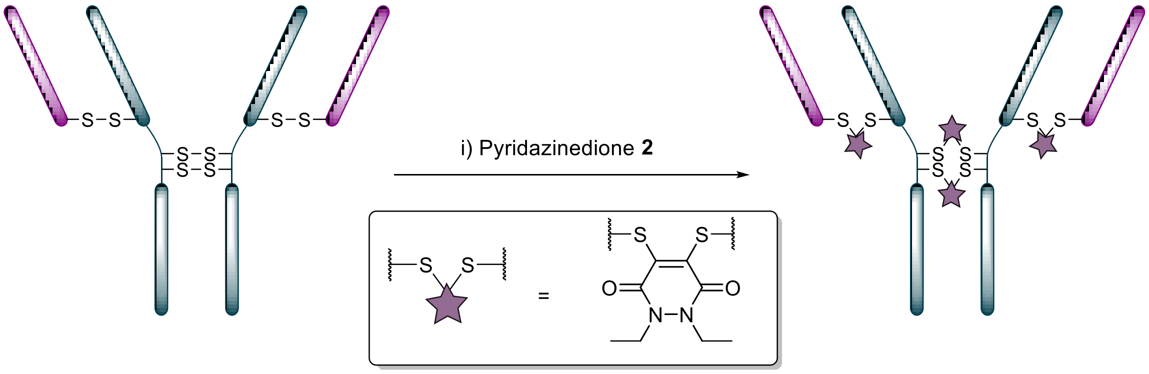

TCEP.HCl (2.3 µL, 20 mM in deionised water, 50 eq.) was added to Herceptin (30 µL, 4.4 mg/mL, 30 µM) in BBS (25 mM sodium borate, 25 mM NaCl, 0.5 mM EDTA, pH 8.0) which had been pretreated

and stored at 37 °C for 1 h previously with pyridazinedione **2** (5.6 µL, 4.0 mM in DMF, 25 eq). The reaction mixture was then stored at 37 °C for 2 h. Excess reagents were removed by repeated diafiltration into deionised water using VivaSpin sample concentrators (GE Healthcare, 10,000 MWCO). The samples were analysed by SDS-PAGE gel (see Figure **S38**. [lane 3]) and UV-vis spectroscopy was used to determine a PAR of 4.1. PAR was calculated according to that previously described.[3]

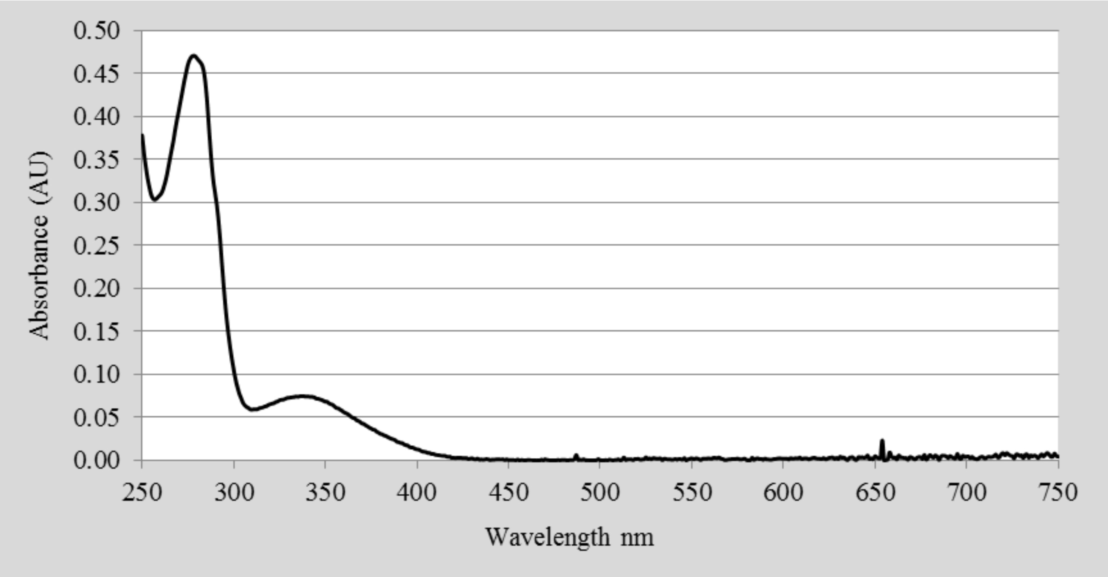

Figure **S30**. UV-vis data for Herceptin modified with pyridazinedione **2** at 37 °C *in situ*.

***In situ* reduction of Herceptin mAb and reaction with pyridazinedione 2 at 4 °C (25 eq.)**

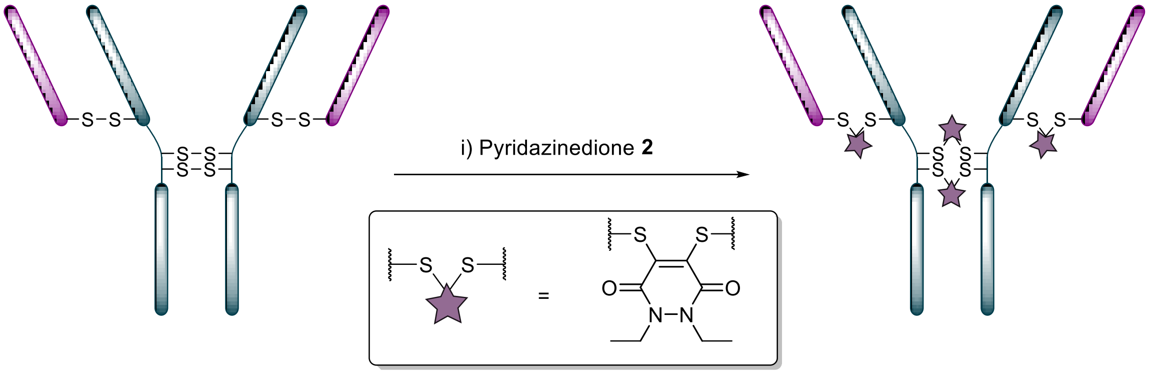

TCEP.HCl (2.3 µL, 20 mM in deionised water, 50 eq.) was added to Herceptin (30 µL, 4.4 mg/mL, 30 µM) in BBS (25 mM sodium borate, 25 mM NaCl, 0.5 mM EDTA, pH 8.0) which had been pretreated and stored at 4 °C for 1 h previously with pyridazinedione **2** (5.6 µL, 4 mM in DMF, 25 eq.). The reaction mixture was then stored at 4 °C for 15 h. Excess reagents were removed by repeated diafiltration into deionised water using VivaSpin sample concentrators (GE Healthcare, 10,000 MWCO). The samples were analysed by SDS-PAGE gel (see Figure **S38**. [lane 2]) and UV-vis spectroscopy was used to determine a PAR of 3.9.[3]

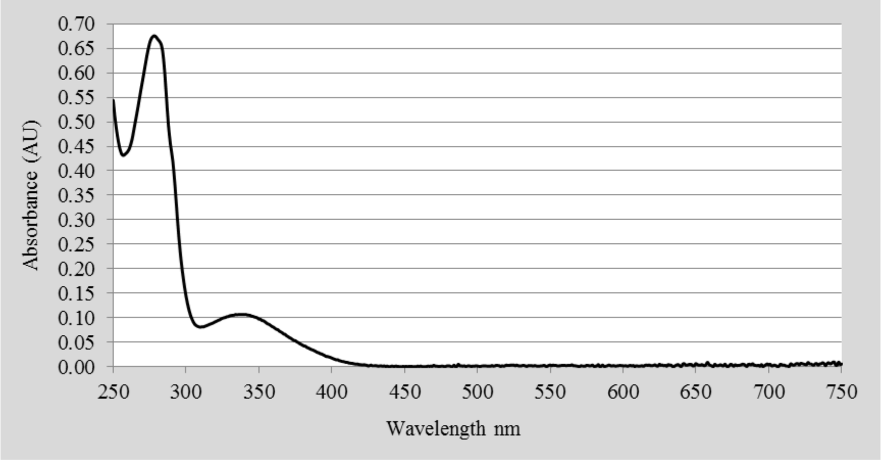

Figure **S31**. UV-vis data for Herceptin modified with pyridazinedione **2** at 4 °C *in situ*.

Control for untreated Fab fragment of Herceptin for experiments using pyridazinedione 7

During the course of this study the LC-MS device used (Thermo Scientific uPLC connected to MSQ Plus Single Quad Detector) underwent servicing and recalibration, this caused a slight change in observed mass for the untreated Fab fragment of Herceptin.

(a)

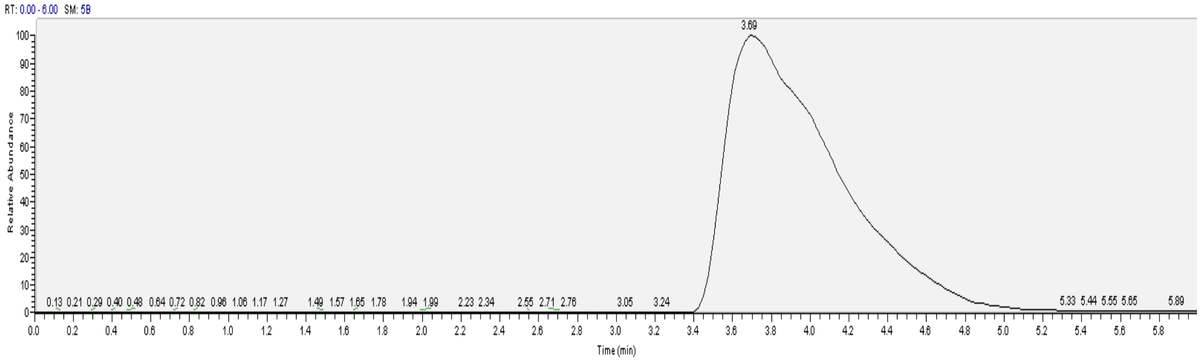

(b)

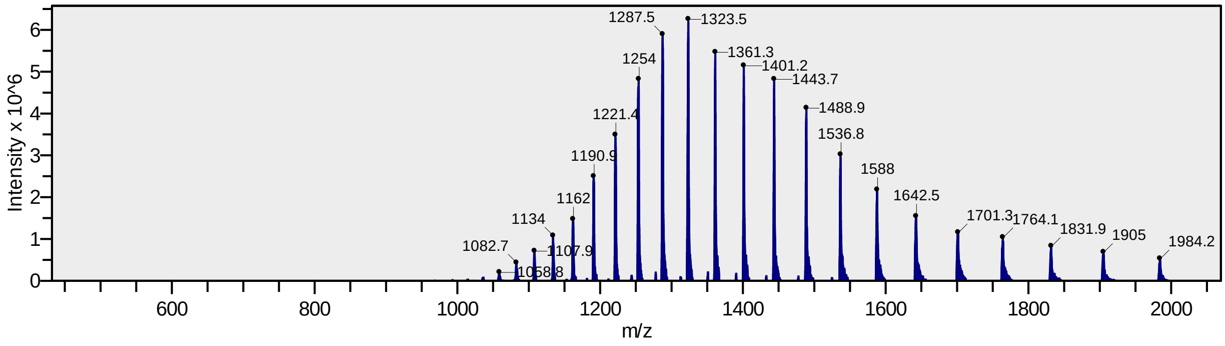

(c)

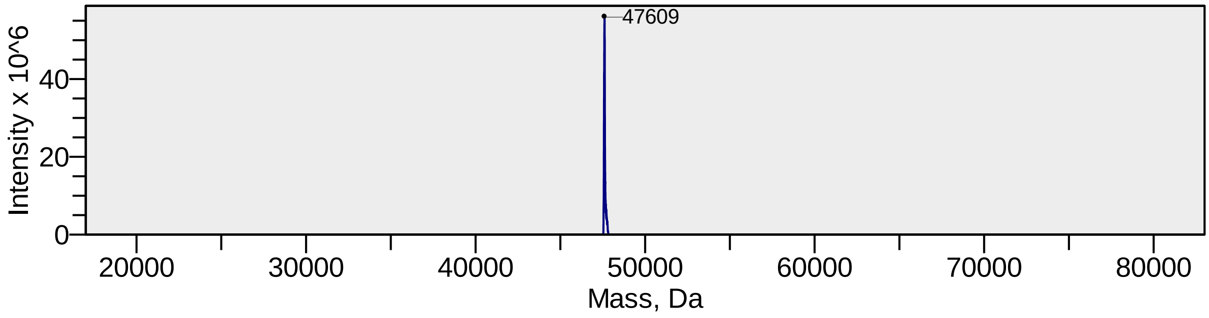

Figure **S32**. (a) TIC, (b) non-deconvoluted and (c) deconvoluted MS data for untreated Fab fragment of Herceptin for experiments using pyridazinedione **7**.

Reaction of Fab fragment of Herceptin with pyridazinedione 7 (1.25 eq) (Synthesis of bioconjugate 8)

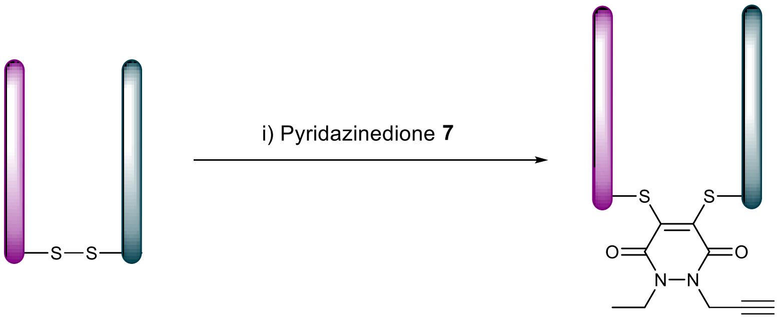

Pyridazinedione **7** (0.57 µL, 2.0 mM in DMF, 1.25 eq) was added to Fab fragment of Herceptin (30 µL, 1.4 mg/mL, 30 µM) in BBS (25 mM sodium borate, 25 mM NaCl, 0.5 mM EDTA, pH 8.0). The reaction mixture was incubated at 37 °C for 1 h. Excess reagents were removed by repeated diafiltration into deionised water using VivaSpin sample concentrators (GE Healthcare, 10,000 MWCO). The samples were analysed by LCMS. Expected mass: 47,785 Da. Observed mass: 47,787 Da.

(a)

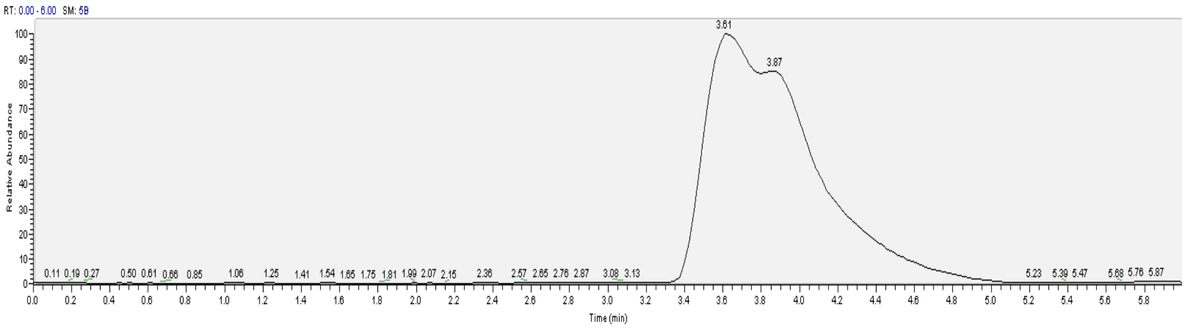

(b)

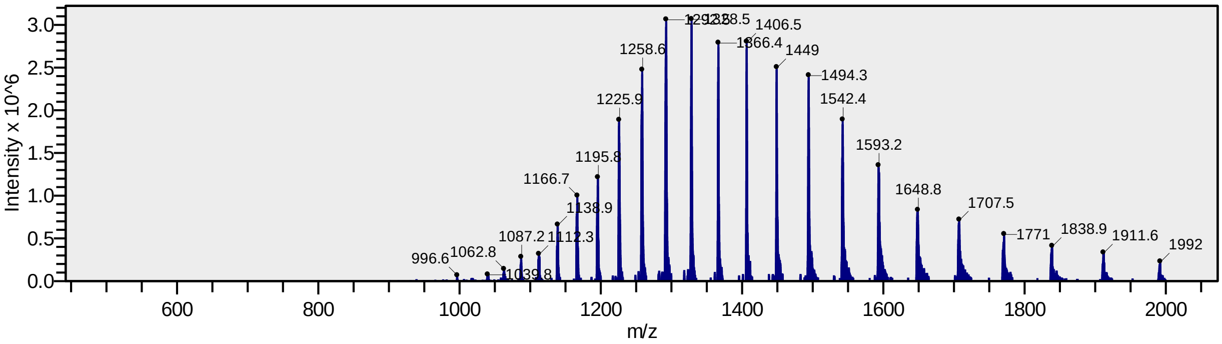

(c)

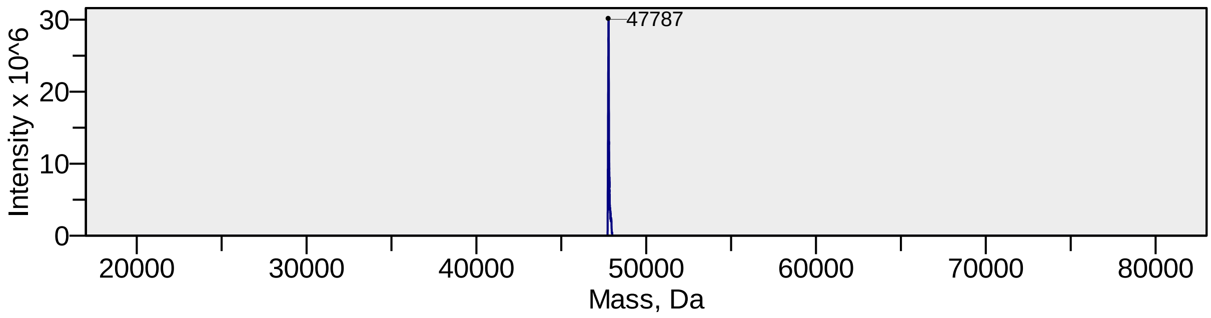

Figure **S33**. (a) TIC, (b) non-deconvoluted and (c) deconvoluted MS data for bioconjugate **8**.

Reaction of Fab-Her bioconjugate 8 with Cy5-azide (Synthesis of bioconjugate 9a)

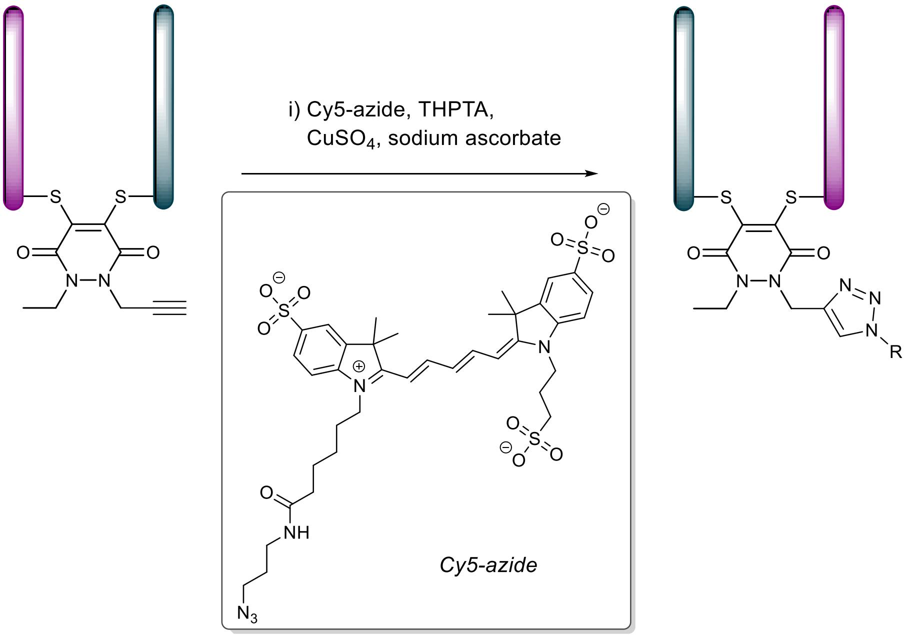

Cy5-azide (1.0 µL, 2.0 mM in water, 2 eq), $CuSO_4$ (1.0 µL, 2.0 mM in water, 2 eq), tris(3-hydroxypropyltriazolylmethyl)amine (THPTA) (1.0 µL, 10 mM in water, 10 eq) and sodium ascorbate (5.0 µL, 0.10 M in water, 500 eq) were added in series to bioconjugate **8** (20 µL, 2.4 mg/mL, 50 µM) in BBS (25 mM sodium borate, 25 mM NaCl, pH 8.0). The reaction mixture was stored at 21 °C for 1.5 h. The excess reagents were then removed by repeated diafiltration into fresh PBS with 2 mM EDTA (to remove residual copper ions) using VivaSpin sample concentrators (GE Healthcare, 10,000 MWCO). Following this, the buffer salts were removed by repeated diafiltration into deionised water using VivaSpin sample concentrators (GE Healthcare, 10,000 MWCO). The samples were then analysed by LCMS. Expected mass: 48,615 Da. Observed mass: 48,613 Da.

(a)

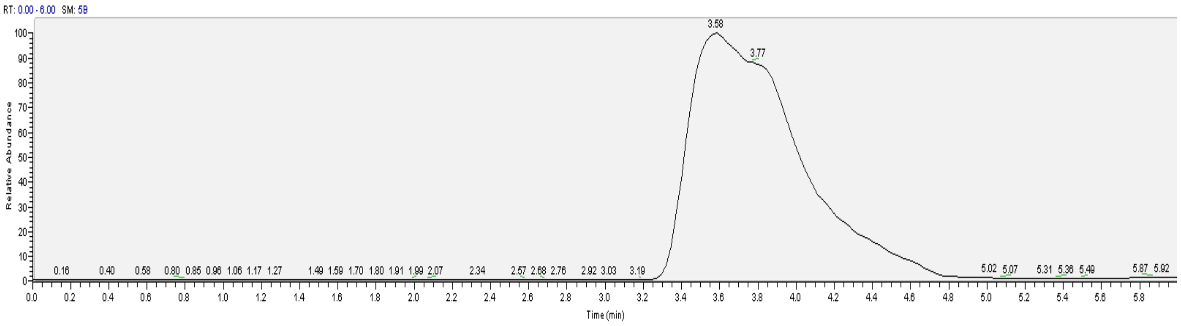

(b)

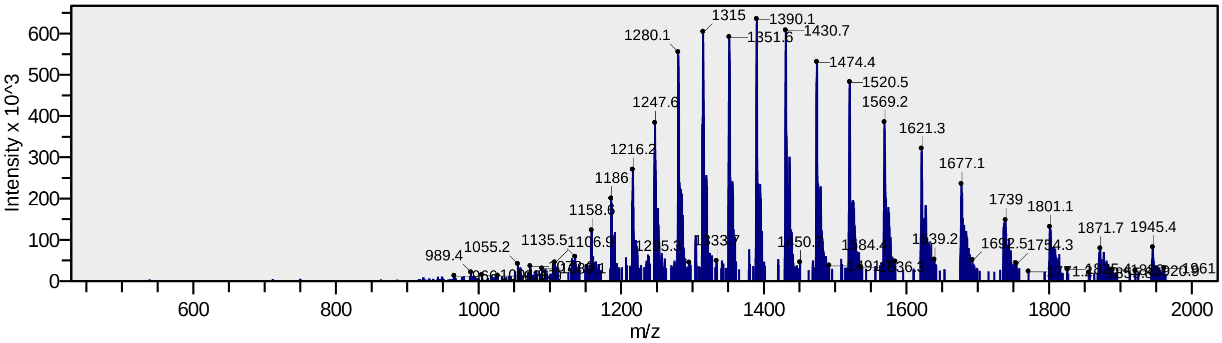

(c)

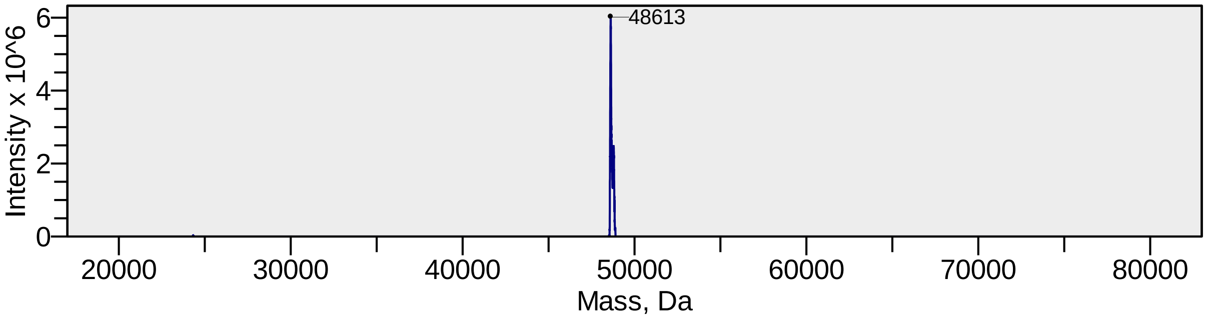

Figure **S34**. (a) TIC, (b) non-deconvoluted and (c) deconvoluted MS data for bioconjugate **9a**.

Reaction of Fab-Her bioconjugate 8 with Doxorubicin-azide[3] (Synthesis of bioconjugate 9b)

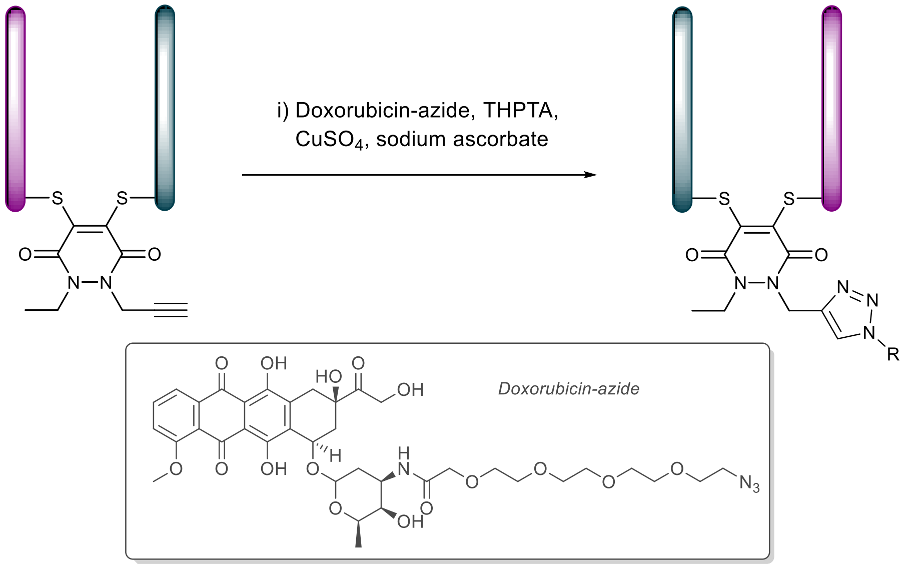

Doxorubicin-azide[3] (1.0 µL, 2.0 mM in water, 2 eq), $CuSO_4$ (1.0 µL, 2.0 mM in water, 2 eq), tris(3-hydroxypropyltriazolylmethyl)amine (THPTA) (1.0 µL, 10 mM in water, 10 eq) and sodium ascorbate (5.0 µL, 0.10 M in water, 500 eq) were added in series to bioconjugate **8** (20 µL, 2.4 mg/mL, 50 µM) in BBS (25 mM sodium borate, 25 mM NaCl, pH 8.0). The reaction mixture was stored at 21 °C for 1.5 h. The excess reagents were then removed by repeated diafiltration into fresh PBS with 2 mM EDTA (to remove residual copper ions) using VivaSpin sample concentrators (GE Healthcare, 10,000 MWCO). Following this, the buffer salts were removed by repeated diafiltration into deionised water using VivaSpin sample concentrators (GE Healthcare, 10,000 MWCO). The samples were then analysed by LCMS. Expected mass: 48,587 Da. Observed mass: 48,586 Da.

(a)

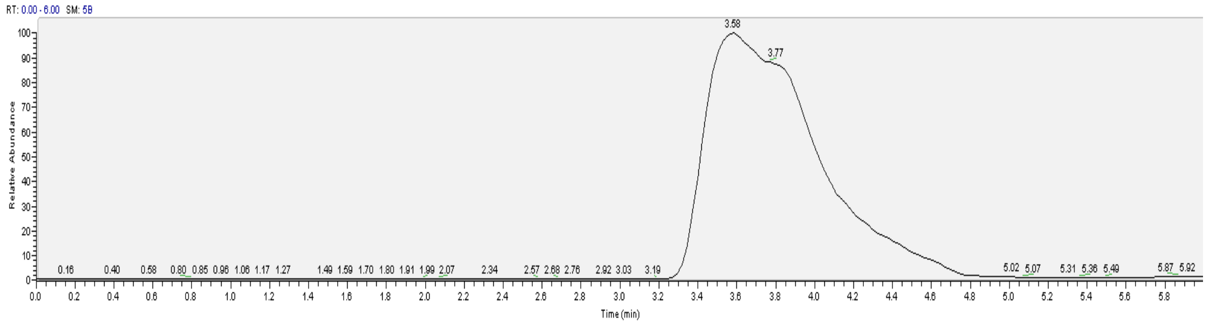

(b)

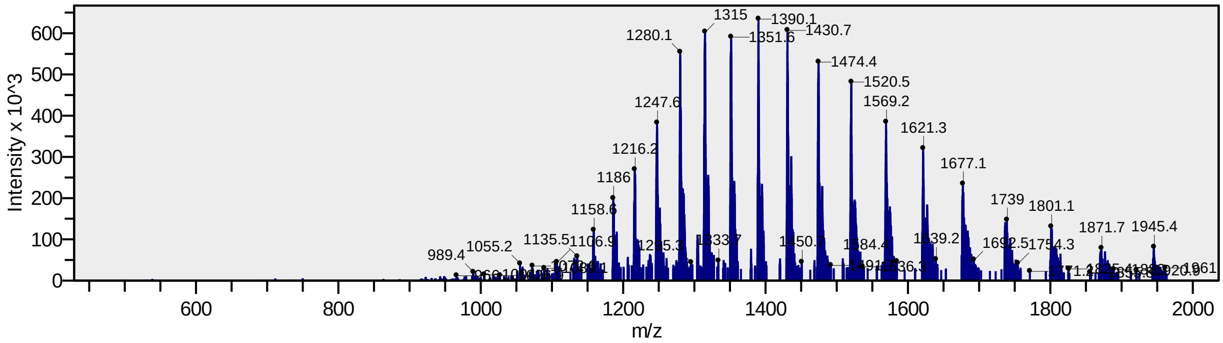

(c)

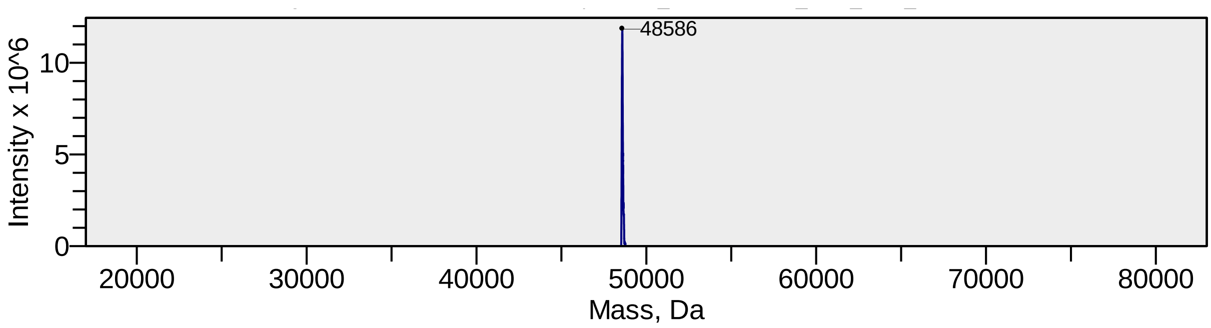

Figure **S35**. (a) TIC, (b) non-deconvoluted and (c) deconvoluted MS data for bioconjugate **9b**.

Reaction of Fab-Her bioconjugate 8 with AlexaFluor 488®-azide (Synthesis of bioconjugate 9c)

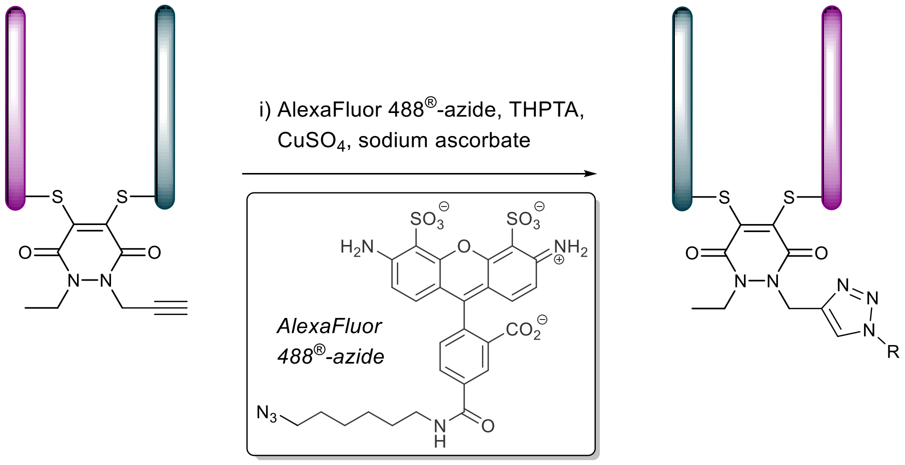

AlexaFluor 488®-azide (1.0 µL, 2.0 mM in water, 2 eq), $CuSO_4$ (1.0 µL, 2.0 mM in water, 2 eq), tris(3-hydroxypropyltriazolylmethyl)amine (THPTA) (1.0 µL, 10 mM in water, 10 eq) and sodium ascorbate (5.0 µL, 0.10 M in water, 500 eq) were added in series to bioconjugate **8** (20 µL, 2.4 mg/mL, 50 µM) in BBS (25 mM sodium borate, 25 mM NaCl, pH 8.0). The reaction mixture was stored at 21 °C for 1.5 h. The excess reagents were then removed by repeated diafiltration into fresh PBS with 2 mM EDTA (to remove residual copper ions) using VivaSpin sample concentrators (GE Healthcare, 10,000 MWCO). Following this, the buffer salts were removed by repeated diafiltration into deionised water using VivaSpin sample concentrators (GE Healthcare, 10,000 MWCO). The samples were then analysed by LCMS. Expected mass: 48,442 Da. Observed mass: 48,447 Da.

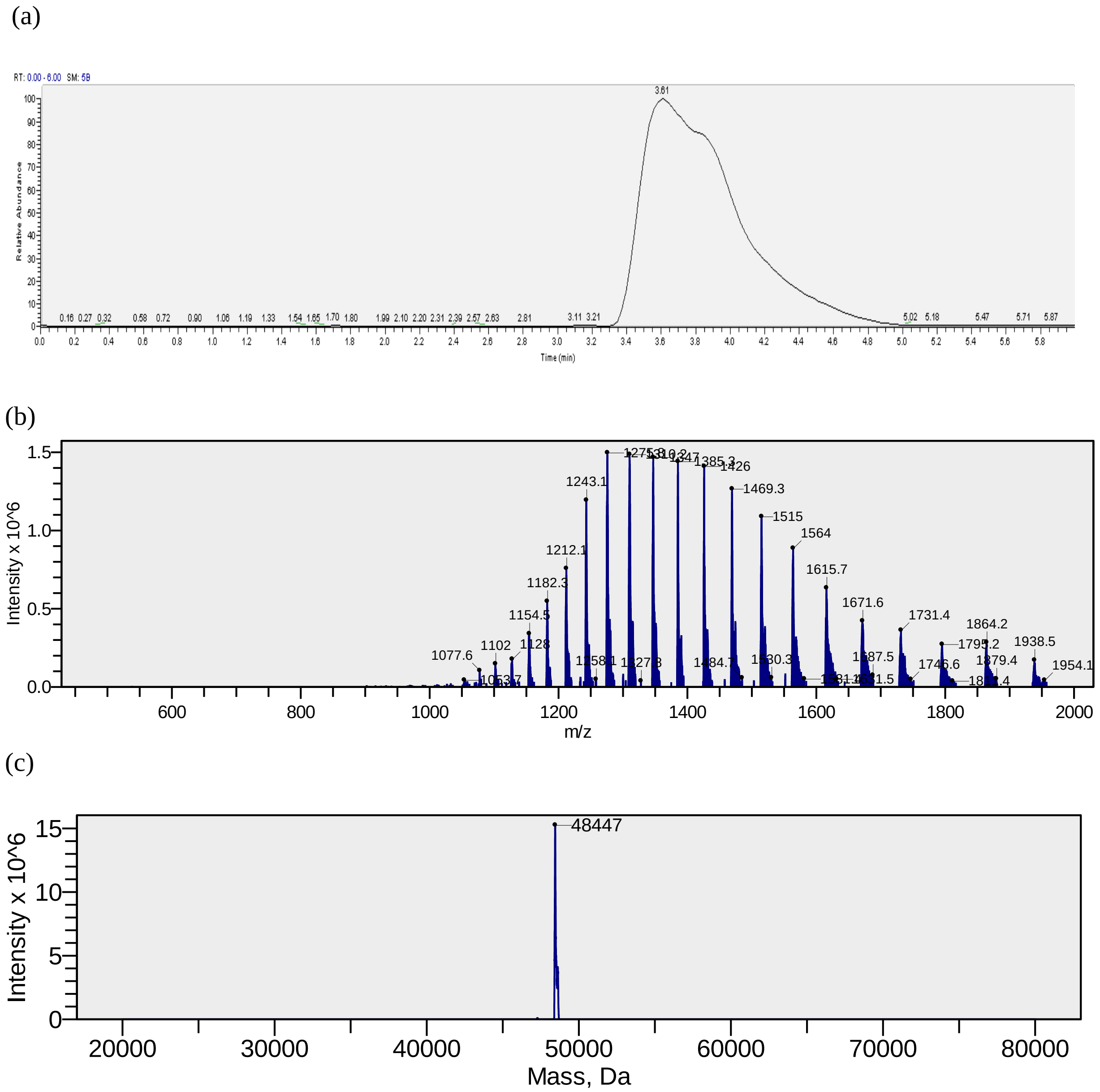

Figure **S36**. (a) TIC, (b) non-deconvoluted and (c) deconvoluted MS data for bioconjugate **9c**.

SDS-PAGE gel for Herceptin modification with pyridazinediones 3 and 5

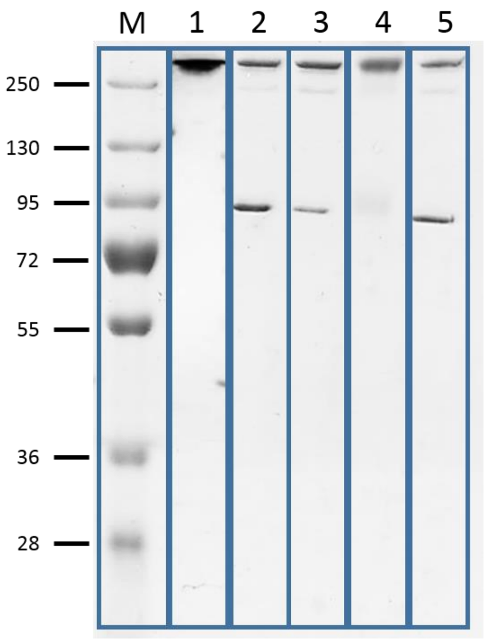

Figure **S37**. Comparison of traditional methods for Herceptin modification against the use of pyridazinedione **5**. M) Molecular weight marker. 1) Untreated Herceptin. 2) *In situ* reduction of Herceptin mAb and reaction with pyridazinedione **3** at 4 °C (25 eq.). 3) Portion-wise *in situ* reduction of Herceptin mAb and reaction with pyridazinedione **3** at 4 °C (25 eq.). 4) Reaction of Herceptin mAb with pyridazinedione **5** at 4 °C (8 eq.). 5) *In situ* reduction of Herceptin mAb and reaction with pyridazinedione **3** at 37 °C (25 eq.)

SDS-PAGE gel for Herceptin modification with pyridazinedione 2

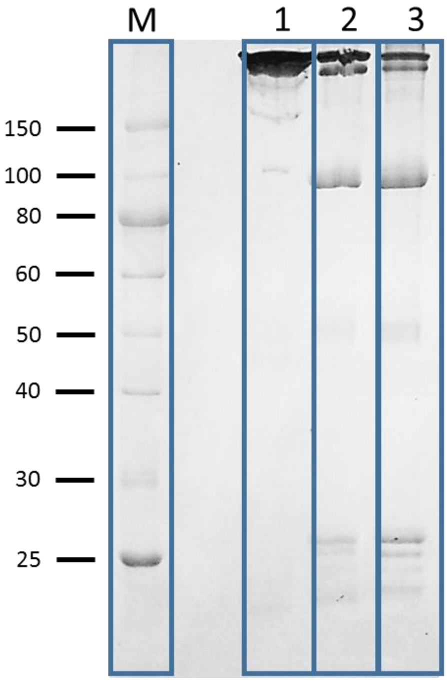

Figure **S38**. Further demonstration of traditional methods for Herceptin modification. M) Molecular weight marker. 1) Untreated Herceptin. 2) *In situ* reduction of Herceptin mAb and reaction with pyridazinedione **2** at 4 °C (25 eq.). 3) *In situ* reduction of Herceptin mAb and reaction with pyridazinedione **2** at 37 °C (25 eq.).

SDS-PAGE gel for Fab fragment of Herceptin and conjugates 8, 9a, 9b and 9c

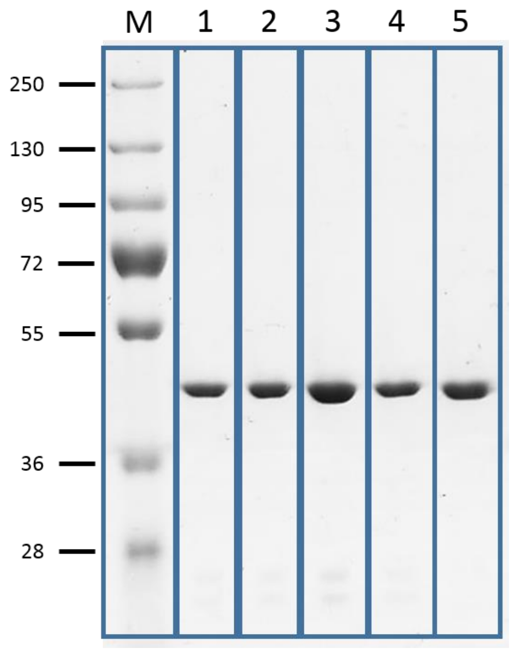

Figure **S39** Distribution of Fab-Her bioconjugates **8**, **9a**, **9b** and **9c** against unmodified Fab-Her fragment. M) Molecular weight marker. 1) Unmodified Fab-Her fragment. 2) Bioconjugate **8**. 3) Bioconjugate **9a**. 4) Bioconjugate **9b**. 5) Bioconjugate **9c**.

Activity by enzyme-linked immunosorbent assay (ELISA)

Binding affinity to HER2 receptor was determined by ELISA. A 96-well plate was coated overnight at 4 °C with HER2 (100 µL of a 0.25 µg·mL^{-1} solution in PBS), including coating one row of wells with PBS only for negative controls. Next, coating solutions were removed and each well washed with PBS twice. Then, the wells were coated with a 1% BSA solution in PBS (200 µL) for 1 h at 21 °C. Then, the wells were washed with PBS three times. Solutions Fab fragment bioconjugates **9a**, **9b** and **9c** in PBS with the following dilution series: 23 nM, 7.8 nM, 2.6 nM, 0.86 nM, 0.29 nM and 0.10 nM were prepared. Wells were coated with the dilution series solutions, including a PBS only and unmodified Fab fragment at 23 nM in the absence of HER2 as negative controls, and incubated for 2 h at room temperature. Then, the solutions were removed and the wells washed with 0.1% Tween 20 in PBS twice and with PBS three times. Detection antibody (100 µL of anti-human IgG, Fab-specific-HRP solution, prepared by taking 4 µL of a 1:5000 diluted solution and further diluting with 20 mL of PBS) was added and incubated for 1 h at room temperature. Then, the solutions were removed and the wells washed with 0.1% Tween 20 in PBS twice and with PBS three times. Finally, an OPD solution (100 µL of 0.5 mg·mL^{-1} OPD in phosphate-citrate buffer with sodium perborate, prepared by dissolving 1 capsule in 100 mL water) was added to each well. After *ca.* 2 min the reaction was stopped through addition of 4 M HCl (50 µL). Absorbance was measured at 490 nm. Absorbance was corrected by subtracting average of negative controls. See Figure **S40** and **S44**.

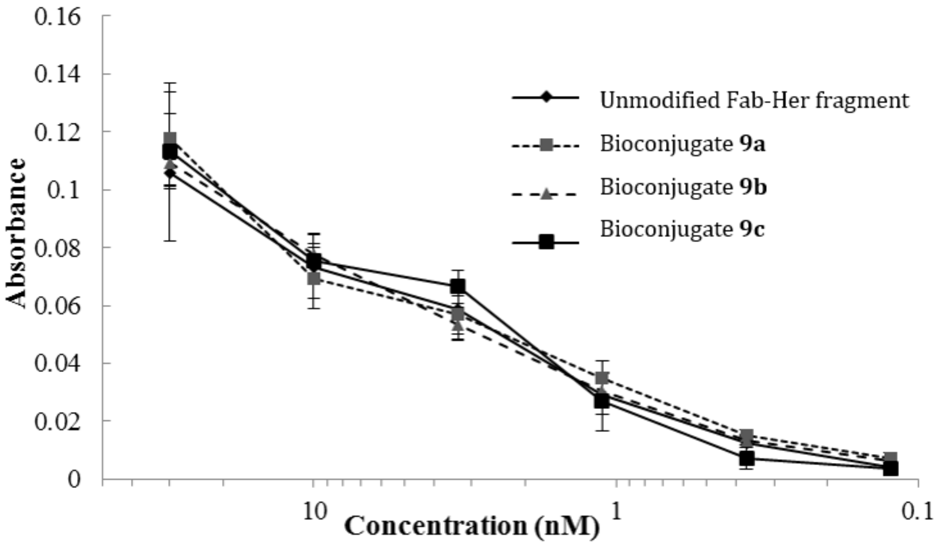

Figure **S40**. Binding activity of Fab-Her bioconjugates **9a**, **9b** and **9c** against untreated Fab fragment of Herceptin.

Reaction of Herceptin mAb with pyridazinedione 7 at 4 °C (8 eq.) for the formation of Her-PD7

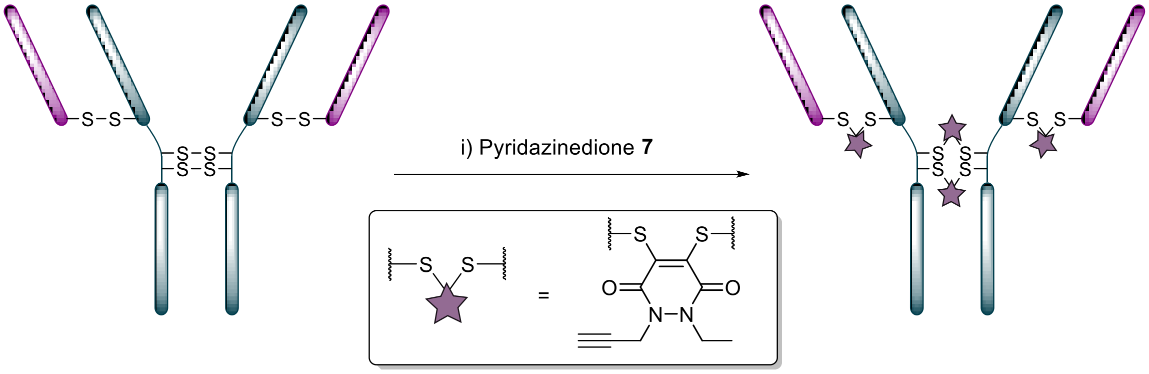

Pyridazinedione **7** (1.9 µL, 4.0 mM in DMF, 8 eq.) was added to Herceptin (30 µL, 4.4 mg/mL, 30 µM) in BBS (25 mM sodium borate, 25 mM NaCl, 0.5 mM EDTA, pH 8.0) which had been stored at 4 °C for 1 h previously. The reaction mixture was then stored at 4 °C for 16 h. Excess reagents were removed by repeated diafiltration into deionised water using VivaSpin sample concentrators (GE Healthcare, 10,000 MWCO). The samples were analysed by SDS-PAGE gel (see Figure **S43**. [lane 2]) and UV-vis spectroscopy was used to determine a PAR of 4.1. PAR was calculated according to that previously described.[3]

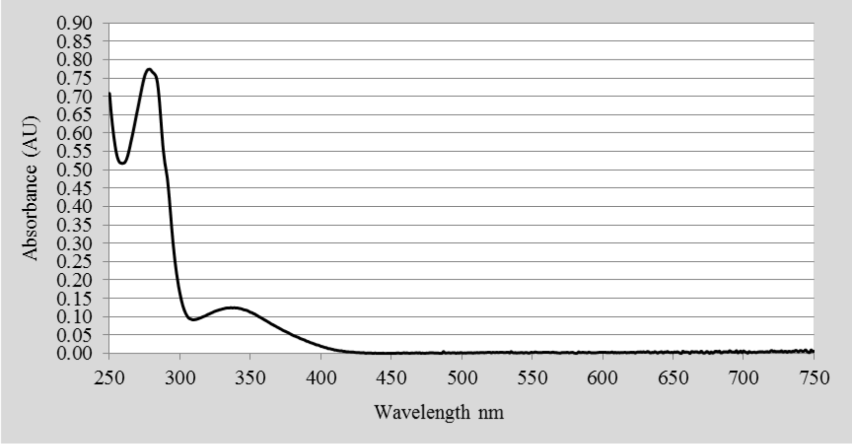

Figure **S41**. UV-vis data for Herceptin modified with pyridazinedione **7** (*i.e.* Her-PD**7**) at 4 °C.

Reaction of Her-PD7 with Doxorubicin-azide[3] for the formation of Her-PD7-Dox

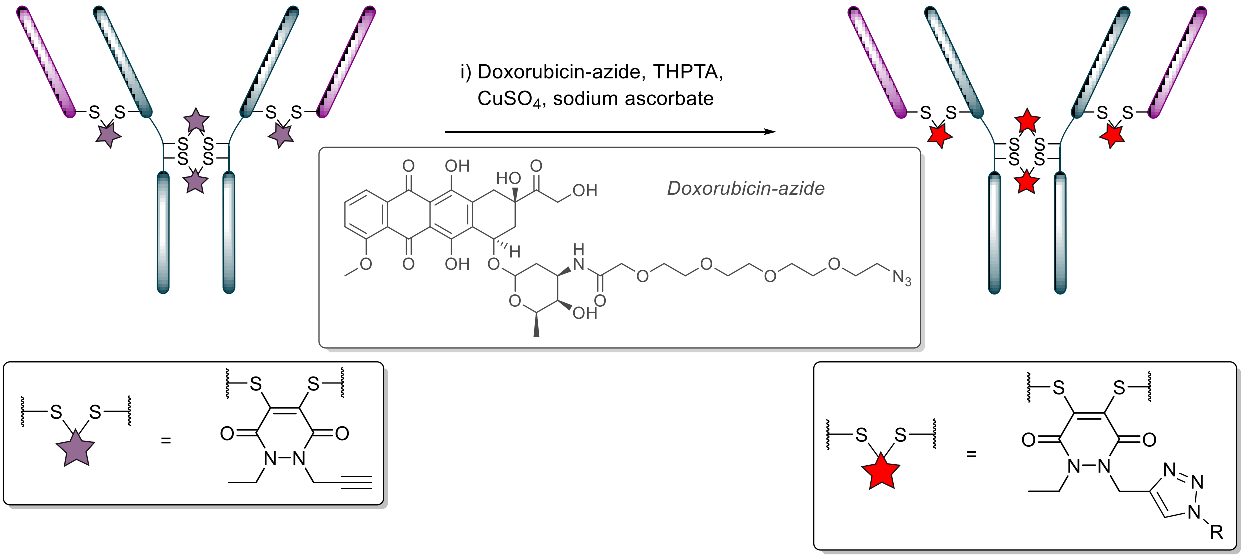

Doxorubicin-azide (1.0 µL, 2.0 mM in water, 2 eq), $CuSO_4$ (1.0 µL, 2.0 mM in water, 2 eq), tris(3-hydroxypropyltriazolylmethyl)amine (THPTA) (1.0 µL, 10 mM in water, 10 eq) and sodium ascorbate (5.0 µL, 0.10 M in water, 500 eq) were added in series to Her-PD**7** (20 µL, 2.4 mg/mL, 50 µM) in BBS (25 mM sodium borate, 25 mM NaCl, pH 8.0). The reaction mixture was stored at 21 °C for 1.5 h. The excess reagents were then removed by repeated diafiltration into fresh PBS with 2 mM EDTA (to remove residual copper ions) using VivaSpin sample concentrators (GE Healthcare, 10,000 MWCO). Following this, the buffer salts were removed by repeated diafiltration into deionised water using VivaSpin sample concentrators (GE Healthcare, 10,000 MWCO). The samples were analysed by SDS-PAGE gel (see Figure **S43**. [lane 3]) and UV-vis spectroscopy was used to determine a DAR of 4.0. DAR was calculated according to that previously described.[3]

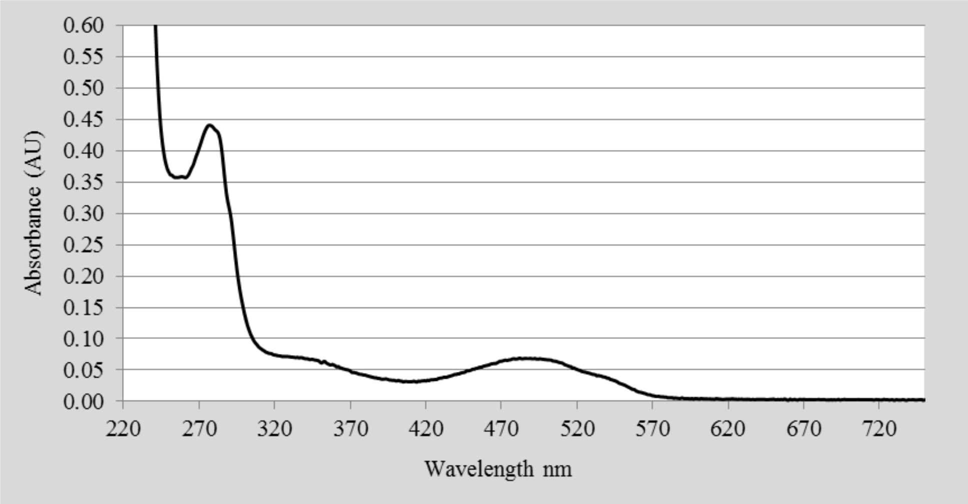

Figure **S42**. UV-vis data for Her-PD**7**-Dox conjugate.

SDS-PAGE gel for Herceptin modification with pyridazinedione 7 and Dox conjugate

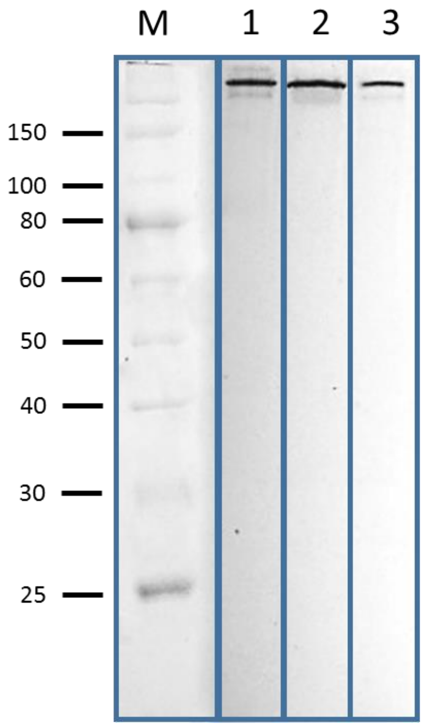

Figure **S43**. Distribution of Her-PD**7** and Her-PD**7**-Dox bioconjugates against unmodified native Herceptin. M) Molecular weight marker. 1) Untreated Herceptin. 2) Reaction of Herceptin mAb with pyridazinedione **7** at 4 °C (8 eq.) (*i.e.* Her-PD**7**). 3) Her-PD7-Dox conjugate.

ELISA for Her-PD7-Dox bioconjugate against native Herceptin

Carried out according to procedure detailed for bioconjugates **9a-c**.

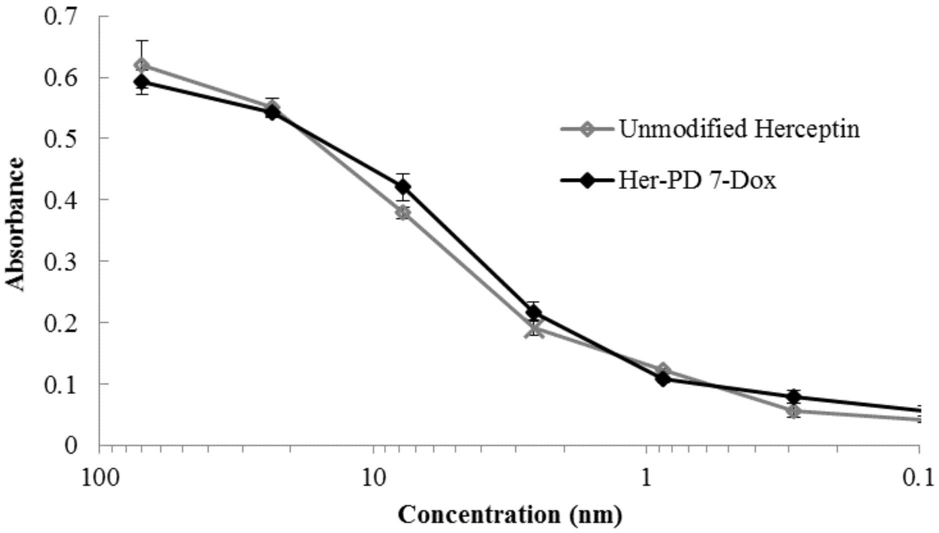

Figure **S44**. Binding activity of Her-PD**7**-Dox against native unmodified Herceptin.

References

1. V. Chudasama, M. E. B. Smith, F. F. Schumacher, D. Papaioannou, G. Waksman, J. R. Baker and S. Caddick, *Chem. Commun.*, 2011, **47**, 8781-8783.

2. K. Gorska, A. Manicardi, S. Barluenga and N. Winssinger, *Chem. Commun.*, 2011, **47**, 4364-4366.

3. A. Maruani, M. E.B. Smith, E. Miranda, K. A. Chester, V. Chudasama and S. Caddick, *Nat. Commun.*, 2015, **6**, 6645.
